# [1,2]-Carbon to Carbon Silyl Migration for Accessing α‑Silyl Alkanals from α‑Hydroxy Allyl Silanes

**DOI:** 10.1021/acs.joc.6c00817

**Published:** 2026-06-15

**Authors:** Darshika Singh, Emmanuel W. Maloba, Robert E. Maleczka

**Affiliations:** Department of Chemistry, 3078 Michigan State University, 578 South Shaw Lane, East Lansing, Michigan 48824, United States

## Abstract

An acid-catalyzed [1,2]-carbon-to-carbon silyl migration is reported for the synthesis of α-silyl aldehydes from α-hydroxy allyl silanes, which can serve as valuable precursors for various organic reactions. During the synthesis of α-hydroxy allyl silanes, an unexpected aldehyde peak in NMR suggested an in situ rearrangement, later confirmed to be acid-driven. Expanding the substrate scope revealed that an alkyl substituent at the β-position was crucial for migration. Mechanistic studies indicated a stepwise process via a carbocation intermediate, as confirmed by stereochemical erosion in enantiomerically enriched substrates. The tertiary carbocation is further stabilized by the β-silicon effect. The advantages of this method include mild reaction conditions, clean conversion, and often no need for purification.

## Graphical abstract

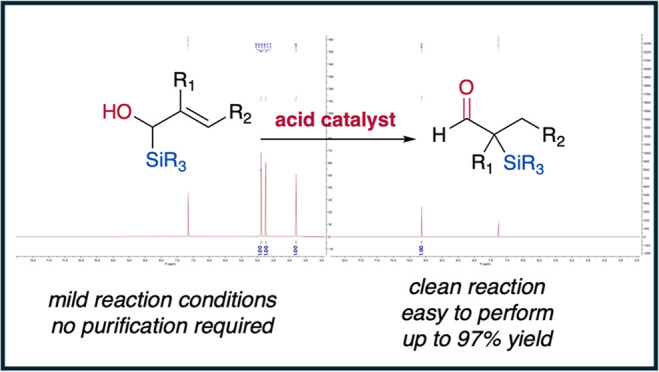

## Introduction

Organosilanes are widely employed in organic synthesis.^1–5^ Aldehydes with a silyl group at the α-position are good precursors for branched propargyl and allyl silanes,^6^ vinyl silyl ethers and aldol reactions (Figure 1).^7^ α-Silyl aldehydes have also been utilized as precursors for the enantioselective synthesis of α-alkenyl glycine methyl esters, α-alkenyl glycine,^8^ and asymmetric [2 + 2] cycloaddition of allenyl imide and mono- or disubstituted alkenes.^9^ Recently, in the total synthesis of (−)-maximiscin, 2-(trimethylsilyl)­acetaldehyde was converted to an oxime at gram scale (Figure 1).^10^

**Figure 1:**
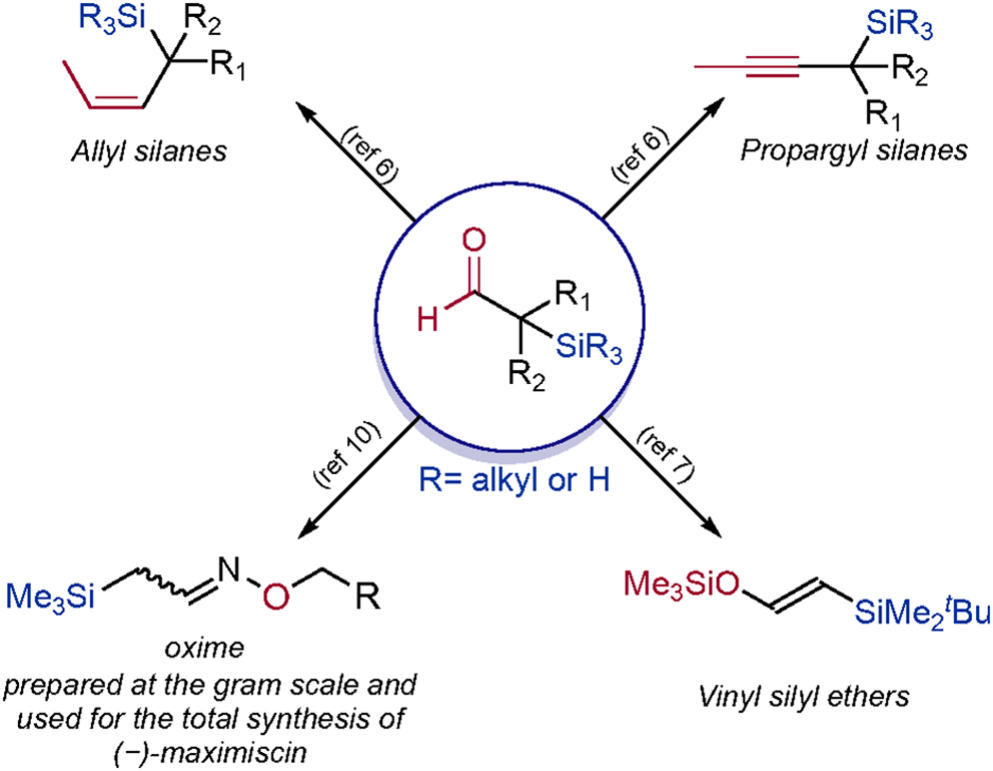
Reported application of α-silyl aldehydes or ketones in organic synthesis.

While several strategies have been developed for the synthesis of α-silyl ketones, analogous methods for α-silyl aldehydes remain significantly more limited.^11–13^ Among earliest examples, opening α,β-epoxysilanes with Grignard reagents generated α-silyl aldehydes that were in situ trapped by the same Grignard reagent, resulting in the formation of β-hydroxysilanes.^14^ The methods used to make the α-silyl aldehydes required for the applications in Figure 1 draw from two literature reports. One demonstrated that by the careful two-phase hydrolysis of silyl imines two α-TBS substituted aldehydes could be isolated (Scheme 1A).^15^ In the second report, α-TBS substituted acetaldehyde was made in 60% yield by a lithiation-silylation-hydrolysis sequence (Scheme 1B).^7^

**Scheme 1:**
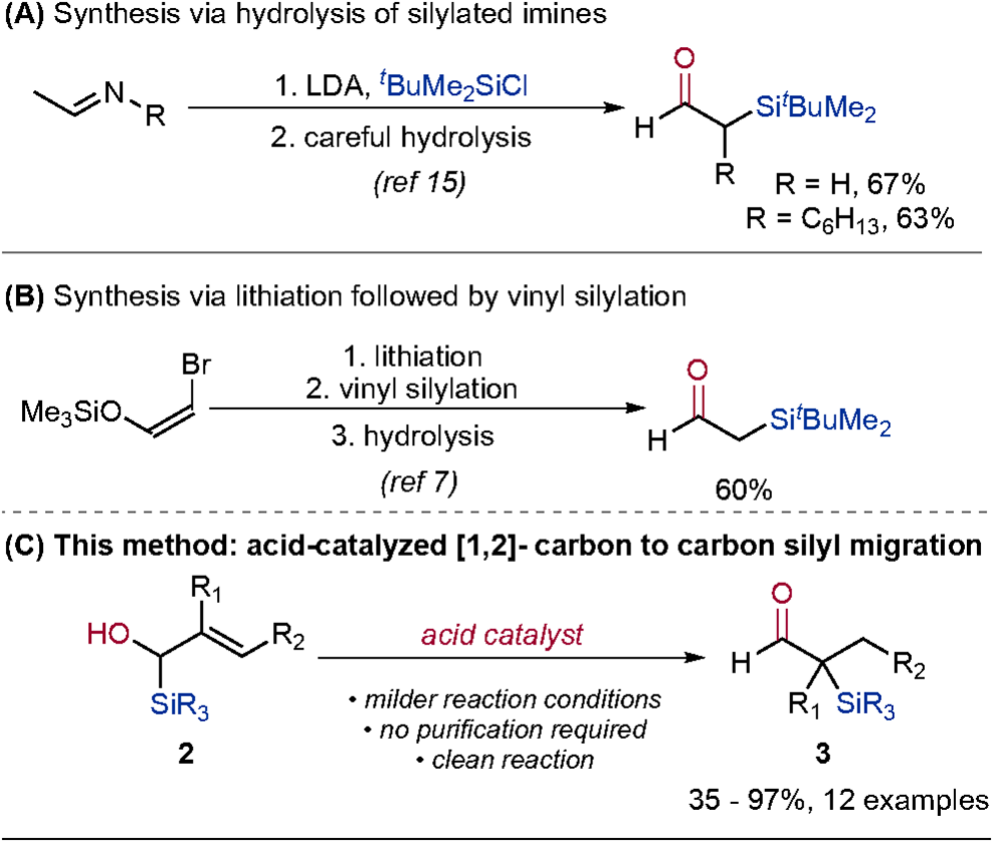
Reported Methods for Synthesizing α-Silyl Aldehydes

## Results and Discussion

Owing to these limited examples, we took note when during the preparation of silanol **2a** (Scheme 2A) a peak at approximately 9.62 ppm appeared in the ^1^H NMR of the crude reaction mixture. Though initially thought to be a minor impurity, the peak persisted in the same ratio (∼1.0:0.2) before and after column chromatography (Figure 2B). As it was unlikely for an aldehyde impurity to coelute with **2a**, we considered the possibility that the aldehyde was not formed during the reaction, but rather in the NMR tube. To investigate further, the sample in the NMR tube was left overnight (∼12 h) and reanalyzed the following day. Indeed, the aldehyde peak had only increased in intensity, signals corresponding to compound **2a** were no longer visible (Figure 2C). The aldehyde was subsequently identified as 2-methyl-2- (triethylsilyl)­propanal (**3a**), which we hypothesized forms via an irreversible [1,2]-carbon-to-carbon silyl migration (Scheme 2B).

**Figure 2:**
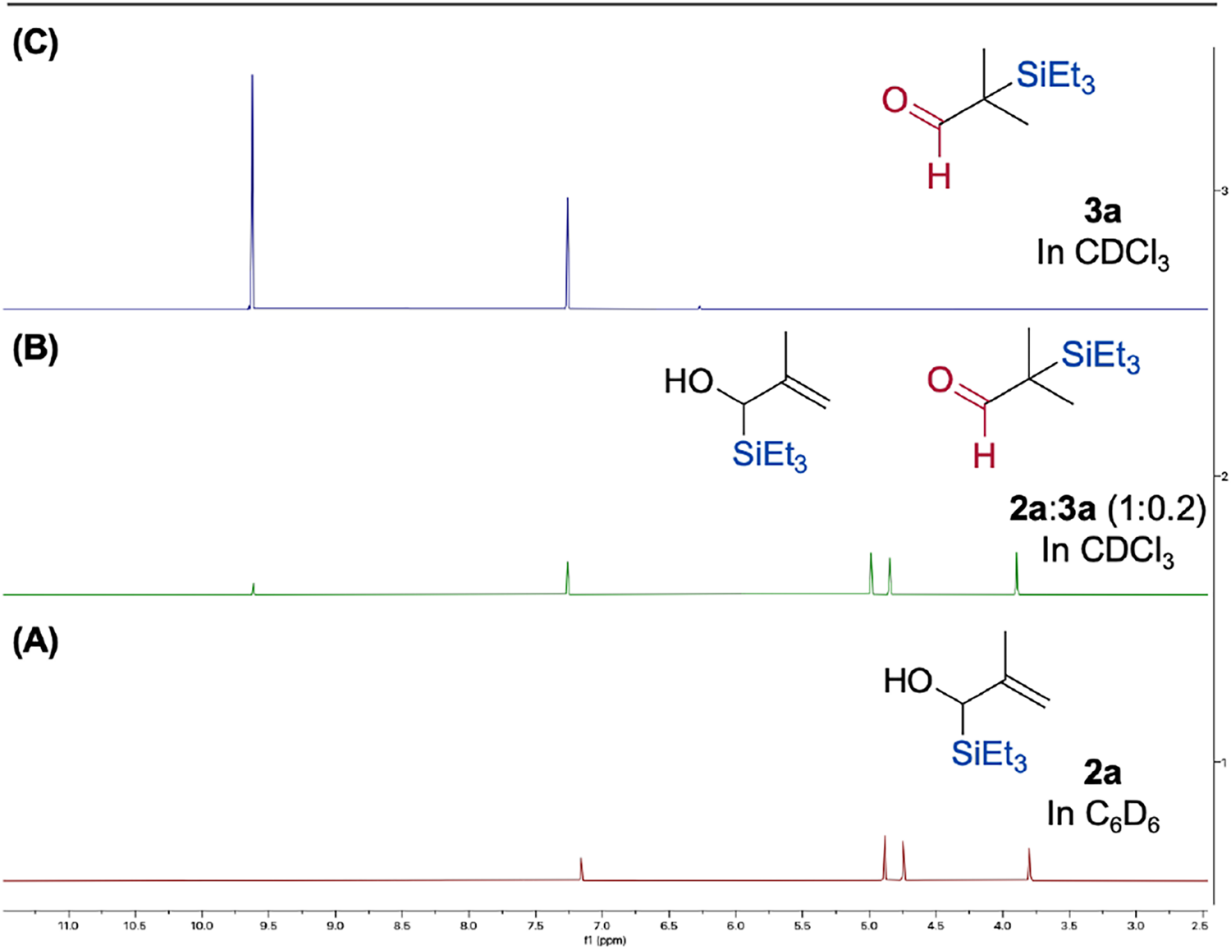
^1^H NMR data for reaction progress in CDCl_3_ (SiEt_3_ protons not shown.) (A). ^1^H NMR data of compound 2a in C_6_D_6_. (B). After 10 min, compound **2a**:**3a** (1:0.2) (C). After 12 h, only compound **3a** is present.

**Scheme 2:**
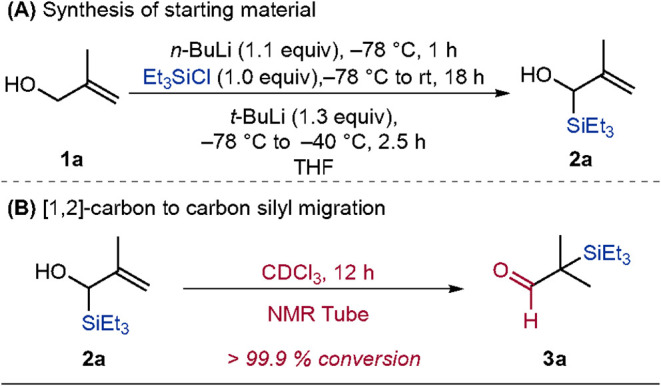
(A) Synthesis of 2-Methyl-1-(triethylsilyl)­prop-2-en-1-ol (**2a**); (B) [1,2]-Carbon to Carbon Silyl Migration of **2a**

Whereas carbon-to-oxygen (Brook rearrangement) or oxygen-to-carbon (retro-Brook rearrangement) silyl shifts are common,^16–21^ carbon-to-carbon silyl migrations are rare, especially [1,2] carbon-to-carbon silyl migrations. Among the reported examples, palladium(0)-catalyzed rearrangement of silicon-substituted vinyl oxiranes have provided three α-silyl β,γ-unsaturated aldehydes in yields ranging from 35–97% (Scheme 3A).^22,23^ In these cases, the presence of a vinyl substituents was said to be required.^23^ The analogous phenyl substituted oxirane was later shown to rearrange in the presence of H_2_SO_4_ (Scheme 3B).^24^ The α-silyl aldehyde prepared in this example was then used in subsequent Peterson olefinations.

**Scheme 3:**
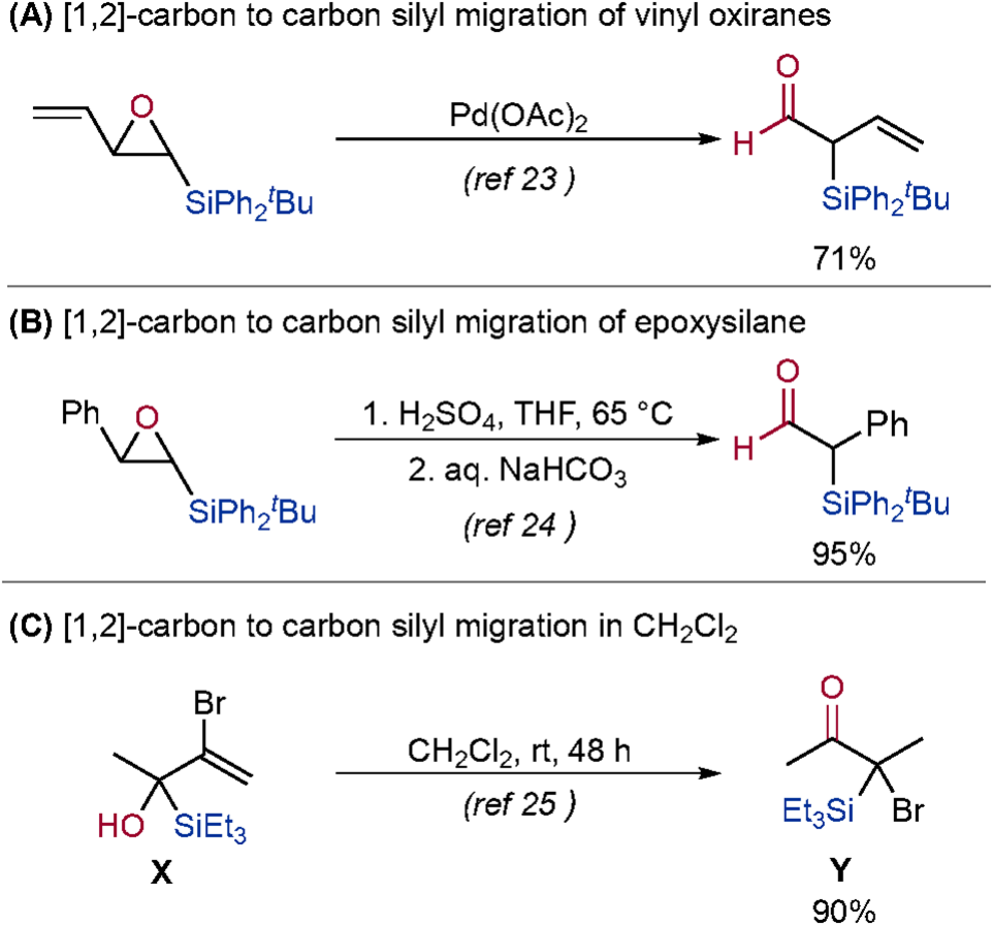
Reported [1,2]-Carbon to Carbon Silyl Migration

The only report of an isolated silyl substituted allylic alcohol undergoing a [1,2] carbon-to-carbon silyl migrations was the intriguing work of Novikov and Sampson, who found that a solution of **X** in CH_2_Cl_2_ slowly converted to “unstable” ketone **Y** (Scheme 3C).^25^ Those authors further noted that “*This unusual rearrangement reaction was not appreciably accelerated by the presence of acids (HCl gas, TsOH), and was completely inhibited by weak bases (pyridine, Et*_*3*_ *N) and even THF*. *We are not aware of a precedent for this rearrangement process*.”^25^ Seeking to reconcile our formation of **3a** and this prior art, compound **2a** was dissolved in deuterated benzene and ^1^H NMR spectra were acquired over time. Even after a week, compound **2a** was unchanged as judged by ^1^H NMR (Figure 2A).

These data clarified that the originally observed [1,2]-carbon-to-carbon silyl migration leading to **3a** had occurred after **2a** had been dissolved in CDCl_3_. It is well-known that deuterated chloroform can become acidic during storage.^26^ Thus, despite prior claims that the aforementioned rearrangement of **A** was not accelerated by acid, we tested to see if **3a** would form in a solution of CDCl_3_ that had been stored over K_2_CO_3_. In such a solution, **3a** was not immediately observed by ^1^H NMR, but the rearrangement of compound **2a** to **3a** occurred gradually over 48 h. Notably, even when using a freshly opened bottle of CDCl_3_, the aldehyde formed over the course of a day.

For practical reasons, we moved to nondeuterated solvents for follow up studies. Not surprisingly, after 1 h a room temperature solution of **2a** (0.2 mmol, 37 mg) in CHCl_3_ (0.5 mL) gave a trace aldehyde peak in the ^1^H NMR spectrum (Table 1, entry 1), which grew over 12 h, ultimately affording **3a** in 95% isolated yield (Table 1, entry 2). With CHCl_3_ stored over K_2_CO_3_, only trace **3a** was detected after 48 h. Still the rearrangement continued to proceed, reaching completion in ∼120 h (Table 1, entries 3 and 4). To further confirm that the reaction is acid-promoted, 0.001 M HCl in chloroform (stored over K_2_CO_3_) was prepared. When a sample of **2a** was dissolved in freshly prepared 0.001 M HCl/CHCl_3_ (2.5 mol %), aldehyde **3a** was formed within one hour (Table 1, entry 5). The reaction proceeded with high purity. Evaporation of the reaction mixture afforded pure **3a** in 98% yield with no further purification required. This experiment provided additional evidence that the reaction was acid-catalyzed, as the introduction of a stronger acid significantly accelerated the reaction. Compound **2a** was also exposed to solutions of 0.001 M HCl in dry toluene and benzene. As shown in Table 1, entries 6 and 7, rearrangement proceeded in these solvent mixtures, but with lower conversions (50% and 37%, respectively). Finally, increasing the molarity of the HCl saw complete loss of **2a**, but various side products accompanied the formation of **3a** (Table 1, entries 8 and 9). Lewis acids were also explored. Using BF_3_·Et_2_O generated a complex mixture, but AlCl_3_ enabled 70% conversion of the starting material to the rearranged product (Table 1, entries 10 and 11). Replacing HCl with silica gel resulted in trace product after 1 h; however, complete conversion was observed after 12 h, though the reaction was not clean (Table 1, entry 12). Lewis base HMPA^27^ had no effect on the reaction (Table 1, entries 13 and 14). Finally, a biphasic reaction with 10% aqueous HCl in D_2_O and CHCl_3_ afforded compound **3a** in 58% yield with complete monodeuteration (CH_2_D) of one of the geminal dimethyls. Therefore, 0.001 M HCl in chloroform was deemed the optimal conditions for this transformation, enabling clean conversion of **2a** to **3a** within one hour at room temperature (Table 1, entry 5).

**Table 1:**
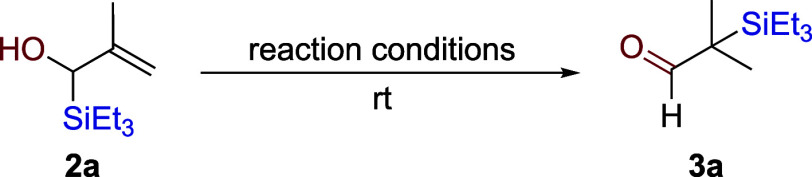
Optimization of Reaction Conditions^a^

entry	acid (base) catalyst^b^	solvent	time (h)	yield (%)^c^ (Observations)^d^
1^e^		CHCl_3_	1	trace (the aldehyde peak shows up)
2^e^		CHCl_3_	12	95 (>99.9% conversion)
3		CHCl_3_ (over K_2_CO_3_)	48	trace (the aldehyde peak shows up)
4		CHCl_3_ (over K_2_CO_3_)	120	80 (>99.9% conversion)
*5*	*0.001 M HCl*	*CHCl_3_ (over K_2_CO_3_)*	*1*	*98 (>99.9% conversion)*
6	0.001 M HCl	Dry toluene	1	(50% conversion)
7	0.001 M HCl	Dry benzene	1	(37% conversion)
8^f^	0.01 M HCl	CHCl_3_ (over K_2_CO_3_)	1	(>99.9% conversion^g^)
9^f^	0.1 M HCl	CHCl_3_ (over K_2_CO_3_)	1	(>99.9% conversion^g^)
10^h^	BF_3_·Et_2_O	CHCl_3_ (over K_2_CO_3_)	1	complex mixture
11^h^	AlCl_3_	CHCl_3_ (over K_2_CO_3_)	1	(70% conversion^g^)
12^h^	Silica gel	CHCl_3_ (over K_2_CO_3_)	1/(12)	trace product/(>99.9% conversion^g^)
13^i^	HMPA	HMPA	1	no reaction
14^i^	HMPA	CHCl_3_ (over K_2_CO_3_)	1	no reaction
15^j^	10% aq. HCl in D_2_O	CHCl_3_ (over K_2_CO_3_)	1	58% (>99 conversion)

a Conditions: All reactions were performed at 0.2 mmol scale, compound **2a** (0.2 mmol, 37 mg), and 0.5 mL of solvent was added.

b Different molarities of HCl were prepared in the mentioned solvents.

c Isolated yields.

d Based on ^1^H NMR spectra.

e Similar results were obtained when the reaction was performed in CDCl_3_ in place of CHCl_3_.

f Reactions were performed at compound **2b** (0.3 mmol, 43 mg).

g Side products were also observed by ^1^H NMR.

h 0.1 equiv of acid catalyst.

i (1.0 equiv) HMPA.

j (0.5 mL) 10% aqueous HCl in D_2_O and (0.5 mL) CHCl_3_.

We next explored the substrate scope of this [1,2] carbon-to-carbon silyl migration. Alcohols with various silyl (R) substituents (**2a**–**2l**) were synthesized from a readily available corresponding alcohol and subjected to 0.001 M HCl (Scheme 4). Starting alcohols with alkyl groups of various chain lengths and R_1_ = CH_3_ and R_2_ = H held constant, rearranged to the corresponding aldehydes (**3a–3d**) in 35–98% yield after reaction times ranging from 10 to 60 min. Synthesis of the TIPS substituted alcohol (**2e**) was more challenging than that of the *n*-propyl derivative, particularly at the retro-brook step. Nonetheless, rearrangement of compounds **2e** afforded **3e** in 66% yield. TBS silylated alcohol **2f** was efficiently converted to aldehyde **3f**. Additionally, alcohols where R = phenyl or methyl were well tolerated, affording the corresponding alkanals **3g** and **3h** in 68% and 66% yields, respectively. Expanding the scope where R_1_ was varied (keeping R_2_ = H) was challenged by limited commercial availability of the corresponding alcohols. Thus, we modified a literature procedure to synthesize **2i** and **2j** in two steps (see Supporting Information for details). When subjected to the acidic conditions, both substrates were fully consumed to give corresponding aldehydes **3i** and **3j** in 69% and 40% yields, respectively. Later when R_2_ was varied (keeping R_1_ = CH_3_) **3k** and **3l** were obtained in 62% and 69% yields, respectively. Attempts to synthesize silylated starting material with having R_1_= phenyl led to an S_N_2-like reaction between the O-silylated 2-phenylprop-2-en-1-ol and butyl lithium.

**Scheme 4:**
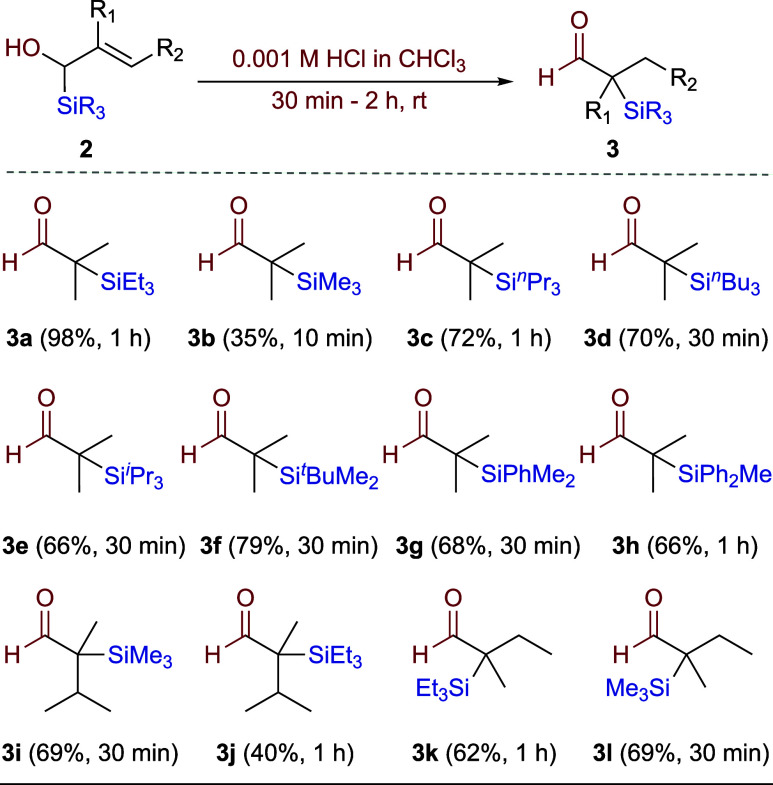
Substrate Scope of [1,2]-Carbon to Carbon Silyl Migration

The robustness of this reaction was further demonstrated by performing it on a gram scale. 2-Methyl-1-(triethylsilyl)­prop-2-en-1-ol (**2a**, 5.4 mmol, 1g) was treated with 0.001 M HCl in CHCl_3_ (9 mL), affording **3a** in 91% yield (920 mg, 4.93 mmol) after passage through a silica plug (Scheme 5A). Surprisingly, when R_1_ = H and R = CH_3_, the silylated alcohol **2x** did not rearrange to give **3x**. Similarly, no silyl migration was observed for **2y** after several days in CDCl_3_ or under standard condtions (Scheme 5B). These experiments emphasized the importance of having an alkyl substituent at the olefin carbon β to the silicon for the reaction to proceed (Scheme 5B). As rearrangement with tertiary allylic alcohols would afford ketones, we explored the reactivity of compounds **2m** and **2n**, which had the additional feature of being cyclic. To prepare these substrates, freshly prepared PhMe_2_SiLi was added to the corresponding enones under cold conditions, which afforded **2m** and **2n** (see Experimental Section for details). Upon subjecting **2m** to the standard reaction conditions, no rearrangement to ketone **3m** was observed even after 12 h. In the case of **2n** (R_1_ = CH_3_), only trace quantities of ketone **3n** were detected after 12 h (Scheme 5C). Given these results, challenges associated with the preparation of other tertiary allylic alcohol substrates, and the prior work on α-silyl ketones by others,^11–13^ we decided to maintain our focus on α-silyl aldehyde formation. No other tertiary allylic alcohols were examined.

**Scheme 5:**
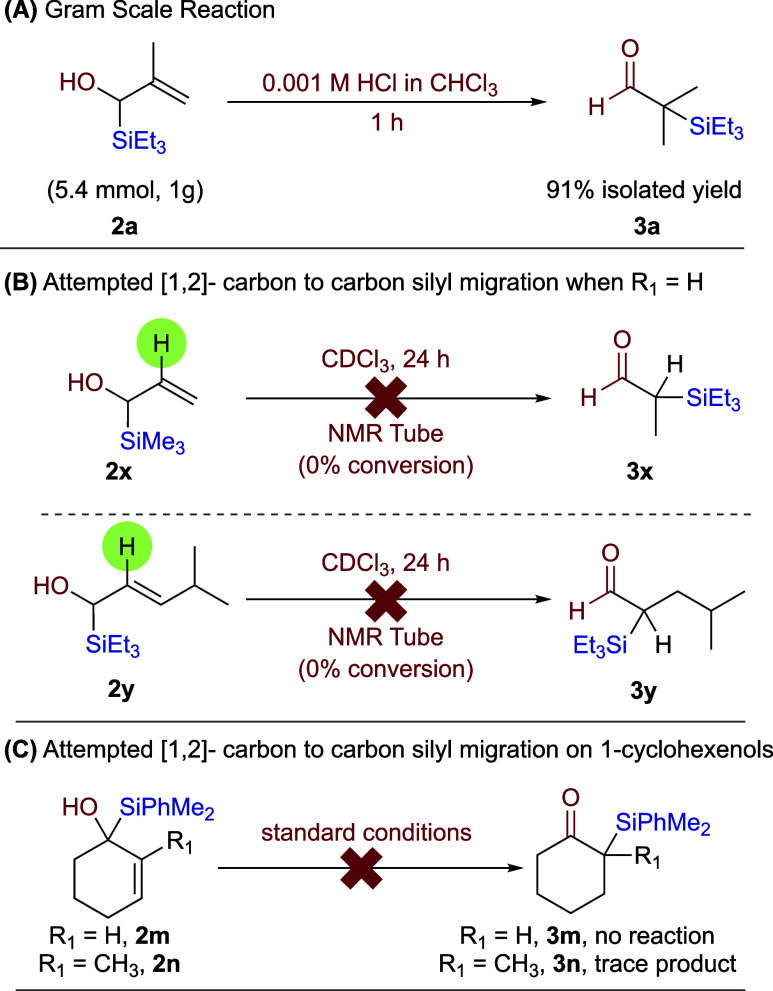
(A) Gram-Scale Synthesis; (B, C) Attempted [1,2]-Carbon to Carbon Silyl Migration When R_1_ = H and on 1-Cyclohexanol Substrates

Our next objective was to determine if chirality transferred upon rearrangement, thus suggesting a concerted mechanism. Specifically, we asked if an enantiomerically enriched **2** would lead to an optically active **3** and if so, would the result be an inversion or retention of stereochemistry. We targeted 3-methyl-2-methylene-1-(triethylsilyl)­butan-1-ol (±)-**2j** as the starting material for these investigations. Swern oxidation of (±)-**2j**, followed by asymmetric reduction of the resulting acyl silane (**2z**) using the Corey–Bakshi–Shibata (CBS) catalyst^28^ afforded (+)-**2j** (Scheme 6). To rigorously determine the extent of any chirality transfer, we analyzed (±)-**3j** by chiral HPLC and chiral SFC/MS. Unfortunately, neither method resolved the enantiomeric partners. Furthermore, attempts to derivatize **3j** resulted in loss of the silyl group. As determining the enantiomeric excess of **3j** would only be necessary if some chirality transfer occurred, we turned to optical rotation for evidence that the rearrangement of (+)-**2j** gave nonracemic **3j**. This was not the case as the [α]_D_ of **3j** was approximately zero (see Experimental Section).

**Scheme 6:**
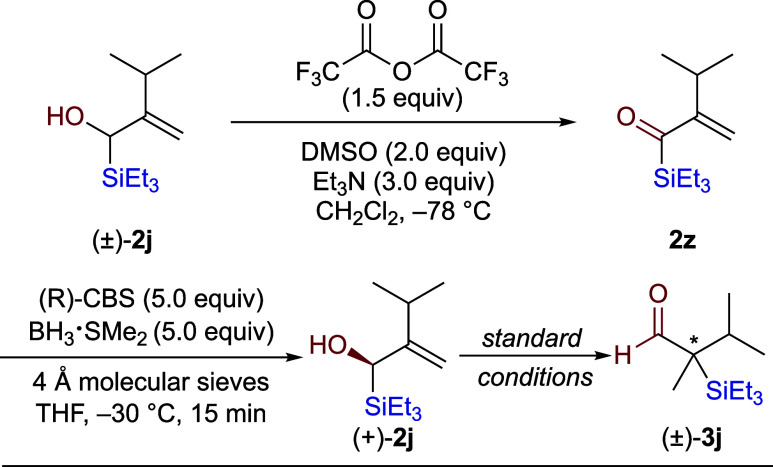
Chirality Transfer Experiment

The necessities of an acid catalyst and a β-alkyl group adjacent to the silyl group, the deuterium incorporation into one of the gem dimethyls of **3a** when the rearrangement was run with in situ generated DCl (Table 1, entry 15), and the apparent stereochemical erosion during rearrangement of (+)-**2j**, are consistent with a stepwise mechanism involving a carbocation intermediate. Thus, we propose that this transformation is acid-catalyzed. Upon protonation, the olefin forms a tertiary carbocation,^29^ which is also stabilized by the β-silicon effect.^30,31^ This intermediate undergoes a subsequent [1,2]-silyl migration, which is facilitated by carbonyl formation, ultimately yielding the α-silyl aldehydes (Scheme 7).

**Scheme 7:**
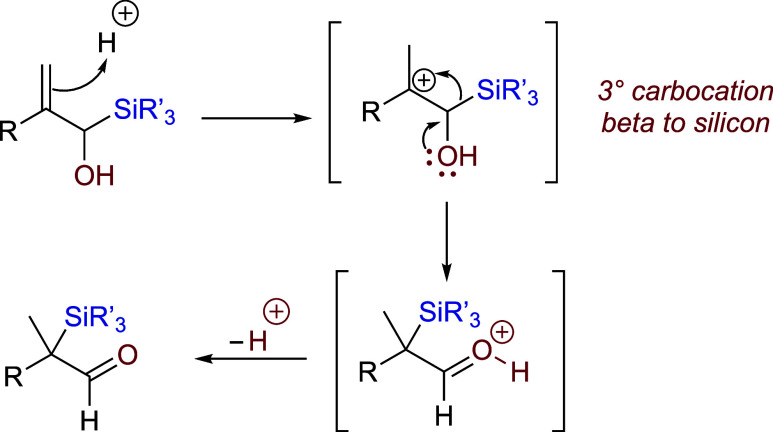
Proposed Reaction Mechanism

## Conclusions

Overall, we have discovered an irreversible acid-catalyzed [1,2]-carbon-to-carbon silyl migration leading to α-silylated alkanals. This migration proceeds even when the substituents on silicon are modified but requires an alkyl group at the olefin carbon β to silicon. Preliminary studies have also supported a stepwise mechanism. Further extension of substrate scope and mechanistic investigation is currently going on in our laboratory.

## Experimental Section

### General Information

All commercially available chemicals were used as received unless otherwise indicated. All reactions were run under a positive atmosphere of nitrogen in oven-dried or flame-dried round-bottom flasks capped with rubber septa. Column chromatography was run on 230–400 mesh silica gel. Visualization was accomplished with UV light and/or potassium permanganate stain by heating with a heat gun. Tetrahydrofuran and diethyl ether were distilled from sodium-benzophenone ketyl; dichloromethane, benzene, triethylamine, cyclohexane, toluene and trimethylsilyl chloride were distilled from calcium hydride. Hexanes, *n*-butyllithium (2.5 M in hexanes) and *t*-butyllithium (1.7 M in pentanes/1.9 M in pentanes) were purchased and used as received. Warning: Organolithium especially *t*-butyllithium is extremely pyrophoric and ignites spontaneously on contact with air or moisture. All manipulations must be conducted under inert atmosphere (dry Ar or N_2_) using Schlenk techniques, flame-dried glassware, and anhydrous solvents. Transfers were performed by gastight syringe or cannula, and additions carried out at −78 °C. Do not use water on organolithium fires; use Class D extinguishers or dry sand. Appropriate PPE must be worn at all times and all operations conducted in a fume hood.^1^H NMR and ^13^C NMR spectra were collected on a 500 MHz instrument using C_6_D_6_ (referenced at 7.16 and 128.06 ppm, respectively) and CDCl_3_ as the solvent (referenced at 7.26 and 77.16 ppm, respectively). High-resolution mass spectra (HRMS) were obtained at the Michigan State University Mass Spectrometry Service Center using a LECO Pegasus GC-HRT on a quadrupole time-of-flight (Q-TOF) instrument. CHN elemental analyses were conducted on a PerkinElmer 2400 Series II CHNS/O analyzer. IR and Polarimeter data were taken on a Jasco FT/IR 6600 and Jasco P-2000, respectively.

#### General Procedure A for the Synthesis of Alcohols (**1b**–**1c**)

Following a modified procedure,^32^ for a 300 mmol reaction, a 3-neck 500 mL round-bottomed flask fitted with a magnetic stir bar was weighed with magnesium powder (3.3 equiv), 2 crystals of iodine and placed over an oil bath. This was followed by the addition of 150 mL dry THF. After this, the side necks of the flask were sealed with two rubber septa and a reflux condenser attached to the middle neck and then purged with nitrogen. In a separate 250 mL round-bottomed flask, a solution of the respective alkyl halide (3.0 equiv) was prepared in 100 mL dry THF. Using a cannula, a 250 mL flask was connected to the 3-neck 500 mL flask. The alkyl halide solution was transferred to the 3-neck flask via a cannula in a dropwise manner over 30 min. The temperature of the oil bath was monitored, and it was found to be about 40 °C after the complete addition of the alkyl halide solution. The cannula was removed, and the temperature of the oil bath was raised to 75 °C to form the Grignard reagent over 1.5 h. The heat was then turned off, and the solution was allowed to come to room temperature after the oil bath had been removed. In a separate 250 mL round-bottomed flask, copper iodide (0.15 equiv) was weighed, followed by the addition of propargylic alcohol (1.0 equiv) as a solution in 100 mL dry THF. This mixture was stirred at room temperature for 30 min. The Grignard reagent in the 3-neck 500 mL flask was cooled down further to 0 °C by placing an ice bath underneath the flask. A cannula was used to connect both the flask and the mixture containing propargylic alcohol and CuI in dry THF, which was transferred to the Grignard reagent in a dropwise manner over 45 min by maintaining the temperature of the ice bath at 0 °C. After the complete addition reaction, the mixture was stirred at room temperature for 24 h. The mixture was then cooled to 0 °C and quenched by the slow addition of 80 mL saturated aqueous ammonium chloride solution. The mixture was transferred into a 1000 mL separating funnel and diluted with 100 mL of diethyl ether. The aqueous layer was extracted with diethyl ether (50 mL × 3). The combined organic layers were washed with saturated aqueous ammonium chloride solution (50 mL), water (50 mL) and brine (50 mL), respectively and then dried over anhydrous magnesium sulfate. This was followed by filtration and concentration under reduced pressure to afford the desired alcohol, which was purified via column chromatography (Et_2_O/hexanes).

#### General Procedure B for the Synthesis of α-Hydroxy Allyl Silanes (**2a**–**2l**)

Following a literature procedure,^33^ to a 250 mL flame-dried round-bottom flask, a solution of corresponding allyl alcohol (1.0 equiv) in THF (150 mL) was added and purged with nitrogen. This solution was cooled to −78 °C. *n*-BuLi (2.5 M in hexanes, 1.1 equiv) was added dropwise, and the mixture was stirred at −78 °C for 1 h. The corresponding chlorosilane (1.0 equiv) was then added dropwise from a syringe, and the resulting mixture was stirred at −78 °C for a given amount of time (see individual compounds procedure below) to form a white suspension. This was followed by the addition of *tert*-BuLi (1.7 M in pentane, 1.3 equiv) dropwise via cannula or syringe, and the reaction was stirred for an additional time. Then, the reaction was kept at the indicated temperature (see individual compounds procedure below). The reaction was quenched by the addition of DI water and diluted with Et_2_O (50 mL). Then, the mixture was allowed to warm up slowly to room temperature. After the layers were separated using a separatory funnel 500 mL, the aqueous phase was washed with Et_2_O (3 × 50 mL). Then all the organic phases were washed with H_2_O (50 mL) and brine (50 mL), respectively. The organic layer was dried over anhydrous MgSO_4_. This was followed by filtration and the filtrate was concentrated under reduced pressure, which afforded α-hydroxy allyl silanes after column chromatography (Et_2_O/hexanes or EtOAc hexanes).

#### General Procedure C for the Synthesis of α-Silyl Alkanals (**3a**–**3l**)

To a 4 mL vial with a magnetic stir bar, α-hydroxy allyl silane (1.0 equiv) was added. This was followed by adding a freshly prepared solution of 0.001 M HCl in chloroform (5 mL). Chloroform used for the preparation of the reagent was stored over potassium carbonate. The flask was sealed with a rubber septum and purged with nitrogen. Depending on the substrate, the reaction mixture was stirred at room temperature for 30 min to 2 h. The resulting mixture was concentrated under reduced pressure and purified by silica plug (if necessary) to afford **3**. In Scheme 4, different times are indicated for each substrate. Reactions were monitored via ^1^H NMR, and at the specified time points, no peaks corresponding to the starting materials were observed in the spectra.

#### General Procedure D for the Synthesis of α-Hydroxy Allyl Cylcohexenyl Silanes (**2m** and **2n**)

Following a modified procedure,^34^ Lithium metal (224 mg, 32.33 mmol, 5 equiv) was cut into small pieces, washed with hexanes, and dried under vacuum in an oven-dried two-necked round-bottom flask. Under a nitrogen atmosphere, dry THF (12 mL) was added via syringe, followed by dropwise addition of chlorodimethylphenylsilane (1.3 mL, 7.35 mmol, 1 equiv) over 5 min at room temperature. The reaction mixture was stirred at room temperature for 16 h, affording a dark brown solution of PhMe_2_SiLi in THF, which was used directly in subsequent reactions without further purification.

To a stirred solution of the corresponding enone (3.0 mmol, 1 equiv) in dry THF at −78 °C under a nitrogen atmosphere was added the freshly prepared PhMe_2_SiLi solution (approximately 1.2 equiv, 0.5 M solution) dropwise via syringe. The resulting mixture was maintained at −78 °C and reaction progress was monitored by TLC. Upon complete consumption of the starting material, the reaction was quenched with saturated aqueous NH_4_Cl (30 mL) and extracted with EtOAc (2 × 50 mL). The combined organic layers were washed with brine (40 mL) and dried over MgSO_4_. The solvent was removed under reduced pressure and the crude residue was purified by column chromatography to afford the desired compounds.

#### Synthesis of 3-Methyl-2-methylenebutan-1-ol (**1b**)

Following general procedure A, isopropyl bromide (28 mL, 300.0 mmol, 3.0 equiv) was added to the solution of magnesium powder (8.0 g, 330.0 mmol, 3.3 equiv) in dry THF (150 mL) and refluxed at 75 °C for one and a half hours to synthesize the Grignard reagent. Grignard reagent was cooled down to 0 °C and was transferred using a cannula to a 500 mL round-bottom flask containing CuI (2.86 g, 15.0 mmol, 0.15 equiv) and propargylic alcohol (5.9 mL, 100 mmol, 1.0 equiv) in dry THF at 0 °C. This was followed by workup, concentration and column chromatography (30% Et_2_O in hexanes), which afforded 6.41 g of compound **1b** and **1c** as a 20:1 yellow oil mixture. (64% yield, 64.0 mmol).

##### Spectroscopic Data for Compound **1b**

^1^H NMR (500 MHz, CDCl_3_) δ = 4.99 (q, *J* = 1.6 Hz, 1H, vinyl H), 4.87 (h, *J* = 1.2 Hz, 1H, vinyl H), 4.13–4.08 (m, 2H, CH_2_), 2.31 (hept, *J* = 6.8 Hz, 1H, isopropyl CH), 1.94 (s, 1H, broad, OH), 1.05 (d, *J* = 6.9 Hz, 6H, isopropyl CH_3_). ^13^C­{^1^H} NMR (126 MHz, CDCl_3_) δ = 155.3, 107.0, 65.0, 31.1, 21.9 (2C). Spectroscopic data for compounds **1b** and **1c** (minor product) are similar to those reported in the literature.^32,35^

#### Synthesis of (*E*)-2-Methylbut-2-en-1-ol (**1d**)

Following a modified reported procedure,^36^ NaBH_4_ (2.96 g, 78.2.0 mmol, 2.0 equiv) was added to the solution of commercially purchased (*E*)-2-methylbut-2-enal (3.8 mL, 39.1 mmol, 1.0 equiv) in methanol (35 mL) at 0 °C. After 4 h, the reaction was quenched by adding deionized water and extracted with EtOAc instead of CH_2_Cl_2_. This was followed by concentration on rotavapor, which afforded 2.26 g of compound **1d** (60% yield, 23.5 mmol) as yellow oil which was used in the next step without further purification.

##### Spectroscopic Data for Compound **1d**

^1^H NMR (500 MHz, CDCl_3_) δ = 5.56–5.42 (m, 1H, vinyl H), 3.98 (d, *J* = 2.7 Hz, 2H, CH_2_), 1.77 (s, 1H, broad, OH), 1.65 (s, 3H, vinyl CH_3_), 1.61 (dt, *J* = 6.7, 1.2 Hz, 3H, vinyl CH_3_). ^13^C­{^1^H} NMR (126 MHz, CDCl_3_) δ = 135.6, 120.7, 69.1, 13.5, 13.2. Spectroscopic data are similar to those previously reported.^36^

#### Synthesis of 2-Methyl-1-(triethylsilyl)­prop-2-en-1-ol (**2a**)

Following general procedure B, 2-methyl allyl alcohol (1.26 mL, 15.0 mmol, 1.0 equiv) in THF (50 mL) was cooled to −78 °C. To this solution, *n*-BuLi (7.2 mL, 2.5 M in hexane, 18.0 mmol, 1.2 equiv) was added dropwise and stirred at −78 °C for 1 h. After 1 h, triethyl silyl chloride (2.52 mL, 15.0 mmol, 1.0 equiv) was dropwise added, and the reaction mixture was stirred at −78 °C to room temperature for 18 h. After further cooling down to −78 °C, *t*-BuLi (10.5 mL, 1.7 M in pentane, 18.0 mmol, 1.2 equiv) was added dropwise and the reaction mixture was stirred at −78 °C to −40 °C for 3.5 h. This was followed by workup, concentration and column chromatography, which afforded 1.4 g of compound **2a** as a colorless liquid (50% yield, 7.5 mmol). *R*_f_ = 0.2. (5% Et_2_O in hexanes).

##### Spectroscopic Data for Compound **2a**

^1^H NMR (500 MHz, C_6_D_6_) δ = 4.88 (dq, *J* = 2.2, 1.0 Hz, 1H, vinyl H), 4.77–4.72 (m, 1H, vinyl H), 3.80 (s, 1H, CH), 1.65–1.58 (m, 3H, vinyl CH_3_), 1.03 (t, *J* = 8.0 Hz, 9H, triethyl CH_3_), 0.96 (s, 1H, OH), 0.65 (tq, *J* = 14.9, 7.7 Hz, 6H, triethyl CH_2_). ^13^C­{^1^H} NMR (126 MHz, C_6_D_6_) δ = 149.1, 106.7, 69.8, 20.7, 7.8, 2.5. ^29^Si NMR (99 MHz, C_6_D_6_) δ = 5.1. IR (neat, cm^–1^): 3440 (broad). HRMS (EI): *m*/*z* calcd for C_10_H_22_OSi [M]^+^: 186.1439; found: 186.1432.

#### Synthesis of 2-Methyl-1-(trimethylsilyl)­prop-2-en-1-ol (**2b**)

Following general procedure B, 2-methyl allyl alcohol (0.84 mL, 10.0 mmol, 1.0 equiv) in THF (50 mL) was cooled to −78 °C. To this solution, *n*-BuLi (4.4 mL, 2.5 M in hexane, 11.0 mmol, 1.1 equiv) was added dropwise and stirred at −78 °C for 1 h. After 1 h, trimethylsilyl chloride (1.26 mL, 10.0 mmol, 1.0 equiv) was dropwise added, and the reaction mixture was stirred at −78 °C for 1.5 h, then *t*-BuLi (7.6 mL, 1.7 M in pentane, 13.0 mmol, 1.3 equiv) was added dropwise, and the reaction mixture was stirred at −78 °C to −40 °C for 3.5 h. This was followed by workup, concentration and column chromatography, which afforded 560.0 mg of compound **2b** as a colorless liquid (41% yield, 3.88 mmol). *R*_f_ = 0.2 (15% Et_2_O in hexanes).

##### Spectroscopic Data for Compound **2b**

^1^H NMR (500 MHz, C_6_D_6_) δ = 4.85 (dq, *J* = 1.7, 0.9 Hz, 1H, vinyl H), 4.74 (q, *J* = 1.5 Hz, 1H, vinyl H), 3.63 (s, 1H, CH), 1.59–1.56 (m, 3H, vinyl CH_3_), 1.32 (s, 1H,broad, OH), 0.08 (s, 9H, Si­(CH_3_)_3_). ^13^C­{^1^H} NMR (126 MHz C_6_D_6_) δ = 148.6, 106.6, 71.4, 20.8, −3.3. ^29^Si NMR (99 MHz, C_6_D_6_) δ = 1.3. Spectroscopic data are consistent with those reported in the literature.^33^

#### Synthesis of 2-Methyl-1-(tripropylsilyl)­prop-2-en-1-ol (**2c**)

Following general procedure B, 2-methyl allyl alcohol (1.7 mL, 20.0 mmol, 1.0 equiv) in THF (50 mL) was cooled to −78 °C. To this solution, *n*-BuLi (9.6 mL, 2.5 M in hexane, 24.0 mmol, 1.2 equiv) was added dropwise and stirred at −78 °C for 1 h. After 1 h, tri*n*-propyl silyl chloride (4.3 mL, 20.0 mmol, 1.0 equiv) was dropwise added, and the reaction mixture was stirred at −78 °C to room temperature for 18 h. The reaction mixture was further cooled down to −78 °C, and *t*-BuLi (14.0 mL, 1.7 M in pentane, 24.0 mmol, 1.2 equiv) was added dropwise, and the reaction mixture was stirred at −78 °C to −40 °C for 3.5 h. This was followed by workup, concentration and column chromatography, which afforded compound 2.4 g of **2c** as a colorless liquid (53.6% yield, 10.7 mmol). *R*_f_ = 0.2 (10% Et_2_O in hexanes).

##### Spectroscopic Data for Compound **2c**

^1^H NMR (500 MHz, C_6_D_6_) δ = 4.89 (dd, *J* = 1.8, 1.0 Hz, 1H, vinyl H), 4.75 (q, *J* = 1.5 Hz, 1H, vinyl H), 3.80 (s, 1H, CH), 1.63 (s, 3H, vinyl CH_3_), 1.49–1.36 (m, 6H, *n*-Pr CH_3_), 1.06–0.99 (m, 10H, *n*-Pr & OH), 0.72–0.61 (m, 6H, *n*-Pr). ^13^C­{^1^H} NMR (126 MHz, C_6_D_6_) δ = 149.1, 106.8, 70.2, 20.8, 19.0, 17.9, 14.3. ^29^Si NMR (99 MHz, C_6_D_6_) δ = 0.7. IR (neat, cm^–1^): 3449 (broad). HRMS (EI): *m*/*z* calcd for C_13_H_28_OSi [M]^+^: 228.1909; found: 228.1904

#### Synthesis of 2-Methyl-1-(tributylsilyl)­prop-2-en-1-ol (**2d**)

Following general procedure B, 2-methyl allyl alcohol (1.26 mL, 15.0 mmol, 1.0 equiv) in THF (50 mL) was cooled to −78 °C. To this solution, *n*-BuLi (7.2 mL, 2.5 M in hexane, 18.0 mmol, 1.2 equiv) was added dropwise and stirred at −78 °C for 1 h. After 1 h, tributyl silyl chloride (4.0 mL, 15.0 mmol, 1.0 equiv) was dropwise added, and the reaction mixture was stirred at −78 °C to room temperature for 18 h. The reaction mixture was further cooled down to −78 °C, and *t*-BuLi (10.5 mL, 1.7 M in pentane, 18.0 mmol, 1.2 equiv) was added dropwise, and the reaction mixture was stirred at −78 °C to −40 °C for 3.5 h. This was followed by workup, concentration and column chromatography, which afforded 1.69 g of compound **2d** as a colorless liquid (41.6% yield, 6.25 mmol).

##### Spectroscopic Data for Compound **2d**

^1^H NMR (500 MHz, C_6_D_6_) δ = 4.92 (td, *J* = 1.8, 0.9 Hz, 1H, vinyl H), 4.77 (q, *J* = 1.5 Hz, 1H, vinyl H), 3.84 (s, 1H, CH), 1.69–1.62 (m, 3H, vinyl CH_3_), 1.49–1.35 (m, 12H, *n*-Bu), 0.94 (t, *J* = 7.1 Hz, 9H, *n*-Bu), 0.91 (s, 1H, OH), 0.80–0.64 (m, 6H, *n*-Bu). ^13^C­{^1^H} NMR (126 MHz, C_6_D_6_) δ = 149.1, 106.9, 70.2, 27.3, 26.6, 20.9, 14.1, 11.3. ^29^Si NMR (99 MHz, C_6_D_6_) δ = 1.7. IR (neat, cm^–1^): 3442 (broad). Elemental Anal. Calcd for C_16_H_34_OSi: C, 71.04; H, 12.67; O, 5.91; Si, 10.38. Found: C, 70.69; H, 12.51.

#### Synthesis of 2-Methyl-1-(triisopropylsilyl)­prop-2-en-1-ol (**2e**)

Following general procedure B, 2-methyl allyl alcohol (1.69 mL, 20.0 mmol, 1.0 equiv) in THF (50 mL) was cooled to −78 °C. To this solution, *n*-BuLi (9.6 mL, 2.5 M in hexane, 24.0 mmol, 1.2 equiv) was added dropwise and stirred at −78 °C for 1 h. After 1 h, triisopropyl silyl chloride (4.3 mL, 20.0 mmol, 1.0 equiv) was dropwise added, and the reaction mixture was stirred at −78 °C to room temperature for 18 h. The reaction mixture was further cooled down to −78 °C, and *t*-BuLi (14.0 mL, 1.7 M in pentane, 20.0 mmol, 1.2 equiv) was added dropwise, and the reaction mixture was stirred at −78 °C to −40 °C for 3.5 h. This was followed by workup, concentration and column chromatography (10% Et_2_O in hexanes), which afforded 284 mg of compound **2e** as a colorless liquid (6.2% yield, 1.24 mmol).

##### Spectroscopic Data for Compound **2e**

^1^H NMR (500 MHz, C_6_D_6_) δ = 4.95 (dt, *J* = 1.8, 0.9 Hz, 1H, vinyl H), 4.76–4.74 (m, 1H, vinyl H), 3.94 (s, 1H, CH), 1.67 (d, *J* = 1.5 Hz, 3H, vinyl CH_3_), 1.20–1.18 (m, 11H, *i*-Pr), 1.17–1.15 (m, 11H *i*-Pr). ^13^C­{^1^H} NMR (126 MHz, C_6_D_6_) δ = 149.8, 69.3, 21.0, 19.2, 19.1, 11.7. ^29^Si NMR (99 MHz, C_6_D_6_) δ = 13.3. IR (neat, cm^–1^): 3327 (broad). HRMS (EI): *m*/*z* calcd for C_13_H_28_OSi [M]^+^: 228.1909; found: 228.1904.

#### Synthesis of 1-(*tert*-Butyldimethylsilyl)-2-methylprop-2-en-1-ol (**2f**)

Following general procedure B, 2-methyl allyl alcohol (0.84 mL, 10.0 mmol, 1.0 equiv) in THF (50 mL) was cooled to −78 °C. To this solution, *n*-BuLi (4.8 mL, 2.5 M in hexane, 12.0 mmol, 1.2 equiv) was added dropwise and stirred at −78 °C for 1 h. After 1 h, *tert*-butylchlorodimethylsilane (1.5 g, 10.0 mmol, 1.0 equiv) was dropwise added, and the reaction mixture was stirred at −78 °C to room temperature for 18 h. The reaction mixture was further cooled down to −78 °C, and *t*-BuLi (7.0 mL, 1.7 M in pentane, 12.0 mmol, 1.2 equiv) was added dropwise, and the reaction mixture was stirred at −78 °C to −40 °C for 3.5 h. This was followed by workup, concentration and column chromatography, which afforded 375 mg of compound **2f** as a colorless liquid (20% yield, 2.0 mmol). *R*_f_ = 0.3 (2.5% Et_2_O in hexanes).

##### Spectroscopic Data for Compound **2f**

^1^H NMR (500 MHz, C_6_D_6_) δ = 4.84 (ddd, *J* = 2.2, 1.5, 0.8 Hz, 1H, vinyl H), 4.74 (ddd, *J* = 2.2, 1.5, 0.8 Hz, 1H, vinyl H), 3.79 (s, 1H, CH), 1.59 (dd, *J* = 1.3, 0.7 Hz, 3H, vinyl CH_3_), 1.03 (s, 9H, *t*-Bu), 0.83–0.76 (m, 1H, OH), 0.09 (s, 3H, Si­(CH_3_)), −0.04 (s, 3H, Si­(CH_3_)). ^13^C­{^1^H} NMR (126 MHz, C_6_D_6_) δ = 149.3, 107.6, 70.1, 27.2, 20.8, 17.4, −6.2, −8.5. ^29^Si NMR (99 MHz, C_6_D_6_) δ = 6.3. IR (neat, cm^–1^): 3458 (broad). Elemental Anal. Calcd for C_10_H_22_OSi: C, 64.45; H, 11.90. Found: C, 64.09; H, 11.74.

#### Synthesis of 1-(Dimethyl­(phenyl)­silyl)-2-methylprop-2-en-1-ol (**2g**)

Following general procedure B, 2-methyl allyl alcohol (1.69 mL, 20.0 mmol, 1.0 equiv) in THF (100 mL) was cooled to −78 °C. To this solution, *n*-BuLi (9.6 mL, 2.5 M in hexane, 24.0 mmol, 1.2 equiv) was added dropwise and stirred at −78 °C for 1 h. After 1 h, chlorodimethyl­(phenyl)­silane (3.35 mL, 20.0 mmol, 1.0 equiv) was dropwise added, and the reaction mixture was stirred at −78 °C to room temperature for 18 h. The reaction mixture was further cooled down to −78 °C, and *t*-BuLi (14.1 mL, 1.7 M in pentane, 24.0 mmol, 1.2 equiv) was added dropwise, and the reaction mixture was stirred at −78 °C to −40 °C for 2.5 h. This was followed by workup, concentration and column chromatography, which afforded 1.69 g of compound **2g** as a colorless liquid (38.8% yield, 7.76 mmol). *R*_f_ = 0.58 (10% Et_2_O in hexanes). Spectroscopic data are similar to those reported in the literature.^11^

##### Spectroscopic Data for Compound **2g**

^1^H NMR (500 MHz, C_6_D_6_) δ = 7.58–7.51 (m, 2H, phenyl H), 7.25–7.17 (m, 3H, phenyl H), 4.84 (td, J = 1.8, 1.0 Hz, 1H, vinyl H), 4.73 (q, J = 1.5 Hz, 1H, vinyl H), 3.85 (s, 1H, CH), 1.42 (s, 3H, vinyl CH_3_), 1.08 (s, 1H, OH), 0.36 (s, 3H, Si­(CH_3_)), 0.32 (s, 3H, Si­(CH_3_)). ^13^C­{^1^H} NMR (126 MHz, C_6_D_6_) δ = 148.3, 137.4, 134.6, 129.6, 128.1, 107.2, 71.1, 21.0, −4.9, −5.3. ^29^Si NMR (99 MHz, C_6_D_6_) δ = −4.9. IR (neat, cm^–1^): 3439 (broad).

#### Synthesis of 2-Methyl-1-(methyldiphenylsilyl)­prop-2-en-1-ol (**2h**)

Following general procedure B, 2-methyl allyl alcohol (1.69 mL, 20.0 mmol, 1.0 equiv) in THF (100 mL) was cooled to −78 °C. To this solution, *n*-BuLi (9.6 mL, 2.5 M in hexane, 24.0 mmol, 1.2 equiv) was added dropwise and stirred at −78 °C for 1 h. After 1 h, chloro­(methyl)­diphenylsilane (4.2 mL, 20.0 mmol, 1.0 equiv) was dropwise added, and the reaction mixture was stirred at −78 °C to room temperature for 18 h. The reaction mixture was further cooled down to −78 °C, and *t*-BuLi (14.1 mL, 1.7 M in pentane, 24.0 mmol, 1.2 equiv) was added dropwise, and the reaction mixture was stirred at −78 °C to −40 °C for 2.5 h. This was followed by workup, concentration and column chromatography, which afforded 1.25 g of compound **2h** as a colorless liquid (23.2% yield, 4.64 mmol). *R*_f_ = 0.4 (12% Et_2_O in hexanes).

##### Spectroscopic Data for Compound **2h**

^1^H NMR (500 MHz, C_6_D_6_) δ = 7.76–7.53 (m, 4H, phenyl H), 7.19–7.10 (m, 6H, phenyl H), 4.87–4.84 (m, 1H, vinyl H), 4.72 (d, *J* = 1.6 Hz, 1H, vinyl H), 4.22 (s, 1H, CH), 1.34 (s, 3H, vinyl CH_3_), 1.19 (s, 1H, OH), 0.55 (s, 3H, Si­(CH_3_)). ^13^C­{^1^H} NMR (126 MHz, C_6_D_6_) δ = 148.0, 135.8, 135.5, 135.5, 129.8, 128.1, 128.1, 108.4, 70.4, 21.3, −6.4. ^29^Si NMR (99 MHz, C_6_D_6_) δ = −11.2. IR (neat, cm^–1^): 3403 (broad). HRMS (EI): *m*/*z* calcd for C_17_H_20_OSi [M]^+^: 268.1234; found: 268.1271.

#### Synthesis of 3-Methyl-2-methylene-1-(trimethylsilyl)­butan-1-ol (**2i**)

Following general procedure B, a solution of **1b** and **1c** alcohol as a 5:1 mixture (598 mg, 5.9 mmol, 1.0 equiv) in THF (50 mL) was cooled to −78 °C. To this solution, *n*-BuLi (2.86 mL, 2.5 M in hexane, 7.16 mmol, 1.2 equiv) was added slowly dropwise using a syringe and stirred at −78 °C for 1 h. After 1 h, chlorotrimethylsilane (0.74 mL, 5.9 mmol, 1.0 equiv) was dropwise added, and the reaction mixture was stirred at −78 °C for 1.5 h. The reaction mixture was further cooled down to −78 °C, and *t*-BuLi (4.2 mL, 1.7 M in pentane, 7.16 mmol, 1.2 equiv) was added dropwise, and the reaction mixture was stirred at −78 °C to −40 °C for 2.5 h. This was followed by workup, concentration and column chromatography, which afforded 215 mg of compound **2i** as a colorless liquid (21% yield, 1.25 mmol). The silanol from **1c** was also formed but not isolated.

##### Spectroscopic Data for Compound **2i**

^1^H NMR (500 MHz, C_6_D_6_) δ = 4.94 (t, *J* = 1.3 Hz, 1H, vinyl H), 4.82 (d, *J* = 1.1 Hz, 1H, vinyl H), 3.74 (s, 1H, CH), 1.86 (hept, *J* = 6.8 Hz, 1H, isopropyl CH), 1.03 (d, *J* = 6.8 Hz, 3H, isopropyl CH_3_), 0.98 (d, *J* = 6.8 Hz, 3H, isopropyl CH_3_), 0.09 (s, 9H, Si­(Me)_3_). ^13^C­{^1^H} NMR (126 MHz, C_6_D_6_) δ = 160.3, 102.8, 70.2, 31.6, 24.0, 21.3, −3.3. ^29^Si NMR (99 MHz, C_6_D_6_) δ = 1.8. IR (neat, cm^–1^): 3449 (broad). HRMS (EI): *m*/*z* calcd for C_9_H_20_OSi [M]^+^: 172.1283; found: 172.1274.

#### Synthesis of 3-Methyl-2-methylene-1-(triethylsilyl)­butan-1-ol (**2j**)

Following general procedure B, a solution of **1b** and **1c** alcohol as a 5:1 mixture (5.76 g, 57.6 mmol, 1.0 equiv) in THF (200 mL) was cooled to −78 °C. To this solution, *n*-BuLi (27 mL, 2.5 M in hexanes, 69.12 mmol, 1.2 equiv) was added dropwise using a syringe and stirred at −78 °C for 18 h. After 1 h, chlorotriethyl silane (4.2 mL, 20.0 mmol, 1.0 equiv) was dropwise added, and the reaction mixture was stirred at −78 °C to room temperature for 18 h. The reaction mixture was further cooled down to −78 °C, and *t*-BuLi (40.0 mL, 1.7 M in pentane, 69.12 mmol, 1.2 equiv) was added dropwise using a cannula over 45 min. The reaction mixture was stirred at −78 °C to −40 °C for 2.5 h. This was followed by workup, concentration and column chromatography (15% Et_2_O in hexanes), which afforded 6.16 g of **2j** and **2ja** as a 2:1 mixture (50% yield, 28.7 mmol) and 910 mg of pure **1b** (15%, 9.1 mmol) starting material was also isolated. The desired alcohol **2j** was further purified by another column on the mixture to afford 712 mg as a colorless liquid (5.7% yield, 3.32 mmol).

##### Spectroscopic Data for Compound **2j**

^1^H NMR (500 MHz, C_6_D_6_) δ = 4.99 (dd, *J* = 1.6, 1.0 Hz, 1H, vinyl H), 4.84 (q, *J* = 1.0 Hz, 1H), vinyl H, 3.94 (s, 1H, CH), 1.91 (hept, *J* = 6.8 Hz, 1H isopropyl CH), 1.08–1.02 (m, 12H, Si­(Me)_3_ & isopropyl CH_3_), 0.99 (d, *J* = 6.8 Hz, 3H, isopropyl CH_3_), 0.89 (s, 1H, OH), 0.67 (dddd, *J* = 22.9, 14.8, 7.9, 6.9 Hz, 6H, triethyl CH_2_). ^13^C­{^1^H} NMR (126 MHz, C_6_D_6_) δ = 160.7, 103.0, 68.5, 31.4, 24.1, 21.5, 7.9, 2.4. ^29^Si NMR (99 MHz, C_6_D_6_) δ = 5.7. IR (neat, cm^–1^): 3458 (broad). HRMS (EI): *m*/*z* calcd for C_12_H_26_OSi [M]^+^: 214.1752; found: 214.1748.

#### Synthesis of (*E*)-2-Methyl-1-(triethylsilyl)­but-2-en-1-ol (**2k**)

Following general procedure B, freshly prepared (*E*)-2-methylbut-2-en-1-ol **1d** (333.0 mg, 3.86 mmol, 1.0 equiv) in THF (20 mL) was cooled to −78 °C. To this solution, *n*-BuLi (1.85 mL, 2.5 M in hexane, 4.6 mmol, 1.2 equiv) was added dropwise and stirred at −78 °C for 1 h. After 1 h, chlorotriethylsilane (0.65 mL, 3.86 mmol, 1.0 equiv) was dropwise added, and the reaction mixture was stirred at −78 °C to room temperature for 18 h. The reaction mixture was further cooled down to −78 °C, and *t*-BuLi (2.72 mL, 1.7 M in pentane, 4.6 mmol, 1.2 equiv) was added dropwise, and the reaction mixture was stirred at −78 °C to −40 °C for 4 h. This was followed by workup, concentration and column chromatography (10% Et_2_O in hexanes), which afforded 75 mg of compound **2k** as a colorless liquid (9.7% yield, 0.37 mmol).

##### Spectroscopic Data for Compound **2k**

^1^H NMR (500 MHz, C_6_D_6_) δ = 5.40 (qt, *J* = 6.7, 1.4 Hz, 1H, vinyl H), 3.90 (s, 1H, CH), 1.66 (dt, *J* = 6.7, 1.1 Hz, 3H, vinyl CH_3_), 1.63 (d, *J* = 1.2 Hz, 3H, vinyl CH_3_), 1.15 (t, *J* = 7.9 Hz, 9H, triethyl CH_3_), 0.83 (t, *J* = 2.3 Hz, 1H, OH), 0.80–0.70 (m, 6H, triethyl CH_2_). ^13^C­{^1^H} NMR (126 MHz, C_6_D_6_) δ = 139.2, 115.7, 71.1, 14.6, 13.2, 7.8, 2.6. ^29^Si NMR (99 MHz, C_6_D_6_) δ = 4.9. IR (neat, cm^–1^): 3403 (broad). HRMS (EI): *m*/*z* calcd for C_11_H_24_OSi [M]^+^: 200.1596; found: 200.1586.

#### Synthesis of (*E*)-2-Methyl-1-(trimethylsilyl)­but-2-en-1-ol (**2l**)

Following general procedure B, freshly prepared (*E*)-2-methylbut-2-en-1-ol **1d** (861.3 mg, 10.0 mmol, 1.0 equiv) in THF (40 mL) was cooled to −78 °C. To this solution, *n*-BuLi (4.80 mL, 2.5 M in hexane, 12.0 mmol, 1.2 equiv) was added dropwise and stirred at −78 °C for 1 h. After 1 h, chloro trimethylsilane (1.27 mL, 10.0 mmol, 1.0 equiv) was dropwise added, and the reaction mixture was stirred at −78 °C for 1.5 h. *t*-BuLi (7.0 mL, 1.7 M in pentane, 12.0 mmol, 1.2 equiv) was added dropwise, and the reaction mixture was stirred at −78 °C for 4 h. This was followed by workup, concentration and column chromatography (10% Et_2_O in hexanes), which afforded 284 mg of compound **2l** as a colorless liquid (17.9% yield, 1.79 mmol). Spectroscopic data are similar to those reported in the literature.

##### Spectroscopic Data for Compound **2l**

^1^H NMR (500 MHz, C_6_D_6_) δ = 5.28 (qt, *J* = 6.7, 1.3 Hz, 1H, vinyl H), 3.63 (s, 1H, CH), 1.57–1.54 (m, 3H, vinyl CH_3_), 1.50 (s, 3H, vinyl CH_3_), 0.85 (s, 1H, OH), 0.09 (s, 9H, Si­(Me)_3_). ^13^C­{^1^H} NMR (126 MHz, C_6_D_6_) δ = 138.9, 115.7, 72.7, 14.6, 13.2, −3.1. ^29^Si NMR (99 MHz, C_6_D_6_) δ = 1.2.

#### Synthesis of 1-(Dimethyl­(phenyl)­silyl)­cyclohex-2-en-1-ol (**2m**)

Following general procedure D, freshly prepared PhMe_2_SiLi solution was added to cyclohex-2-en-1-one (0.3 mL, 3 mmol, 1.0 equiv) in THF (10 mL) at −78 °C and stirred for 40 min.This was followed by workup, concentration and column chromatography (2% EtOAc in hexanes), which afforded 770 mg of compound **2m** as a colorless liquid (>99% yield, 3.3 mmol). Spectroscopic data are similar to those reported in the literature.^37^

##### Spectroscopic Data for Compound **2m**

^1^H NMR (500 MHz, CDCl_3_) δ = 7.61 (dd, *J* = 7.4, 1.9 Hz, 2H, phenyl H), 7.39–7.35 (m, 3H, phenyl H), 5.85 (ddd, *J* = 9.9, 4.9, 2.8 Hz, 1H, vinyl H), 5.72 (dt, *J* = 10.0, 2.1 Hz, 1H, vinyl H), 2.05–1.99 (m, 1H, CH), 1.87–1.80 (m, 1H), 1.72 (dd, *J* = 8.4, 4.7 Hz, 2H, cyclohexene CH_2_), 1.6–1.51 (m, 2H, cyclohexene CH_2_), 1.21 (s, 1H, OH), 0.37 (s, 3H Si­(Me)), 0.36 (s, 3H, Si­(Me)). ^13^C­{^1^H} NMR (126 MHz, CDCl_3_) δ = 136.5, 134.7, 130.6, 130.4, 129.4, 127.8, 64.3, 32.8, 25.3, 17.6, −5.6, −5.8. ^29^Si NMR (99 MHz, CDCl_3_) δ = −2.3.

#### Synthesis of 1-(Dimethyl­(phenyl)­silyl)-2-methylcyclohex-2-en-1-ol (**2n**)

Following general procedure D, freshly prepared PhMe_2_SiLi solution was added to 2-methylcyclohex-2-en-1-one (659 mg, 6 mmol, 1.0 equiv) in THF (25 mL) at −78 °C and stirred for 1 h.This was followed by workup, concentration and column chromatography (100% hexanes to 2% EtOAc in hexanes), which afforded 560 mg of compound **2n** as a yellow liquid (37.8% yield, 2.27 mmol). Spectroscopic data are similar to those reported in the literature.^38^

##### Spectroscopic Data for Compound **2n**

^1^H NMR (500 MHz, C_6_D_6_) δ = 7.69–7.66 (m, 2H, phenyl H), 7.25–7.20 (m, 3H, phenyl H), 5.33 (tt, *J* = 3.2, 1.5 Hz, 1H, vinyl H), 1.85–1.75 (m, 1H), 1.76–1.71 (m, 4H), 1.69–1.62 (m, 1H), 1.48 (ddd, *J* = 12.8, 8.7, 3.3 Hz, 1H), 1.36–1.23 (m, 2H, cyclohexene CH_2_), 0.88 (d, *J* = 3.1 Hz, 1H), 0.45 (s, 3H, Si­(Me)), 0.37 (s, 3H, Si­(Me)). ^13^C­{^1^H} NMR (126 MHz, C_6_D_6_) δ = 138.6, 138.3, 134.9, 129.3, 128.0, 124.3, 68.4, 36.2, 25.7, 21.5, 18.8, −3.2, −3.5. ^29^Si NMR (99 MHz, C_6_D_6_) δ = −1.6.

#### Synthesis of 2-Methyl-2-(triethylsilyl)­propanal (**3a**)

To a 4 mL vial with a magnetic stir bar, 2-methyl-1-(triethylsilyl)­prop-2-en-1-ol **2a** (56.0 mg, 0.3 mmol, 1.0 equiv) was added. This was followed by adding a freshly prepared solution of 0.001 M HCl in chloroform (0.5 mL). The reaction mixture was stirred at room temperature for 1 h. The resulting mixture was concentrated under reduced pressure, which afforded 55.0 mg of pure compound **3a** as a colorless liquid (98% yield, 0.29 mmol).

##### Spectroscopic Data for Compound **3a**

^1^H NMR (500 MHz, C_6_D_6_) δ = 9.53 (s, 1H, aldehyde H), 1.03 (s, 6H, 2­(CH_3_)), 0.84 (t, *J* = 8.0 Hz, 9H, triethyl CH_3_), 0.45 (q, *J* = 8.0 Hz, 6H, triethyl CH_2_). ^13^C­{^1^H} NMR (126 MHz, C_6_D_6_) δ = 204.4, 42.3, 18.5, 7.9, 2.0. ^29^Si NMR (99 MHz, C_6_D_6_) δ = 10.4. IR (neat, cm^–1^): 1696. HRMS (EI): *m*/*z* calcd for C_10_H_22_OSi [M]^+^: 186.1439; found: 186.1429.

#### Synthesis of 2-Methyl-2-(Trimethylsilyl)­propanal (**3b**)

To a 4 mL vial with a magnetic stir bar, 2-methyl-1-(trimethylsilyl)­prop-2-en-1-ol **2b** (144.29 mg, 1.0 mmol, 1.0 equiv) was added. This was followed by adding a freshly prepared solution of 0.001 M HCl in chloroform (1.5 mL). The reaction mixture was stirred at room temperature for 10 min. The resulting mixture was concentrated under reduced pressure, which afforded 51.0 mg of pure compound **3b** as a colorless liquid (35% yield, 0.35 mmol). (Note: We attribute the low yield to product volatility).

##### Spectroscopic Data for Compound **3b**

^1^H NMR (500 MHz C_6_D_6_) δ = 9.46 (s, 1H, aldehyde H), 0.96 (s, 6H, 2­(CH_3_)), −0.17 (s, 9H, Si­(Me_3_)). ^13^C­{^1^H} NMR (126 MHz, C_6_D_6_) δ = 204.8, 17.4, −4.4. ^29^Si NMR (99 MHz, C_6_D_6_) δ = 8.1. IR (neat, cm^–1^): 1663. HRMS (EI): *m*/*z* calcd for C_7_H_16_OSi [M]^+^: 144.0970; found: 144.0963.

#### Synthesis of 2-Methyl-2-(tripropylsilyl)­propanal (**3c**)

To a 4 mL vial with a magnetic stir bar, 2-methyl-1-(tripropylsilyl)­prop-2-en-1-ol **2c** (45.7 mg, 0.2 mmol, 1.0 equiv) was added. This was followed by adding a freshly prepared solution of 0.001 M HCl in chloroform (0.5 mL). The reaction mixture was stirred at room temperature for 1 h. The resulting mixture was concentrated under reduced pressure and passed through a deactivated (with triethyl amine) silica plug using chloroform, which afforded 33.2 mg of pure compound **3c** as a colorless liquid (72% yield, 0.14 mmol).

##### Spectroscopic Data for Compound **3c**

^1^H NMR (500 MHz, C_6_D_6_) δ = 9.54 (s, 1H, aldehyde H), 1.31–1.14 (m, 6H, *n*-Pr CH_2_), 1.05 (s, 6H, 2­(CH_3_)), 0.91 (t, *J* = 7.2 Hz, 9H, *n*-Pr CH_3_), 0.51–0.46 (m, 6H, *n*-Pr CH_2_). ^13^C­{^1^H} NMR (126 MHz C_6_D_6_) δ = 204.4, 42.2, 18.9, 18.6, 18.0, 13.9. ^29^Si NMR (99 MHz, C_6_D_6_) δ = 6.1. IR (neat, cm^–1^): 1697. HRMS (EI): *m*/*z* calcd for C_13_H_28_OSi [M]^+^: 228.1909; found: 228.1900.

#### Synthesis of 2-Methyl-2-(tributylsilyl)­propanal (**3d**)

To a 4 mL vial with a magnetic stir bar, 2-methyl-1-(tributylsilyl)­prop-2-en-1-ol **2d** (81.0 mg, 0.3 mmol, 1.0 equiv) was added. This was followed by adding a freshly prepared solution of 0.001 M HCl in chloroform (0.5 mL). The reaction mixture was stirred at room temperature for 30 min. The resulting mixture was concentrated under reduced pressure and passed through a silica plug, which afforded 57.0 mg of compound **3d** as a colorless liquid (70% yield, 0.21 mmol).

##### Spectroscopic Data for Compound **3d**

^1^H NMR (500 MHz, C_6_D_6_ + 1% TMS) δ = 9.59 (s, 1H, aldehyde H), 1.32–1.24 (m, 12H, *n*-Bu (CH_2_)), 1.09 (s, 6H, 2­(CH_3_)), 0.88 (t, J = 6.9 Hz, 9H, *n*-Bu (CH_3_)), 0.59–0.55 (m, 6H, *n*-Bu (CH_2_)). ^13^C­{^1^H} NMR (126 MHz, C_6_D_6_) δ = 204.5, 42.3, 27.3, 26.6, 18.7, 13.9, 10.8. ^29^Si NMR (99 MHz, C_6_D_6_) δ = 7.1. IR (neat, cm^–1^): 1691. HRMS (EI): *m*/*z* calcd for C_16_H_34_OSi [M]^+^: 270.2378; found: 270.2359.

#### Synthesis of 2-Methyl-2-(triisopropylsilyl)­propanal (**3e**)

To a 4 mL vial with a magnetic stir bar, 2-methyl-1-(triisopropylsilyl)­prop-2-en-1-ol **2e** (68.0 mg, 0.3 mmol, 1.0 equiv) was added. This was followed by adding a freshly prepared solution of 0.001 M HCl in chloroform (0.5 mL). The reaction mixture was stirred at room temperature for 30 min. The resulting mixture was concentrated under reduced pressure and passed through a silica plug, which afforded 45.0 mg of pure compound **3e** as a colorless liquid (66% yield, 0.19 mmol). Spectroscopic data are similar to those reported in the literature.^39^

##### Spectroscopic Data for **3e**

^1^H NMR (500 MHz, C_6_D_6_) δ = 9.64 (s, 1H, aldehyde H), 1.14 (s, 6H, 2­(CH_3_)), 1.03–0.97 (m, 24H, *i*-Pr (CH & CH_3_). ^13^C­{^1^H} NMR (126 MHz, C_6_D_6_) δ = 204.1, 43.2, 20.5, 19.4, 11.8. ^29^Si NMR (99 MHz, C_6_D_6_) δ = 1.4. IR (neat, cm^–1^): 1682.

#### Synthesis of 2-(*tert*-Butyldimethylsilyl)-2-methylpropanal (**3f**)

To a 4 mL vial with a magnetic stir bar, 1-(*tert*-butyldimethylsilyl)-2-methylprop-2-en-1-ol **2f** (55.9 mg, 0.3 mmol, 1.0 equiv) was added. This was followed by adding a freshly prepared solution of 0.001 M HCl in chloroform (0.5 mL). The reaction mixture was stirred at room temperature for 30 min. The resulting mixture was concentrated under reduced pressure and passed through a silica plug, which afforded 44.0 mg of compound **3f** as a white solid (78.6% yield, 0.23 mmol). Spectroscopic data are similar to those reported in the literature.^40^

##### Spectroscopic Data for Compound **3f**

^1^H NMR (500 MHz, C_6_D_6_) δ = 9.58 (s, 1H, aldehyde H), 1.03 (s, 6H, 2­(CH_3_)), 0.81 (s, 9H, *t*-Bu (CH_3_), −0.14 (s, 6H, Si­(CH_3_)_2_. ^13^C­{^1^H} NMR (126 MHz, C_6_D_6_) δ = 204.3, 42.7, 27.8, 19.2, 18.9, −7.5. ^29^Si NMR (99 MHz, C_6_D_6_) δ = 13.0. IR (neat, cm^–1^): 1673.

#### Synthesis of 2-(Dimethyl­(phenyl)­silyl)-2-methylpropanal (**3g**)

To a 4 mL vial with a magnetic stir bar, 1-(dimethyl­(phenyl)­silyl)-2-methylprop-2-en-1-ol **2g** (61.0 mg, 0.3 mmol, 1.0 equiv) was added. This was followed by adding a freshly prepared solution of 0.001 M HCl in chloroform (0.5 mL). The reaction mixture was stirred at room temperature for 30 min. The resulting mixture was concentrated under reduced pressure and passed through a deactivated silica plug, which afforded 28.0 mg of compound **3g** as a colorless liquid (67.8% yield, 0.14 mmol).

##### Spectroscopic Data for Compound **3g**

^1^H NMR (500 MHz, C_6_D_6_) δ = 9.48 (s, 1H, aldehyde H), 7.49–7.45 (m, 2H, phenyl H), 7.42–7.36 (m, 3H, phenyl H), 1.18 (s, 6H, 2­(CH_3_)), 0.37 (s, 6H, Si­(CH_3_)_2_). ^13^C­{^1^H} NMR (126 MHz, C_6_D_6_) δ = 207.1, 134.5, 129.9, 128.1, 42.3, 17.7, – 5.9. ^29^Si NMR (99 MHz, C_6_D_6_) δ = 1.7. IR (neat, cm^–1^): 1688. HRMS (EI): *m*/*z* calcd for C_12_H_18_OSi [M]^+^: 206.1126; found: 206.1120.

#### Synthesis of 2-Methyl-2-(methyldiphenylsilyl)­propanal (**3h**)

To a 4 mL vial with a magnetic stir bar, 2-methyl-1-(methyldiphenylsilyl)­prop-2-en-1-ol **2h** (62.0 mg, 0.23 mmol, 1.0 equiv) was added. This was followed by adding a freshly prepared solution of 0.001 M HCl in chloroform (0.5 mL). The reaction mixture was stirred at room temperature for 2 h. The resulting mixture was concentrated under reduced pressure and passed through a silica plug, which afforded 41.0 mg of compound **3h** as a white solid (66% yield, 0.15 mmol).

##### Spectroscopic Data for Compound **3h**

^1^H NMR (500 MHz, CDCl_3_) δ = 9.61 (s, 1H, aldehyde H), 7.60–7.56 (m, 4H, phenyl H), 7.43–7.37 (m, 6H, phenyl H), 1.29 (s, 6H, 2­(CH_3_)), 0.67 (s, 3H, Si­(CH_3_)). ^13^C­{^1^H} NMR (126 MHz, CDCl_3_) δ = 206.9, 135.4, 133.4, 130.0, 128.2, 42.1, 18.7, −6.1. ^29^Si NMR (99 MHz, CDCl_3_) δ = −6.1. IR (neat, cm^–1^): 1691. HRMS (EI): *m*/*z* calcd for C_17_H_20_OSi [M]^+^: 268.1283; found: 268.1268.

#### Synthesis of 2,3-Dimethyl-2-(trimethylsilyl)­butanal (**3i**)

To a 4 mL vial with a magnetic stir bar, 3-methyl-2-methylene-1-(trimethylsilyl)­butan-1-ol **2i** (51.7 mg, 0.3 mmol, 1.0 equiv) was added. This was followed by adding a freshly prepared solution of 0.001 M HCl in chloroform (0.5 mL). The reaction mixture was stirred at room temperature for 30 min. The resulting mixture was concentrated under reduced pressure and passed through a silica plug, which afforded 36.0 mg of compound **3i** as a colorless liquid (69.6% yield, 0.20 mmol).

##### Spectroscopic Data for Compound **3i**

^1^H NMR (500 MHz, C_6_D_6_) δ = 9.45 (s, 1H, aldehyde H), 2.27 (hept, *J* = 6.9 Hz, 1H, *i*-Pr CH), 0.95 (s, 3H, CH_3_), 0.74 (d, *J* = 7.0 Hz, 3H, *i*-Pr CH_3_), 0.70 (d, *J* = 6.8 Hz, 3H, *i*-Pr CH_3_), −0.09 (s, 9H, Si­(CH_3_)_3_). ^13^C­{^1^H} NMR (126 MHz, C_6_D_6_) δ = 205.1, 50.3, 29.6, 19.4, 19.2, 9.0, −2.4. ^29^Si NMR (99 MHz, C_6_D_6_) δ = 5.6. IR (neat, cm^–1^): 1660. HRMS (EI): *m*/*z* calcd for C_9_H_20_OSi [M]^+^: 172.1283; found: 172.1277.

#### Synthesis of 2,3-Dimethyl-2-(triethylsilyl)­butanal (**3j**)

To a 4 mL vial with a magnetic stir bar, 3-methyl-2-methylene-1-(triethylsilyl)­butan-1-ol **2j** (43.0 mg, 0.3 mmol, 1.0 equiv) was added. This was followed by adding a freshly prepared solution of 0.001 M HCl in chloroform (0.5 mL). The reaction mixture was stirred at room temperature for 1 h. The resulting mixture was concentrated under reduced pressure and passed through a silica plug, which afforded 26.0 mg of compound **3j** as a colorless liquid (40.4% yield, 0.12 mmol).

##### Spectroscopic Data for Compound **3j**

^1^H NMR (500 MHz, C_6_D_6_) δ = 9.54 (s, 1H, aldehyde H), 2.28 (hept, *J* = 6.8 Hz, 1H, *i*-Pr CH), 1.02 (s, 3H, CH_3_), 0.88 (t, *J* = 7.9 Hz, 9H, triethyl CH_3_), 0.77 (d, *J* = 6.9 Hz, 3H, *i*-Pr CH_3_), 0.70 (d, *J* = 6.8 Hz, 3H, *i*-Pr CH_3_), 0.52 (qd, *J* = 7.8, 2.4 Hz, 6H, triethyl CH_2_). ^13^C­{^1^H} NMR (126 MHz, C_6_D_6_) δ = 204.3, 51.8, 29.7, 19.7, 19.3, 9.8, 8.1, 3.3. ^29^Si NMR (99 MHz, C_6_D_6_) δ = 8.6. IR (neat, cm^–1^): 1694. HRMS (EI): *m*/*z* calcd for C_12_H_26_OSi [M]^+^: 214.1752; found: 214.1748.

#### Synthesis of 2-Methyl-2-(triethylsilyl)­butanal (**3k**)

To a 4 mL vial with a magnetic stir bar, (*E*)-2-methyl-1-(triethylsilyl)­but-2-en-1-ol **2k** (60.1 mg, 0.3 mmol, 1.0 equiv) was added. This was followed by adding a freshly prepared solution of 0.001 M HCl in chloroform (0.5 mL). The reaction mixture was stirred at room temperature for 1 h. The resulting mixture was concentrated under reduced pressure and passed through a silica plug, which afforded 37.0 mg of compound **3k** as a colorless liquid (61.5% yield, 0.18 mmol).

##### Spectroscopic Data for Compound **3k**

^1^H NMR (500 MHz, C_6_D_6_) δ = 9.54 (s, 1H, aldehyde H), 1.95 (dq, *J* = 14.6, 7.4 Hz, 1H), 1.24 (dd, *J* = 13.9, 7.3 Hz, 1H), 1.07 (s, 3H, CH_3_), 0.85 (t, *J* = 7.9 Hz, 9H, triethyl CH_3_), 0.70 (t, *J* = 7.4 Hz, 3H, ethyl (CH_3_)), 0.47 (q, *J* = 8.2 Hz, 6H, triethyl CH_2_). ^13^C­{^1^H} NMR (126 MHz, C_6_D_6_) δ = 204.5, 47.6, 24.7, 13.5, 9.8, 8.0, 2.1.^29^Si NMR (99 MHz, C_6_D_6_) δ = 10.1. IR (neat, cm^–1^): 1690. HRMS (EI): *m*/*z* calcd for C_11_H_24_OSi [M]^+^: 200.1596; found: 200.1586.

#### Synthesis of 2-Methyl-2-(trimethylsilyl)­butanal (**3l**)

To a 4 mL vial with a magnetic stir bar, (*E*)-2-methyl-1-(trimethylsilyl)­but-2-en-1-ol **2l** (47.5 mg, 0.3 mmol, 1.0 equiv) was added. This was followed by adding a freshly prepared solution of 0.001 M HCl in chloroform (0.5 mL). The reaction mixture was stirred at room temperature for half hour. The resulting mixture was concentrated under reduced pressure and passed through a silica plug, which afforded 33 mg of compound **3l** and starting material **2l** as a mixture of (1:0.2) as a colorless liquid (69% yield, 0.20 mmol). Due to the low yield compound **3l** was not further purified.

##### Spectroscopic Data for Compound **3l**

^1^H NMR (500 MHz, CDCl_3_) δ = δ 9.55 (s, 1H, aldehyde H), 1.64–1.58 (m, 2H, CH_2_), 1.14 (s, 3H, CH_3_), 0.88 (t, *J* = 7.4 Hz, 3H, ethyl (CH_3_)), 0.06 (s, 9H, Si­(CH_3_)_3_).^13^C­{^1^H} NMR (126 MHz, CDCl_3_) δ = 207.2, 47.4, 24.1, 12.6, 10.2, −3.8. HRMS (EI): *m*/*z* calcd for C_8_H_18_OSi [M]^+^: 158.1126; found: 158.1119

#### Synthesis of 3-Methyl-2-methylene-1-(triethylsilyl)­butan-1-one (**2z**)

Following a reported procedure,^41^ in an oven-dried 100 mL round-bottom flask fitted with a magnetic stir bar, 25 mL of dry DCM was added. The flask was sealed with a rubber septum and purged with nitrogen. To this flask, trifluoroacetic anhydride (0.84 mL, 6.06 mmol, 1.5 equiv) was added via a syringe and the reaction mixture was cooled down to −78 °C by using a dry ice-acetone bath. This was followed by a dropwise addition of dimethyl sulfoxide (DMSO) (0.57 mL, 8.08 mmol, 2.0 equiv) using a syringe. After stirring at −78 °C for 30 min, 3-methyl-2-methylene-1-(triethylsilyl)­butan-1-ol **2i** (867 mg, 4.04 mmol, 1.0 equiv) was added to the reaction mixture as a solution in dry DCM, and the reaction mixture was further stirred at −78 °C for 1 h. Lastly, freshly distilled triethyl amine (1.69 mL, 12.12 mmol, 3.0 equiv) was slowly added via a syringe, and the resulting mixture was stirred at −78 °C for another hour. After 1 h, the reaction mixture was quenched by adding 20 mL DI water at −78 °C dropwise and allowed to warm up slowly to room temperature. This was transferred to a 250 mL separatory funnel, and the layers were separated. The aqueous layer was extracted twice with DCM, and the combined organic layers were dried over anhydrous sodium sulfate. The crude was obtained by evaporating the solvent on a rotary evaporator. Column chromatography (5% EtOAc in hexanes) was performed to obtain 719.0 mg of compound **2z** as a colorless liquid (83.7% yield, 3.38 mmol).

##### Spectroscopic Data for Compound **2z**

^1^H NMR (500 MHz, CDCl_3_) δ = 5.96 (d, *J* = 1.2 Hz, 1H, vinyl H), 5.90 (s, 1H, vinyl H), 2.85 (hept, *J* = 6.8 Hz, 1H, *i*-Pr CH), 0.99–0.94 (m, 13H), 0.94 (s, 2H), 0.81–0.76 (m, 6H). ^13^C­{^1^H} NMR (126 MHz, CDCl_3_) δ = 238.1, 161.7, 124.9, 26.3, 21.8, 7.5, 4.0.

#### Synthesis of (*R*)-3-Methyl-2-methylene-1-(triethylsilyl)­butan-1-ol ((+)-**2j**)

Following a reported procedure,^42^ in a 100 mL oven-dried round-bottom flask fitted with a magnetic stirrer, 2 g of 4Å molecular sieves was added. Under nitrogen atmosphere, compound **2z** (699 mg, 3.29 mmol, 1.0 equiv) was added as a solution in 15 mL freshly distilled THF and stirred at room temperature for 2 h. Later, (*R*)–CBS (4.6 g, 16.45 mmol, 5.0 equiv) was added, and the reaction mixture was cooled down to −30 °C using a dry ice-acetonitrile bath. After 10 min, BH_3_·SMe_2_ (1.6 mL, 16.45 mmol, 5.0 equiv) was added dropwise using a syringe at −30 °C. After stirring at this temperature for 15 min, the reaction mixture was quenched by adding methanol and diluted with ammonium chloride. This was followed by extraction using diethyl ether (20 mL × 2). The combined organic layers were washed with brine and dried over anhydrous sodium sulfate. This was followed by filtration, concentration under reduced pressure, and flash column chromatography (30% Et_2_O in hexanes followed by a second column in 100% hexanes) to afford 171.0 mg of compound (+)-**2j** as a colorless liquid (24.2% yield, 0.79 mmol).

Polarimeter data for compound (+)-**2j** [α]_D_20 = +67 (*c =* 0.1 mg/mL, CH_2_Cl_2_). The other spectral data were identical with those of compound **2j**. Upon rearrangement, this value plummeted to net [α]_D_20 = +0.6° (*c =* 0.1 mg/mL, CH_2_Cl_2_).

## Supplementary Material



## Data Availability

The data underlying this study are available in the published article and its Supporting Information.

## Notes

Copies of ^1^H, ^13^C and ^29^Si NMR spectra (PDF)
