# Supplementary material for: [1,2]-Carbon to Carbon Silyl Migration for Accessing α‑Silyl Alkanals from α‑Hydroxy Allyl Silanes
Source: J Org Chem. 2026 Jun 15;91(25):8670–82. doi: 10.1021/acs.joc.6c00817 (PMC13316976; doi:10.1021/acs.joc.6c00817)
Supplement: Supplementary file 1 [file jo6c00817_si_001.pdf]

Supporting Information

[1,2]-Carbon to carbon silyl migration for accessing α-silyl alkanals from α-hydroxy allyl silanes

Darshika Singh, Emmanuel W. Maloba and Robert E. Maleczka, Jr.*

Department of Chemistry, Michigan State University, 578 South Shaw Lane, East Lansing, Michigan 48824-1322 USA

Corresponding author : maleczka@chemistry.msu.edu

Table of Contents

Copies of ^{1}H, ^{13}C and ^{29}Si NMR spectra ………………… S3-S75

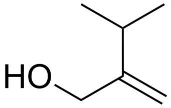

1b

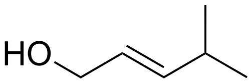

1c

(20:1)

^{1}H NMR (500 MHz, $CDCl_3$)

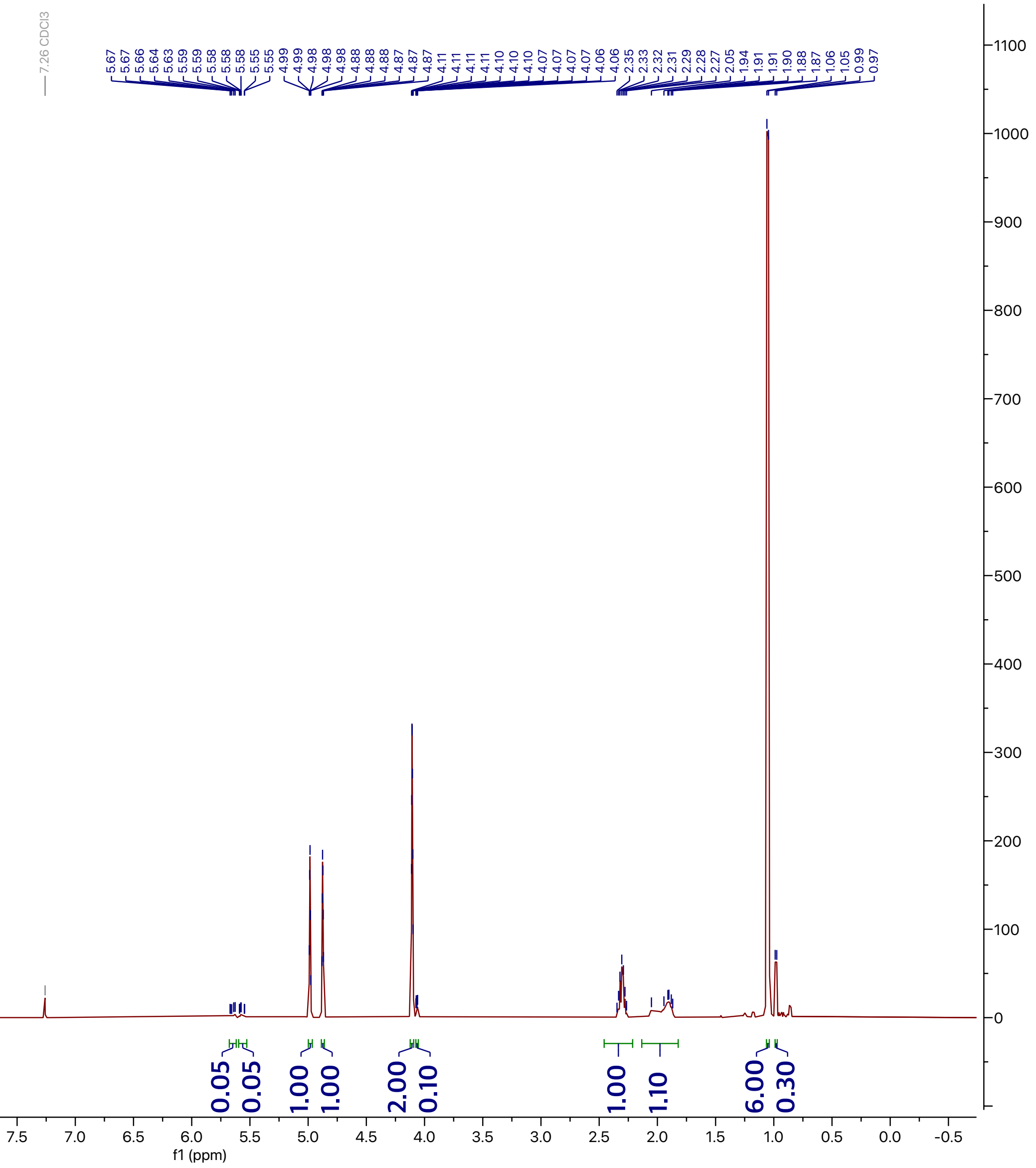

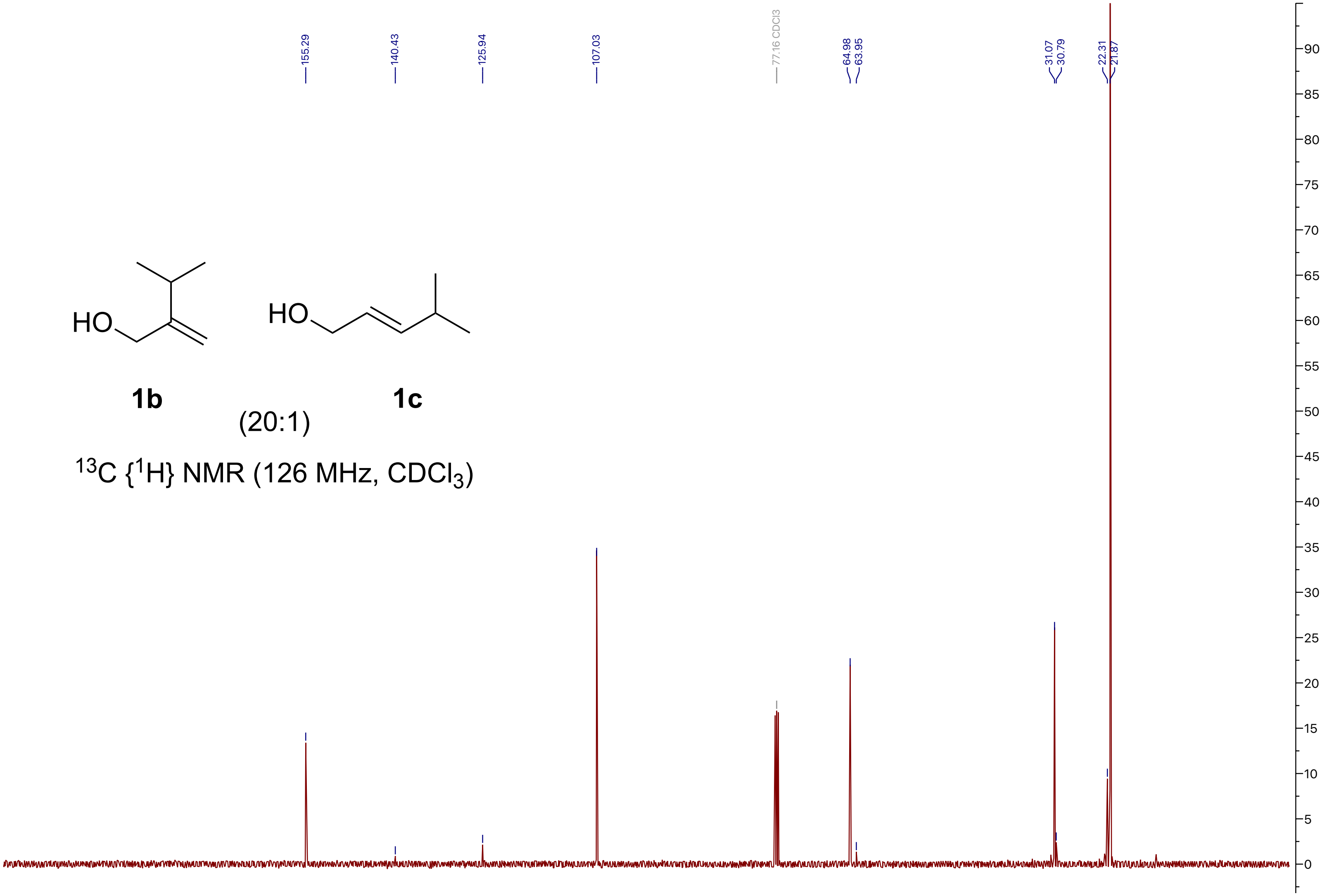
155.29
140.43
125.94
107.03
77.16 CDCl3
64.98
63.95
31.07
30.79
22.31
21.87
HO
HO
1b
(20:1)
1c
^{13}C {^{1}H} NMR (126 MHz, $CDCl_3$)
90
85
80
75
70
65
60
55
50
45
40
35
30
25
20
15
10
5
0

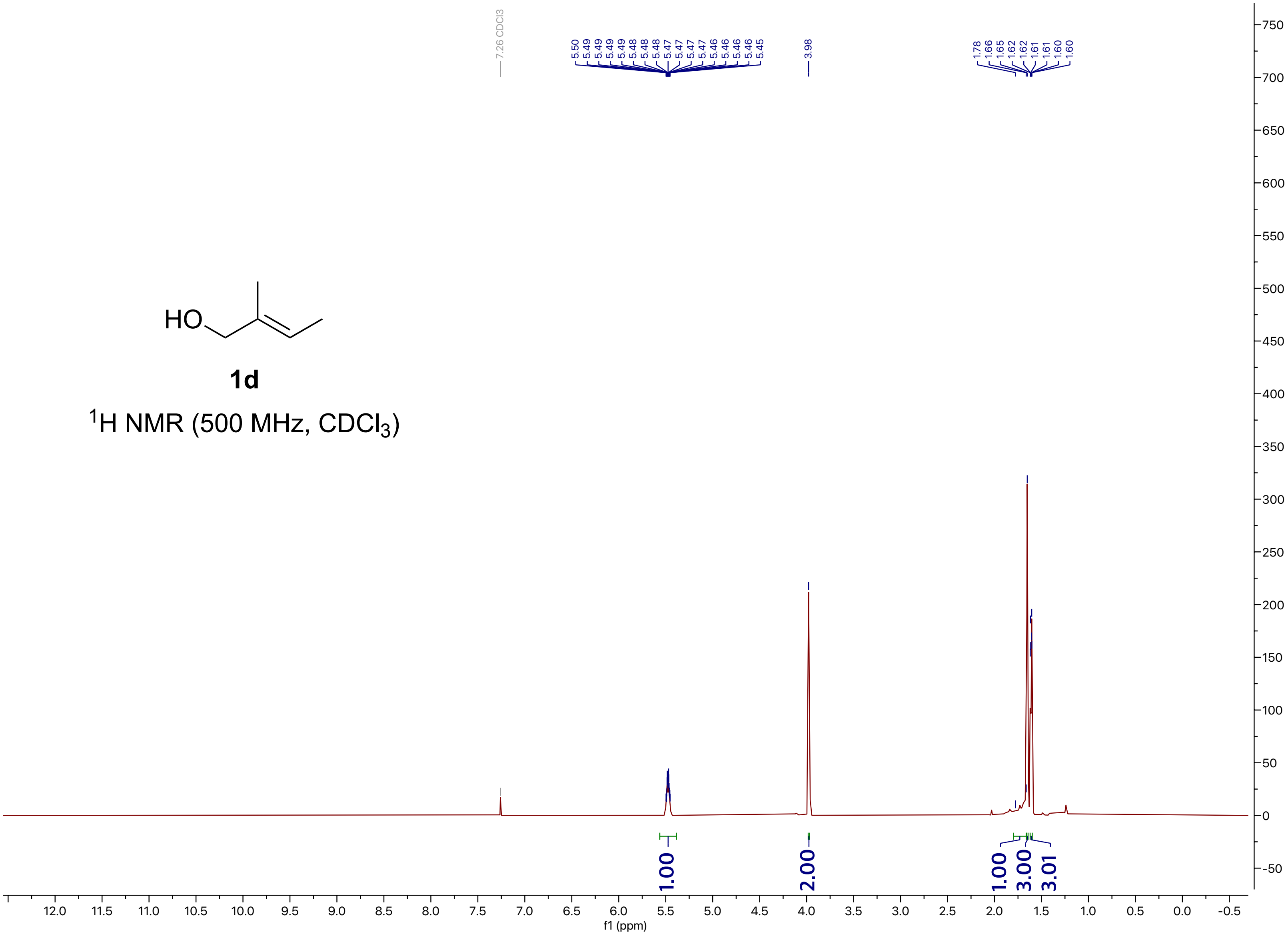

1d

^{1}H NMR (500 MHz, $CDCl_3$)

1-DS-297-col26-33_CARBON_01

1d

$^{13}C\ \{^{1}H\}$ NMR (126 MHz, $CDCl_3$)

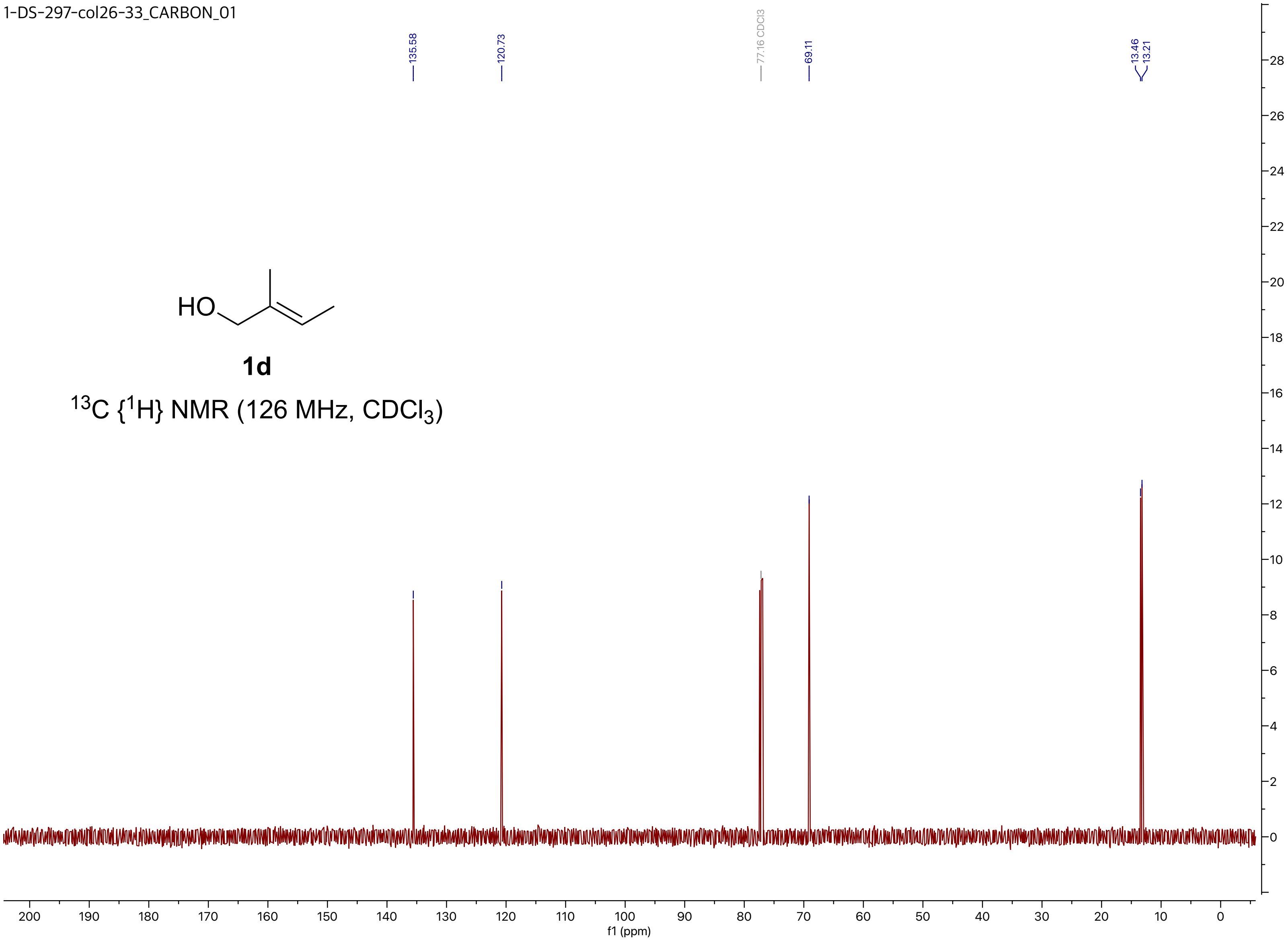

2a

^{1}H NMR (500 MHz, C_6D_6)

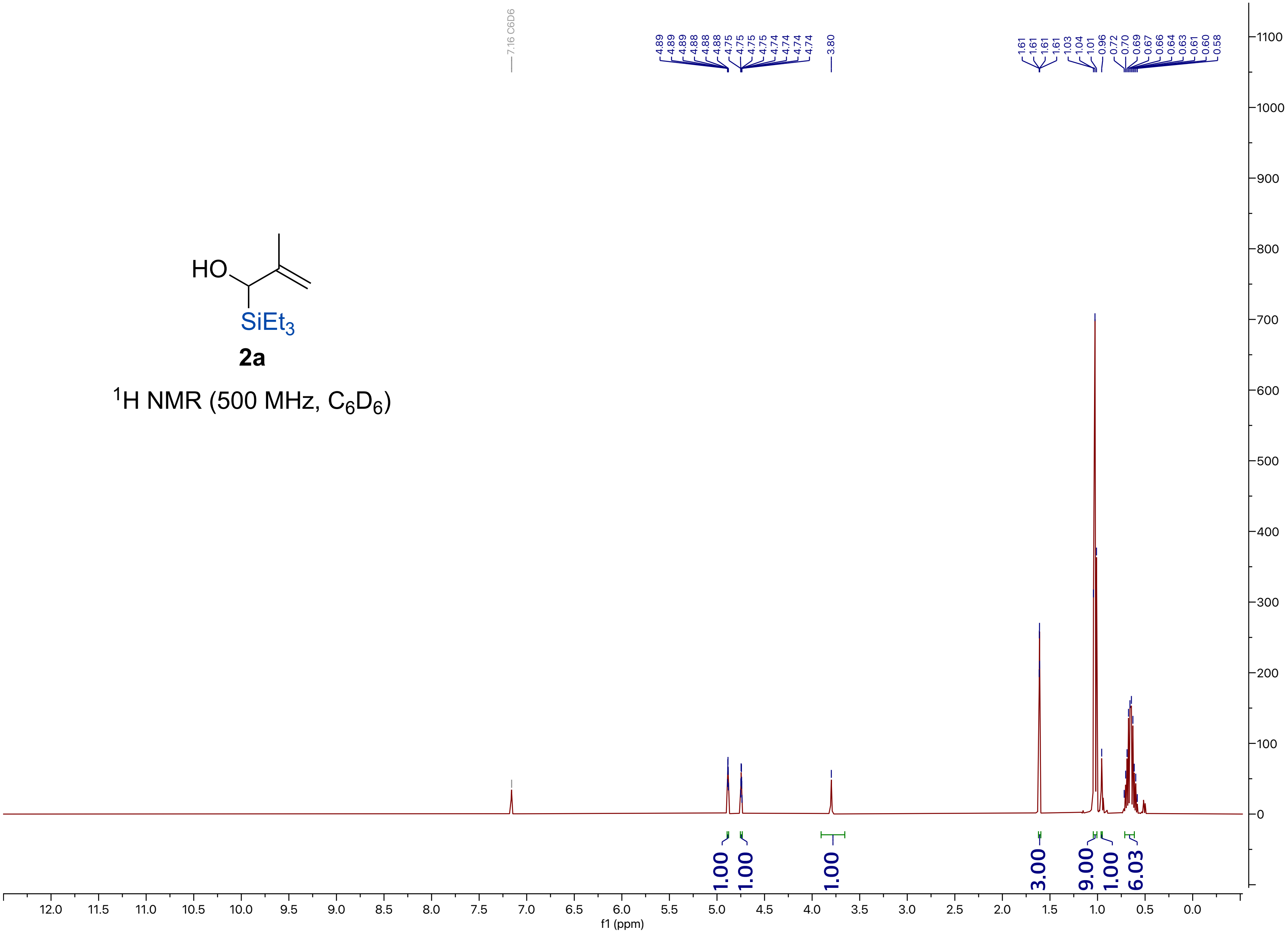

1-DS-278col2-34-last_CARBON_01

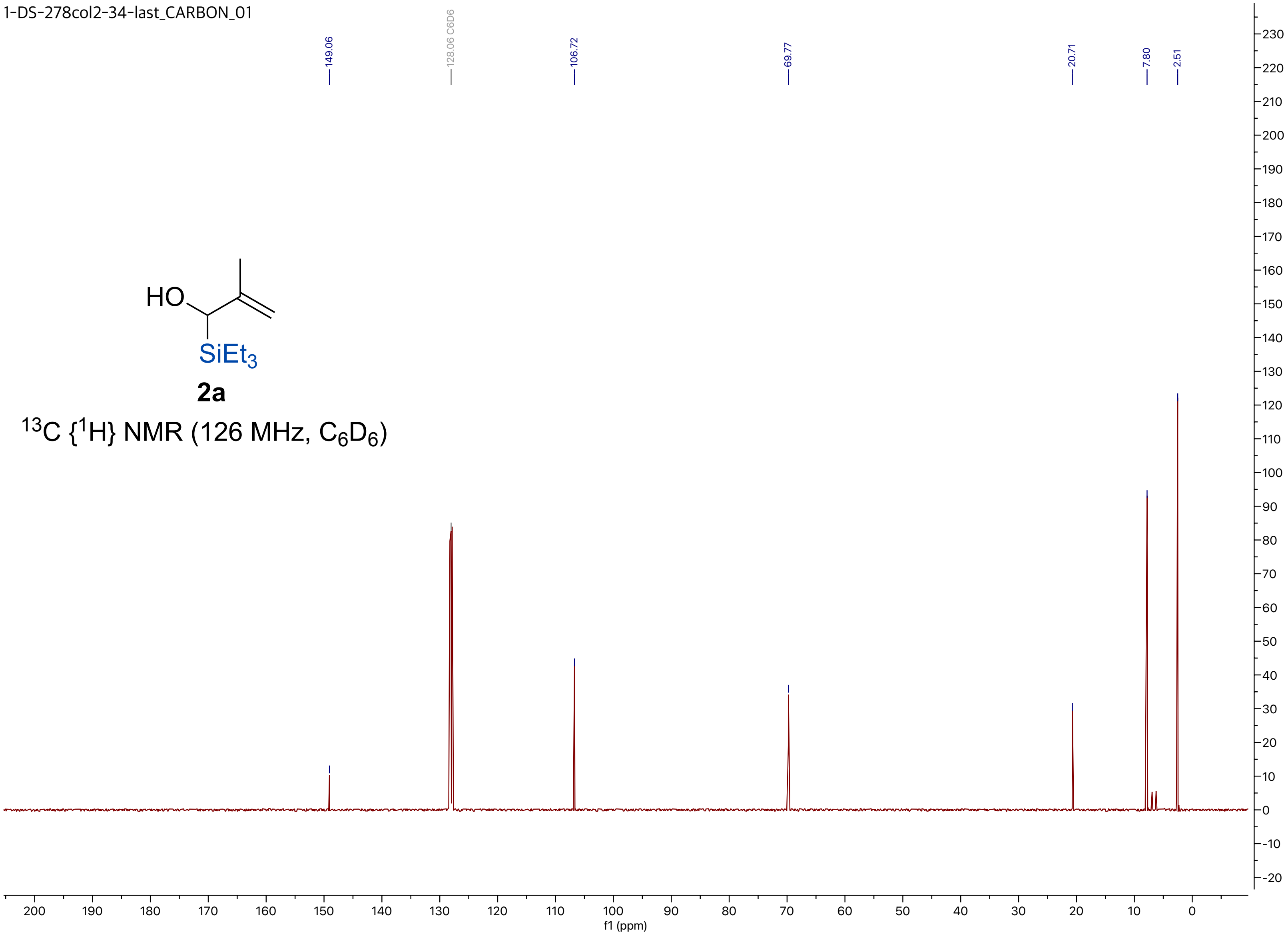

2a

$^{13}C\ \{^1H\}$ NMR (126 MHz, C_6D_6)

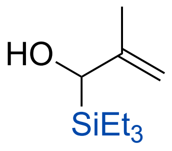

2a

^{29}Si NMR (99 MHz, C_6D_6)

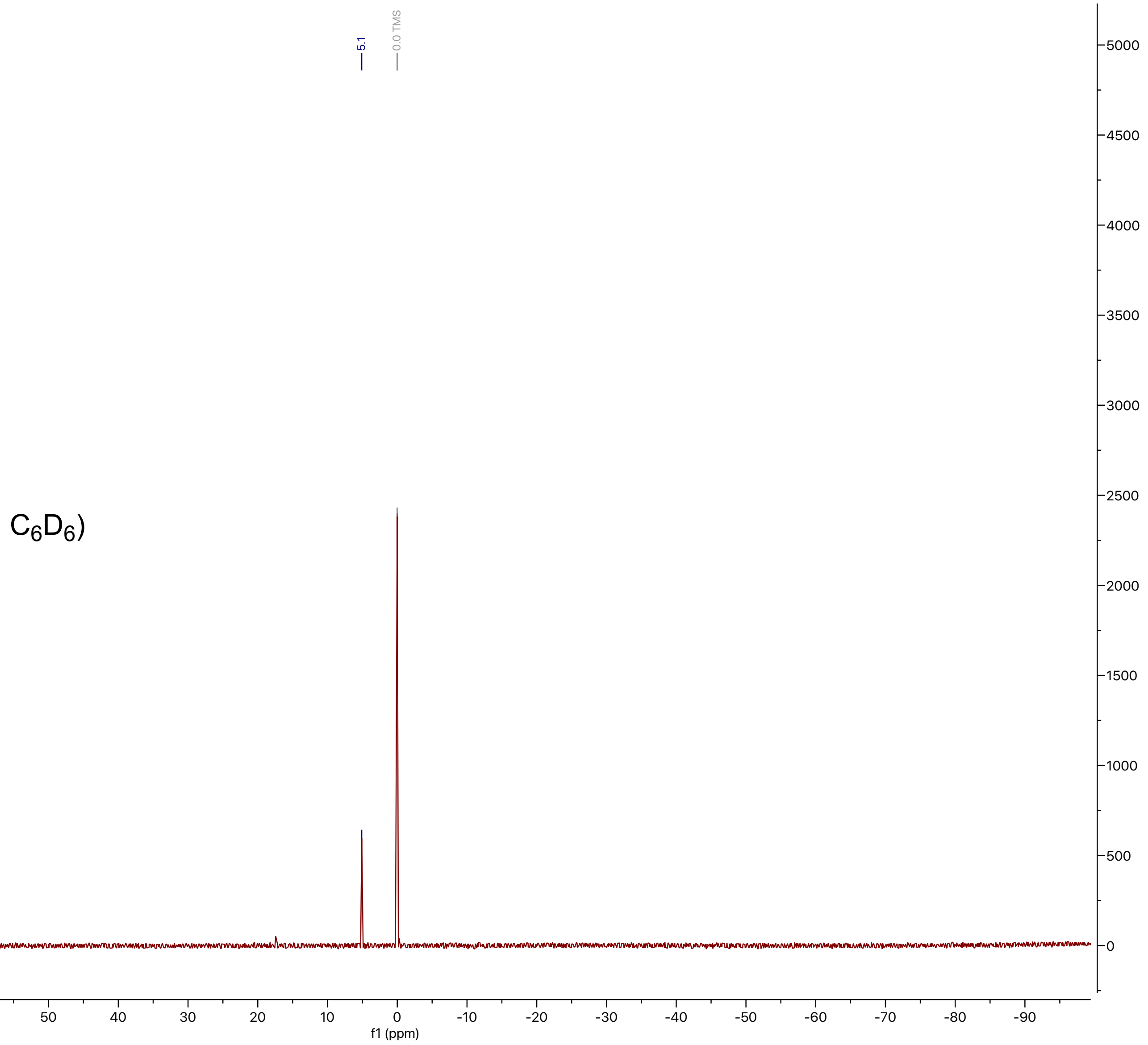

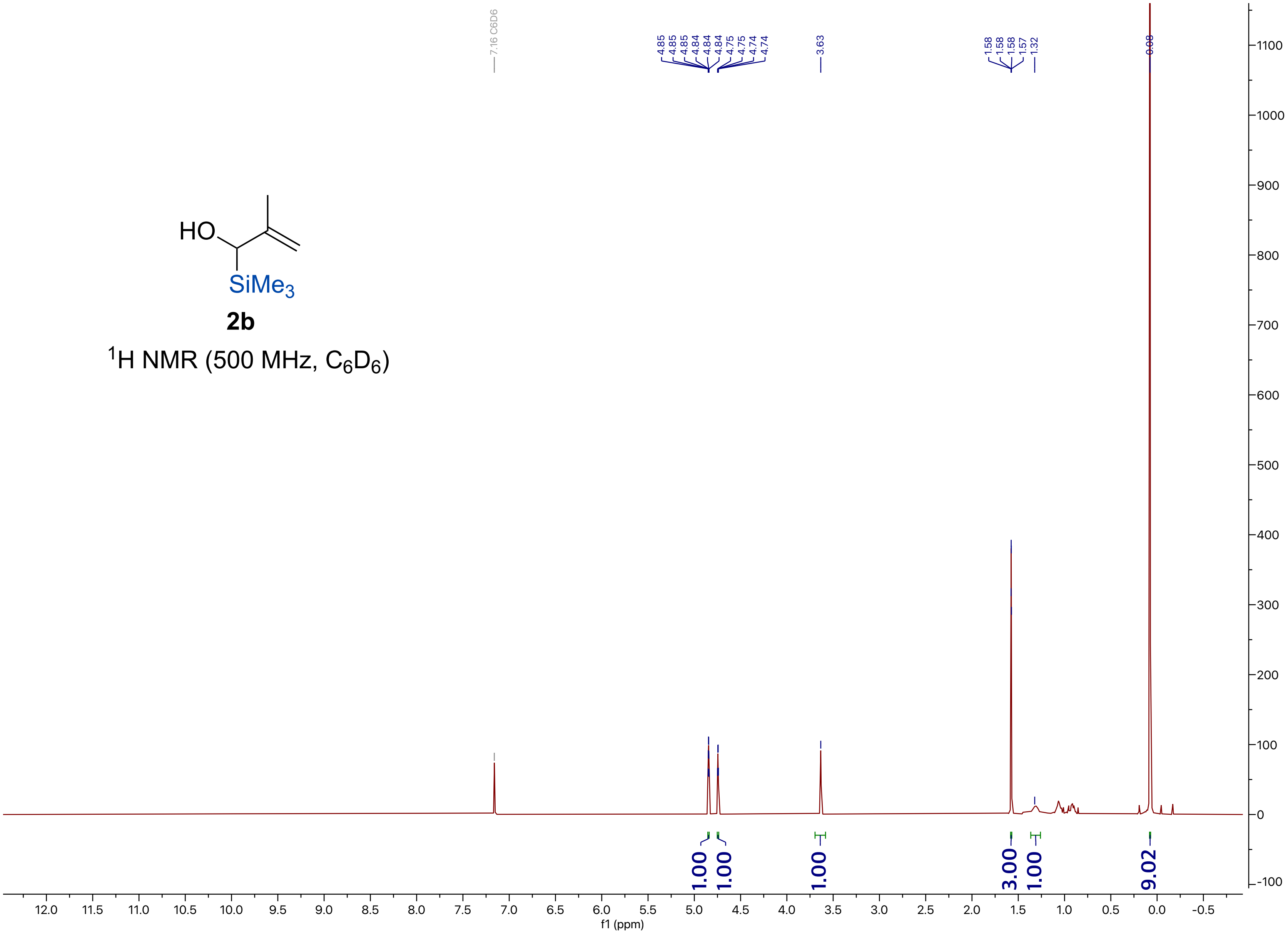
HO
SiMe3
2b
1H NMR (500 MHz, C6D6)
7.16 C6D6
4.85
4.85
4.85
4.84
4.84
4.84
4.75
4.75
4.74
4.74
3.63
1.58
1.58
1.58
1.57
1.32
0.08
1.00
1.00
1.00
3.00
1.00
9.02
f1 (ppm)

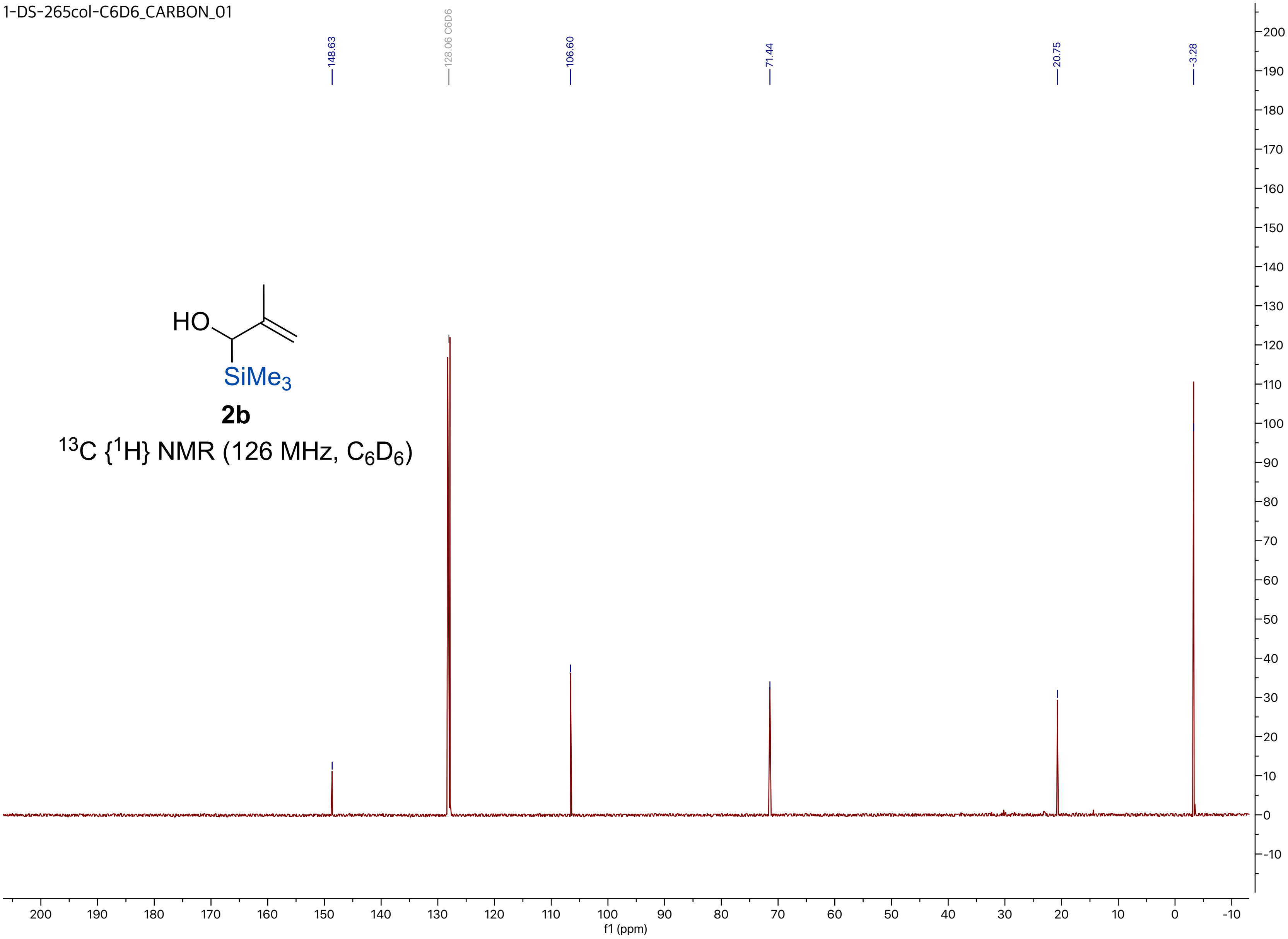
1-DS-265col-C6D6_CARBON_01
148.63
128.06 C6D6
106.60
71.44
20.75
-3.28
HO
SiMe3
2b
$^{13}C\ \{^{1}H\}$ NMR (126 MHz, C_6D_6)
f1 (ppm)

2-DS-28-SM_TMS__s2pul_01

2b

^{29}Si NMR (99 MHz, C_6D_6)

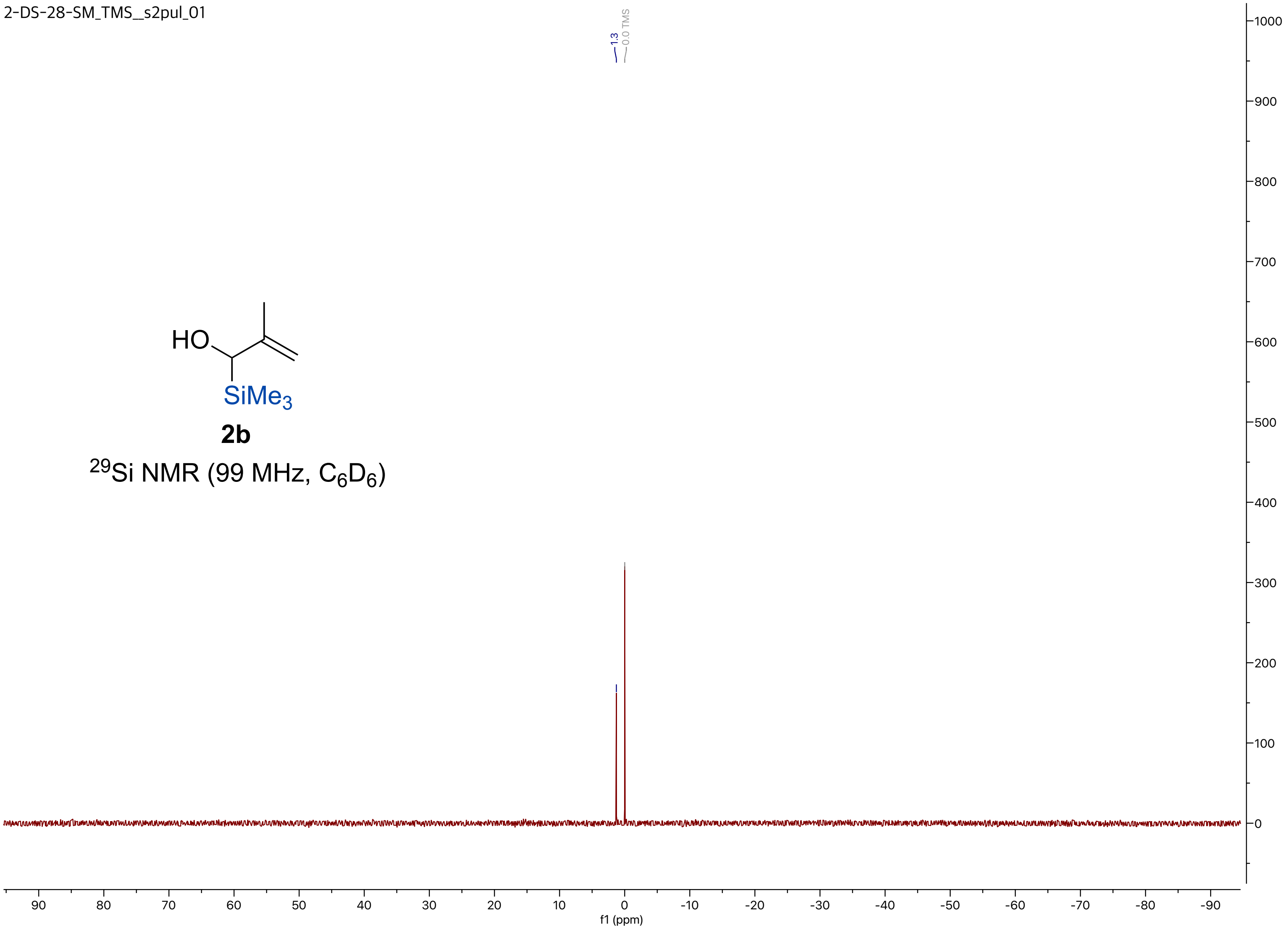

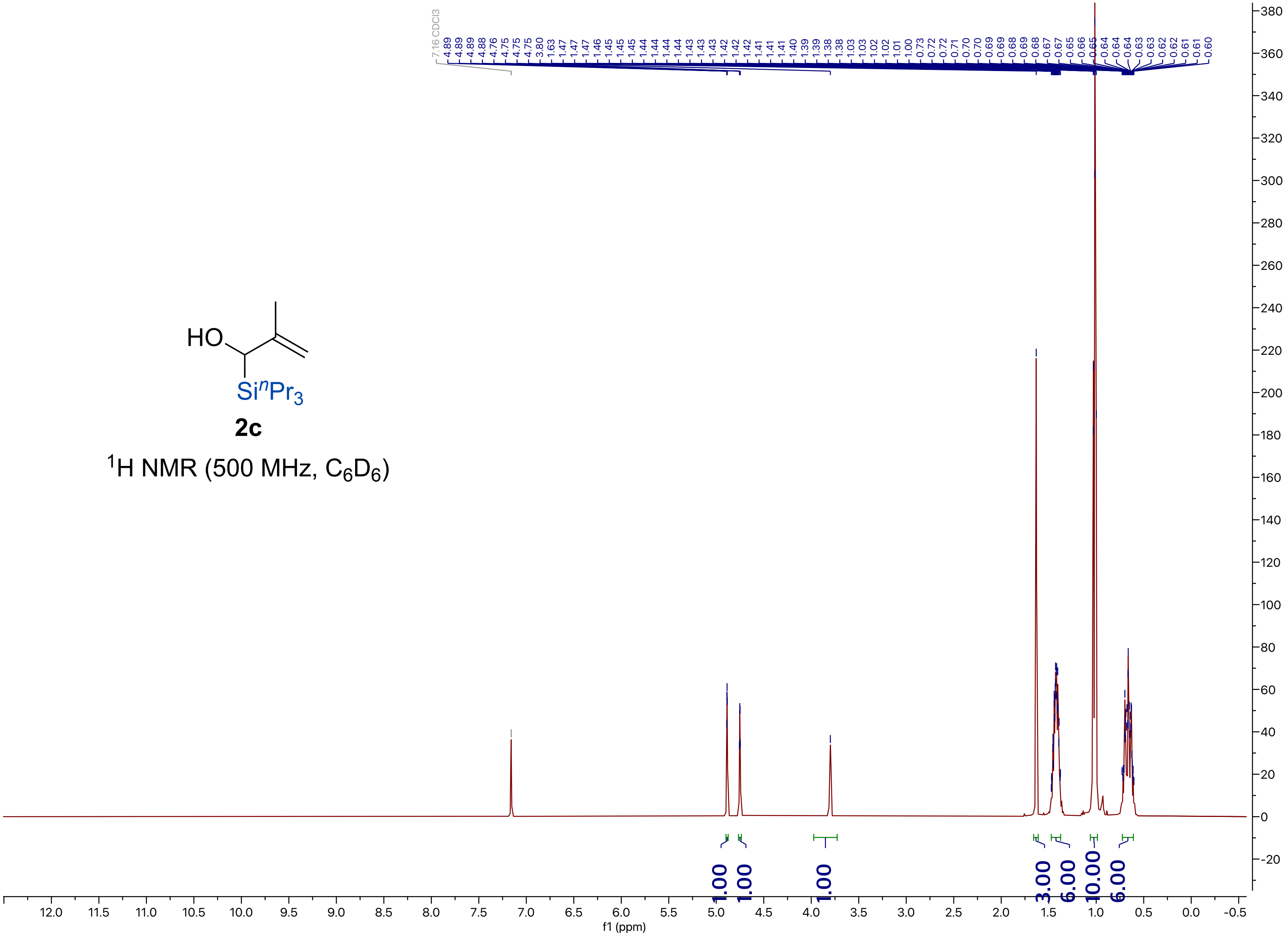

2c

^{1}H NMR (500 MHz, C_6D_6)

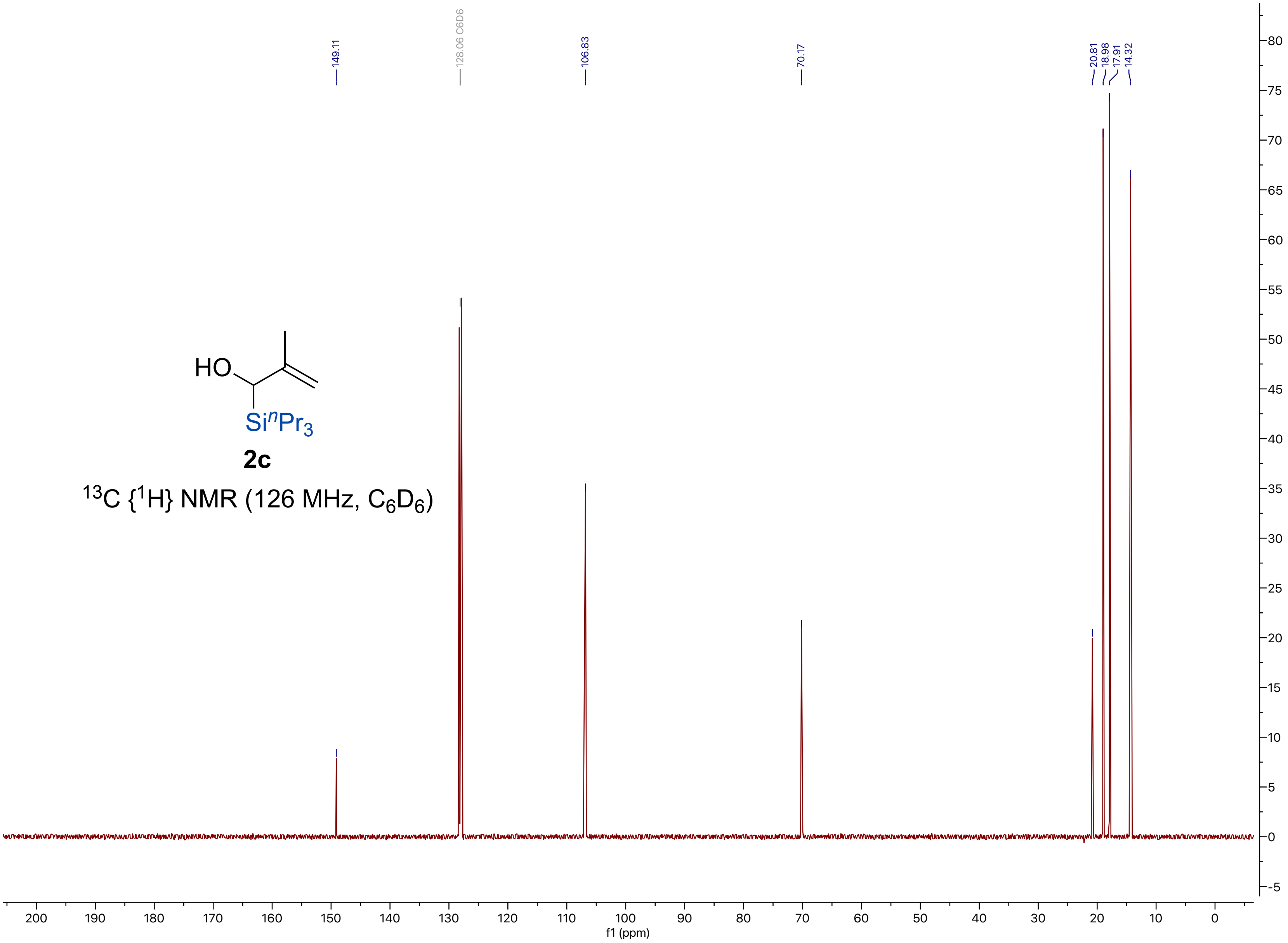

2c

^{13}C {^{1}H} NMR (126 MHz, C_6D_6)

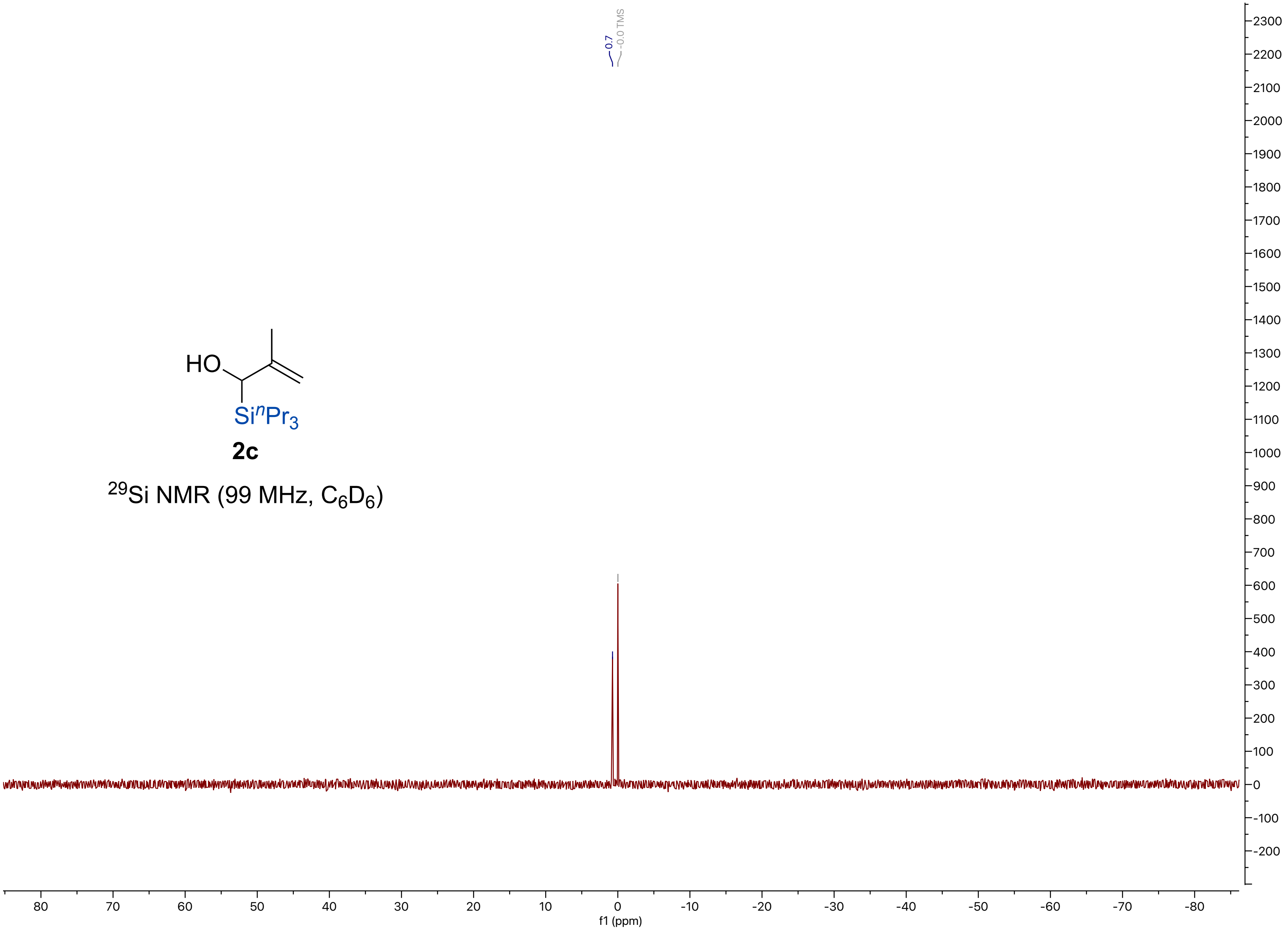

HO
Si^{n}Pr$_3$
2c
^{29}Si NMR (99 MHz, C_6D_6)
0.7
-0.0 TMS
f1 (ppm)

1-DS-290-col14-17_PROTON_01

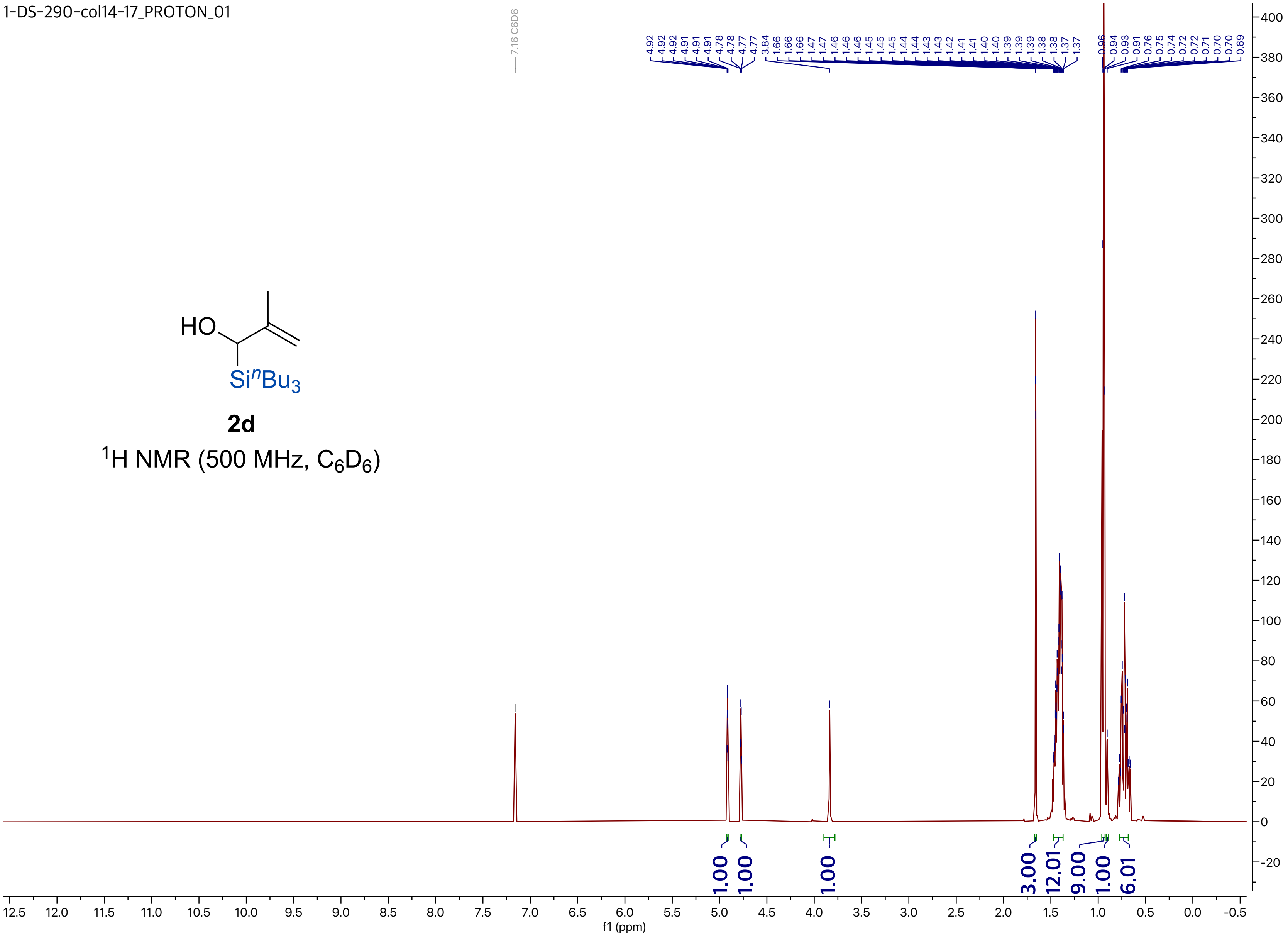

2d

^{1}H NMR (500 MHz, C_6D_6)

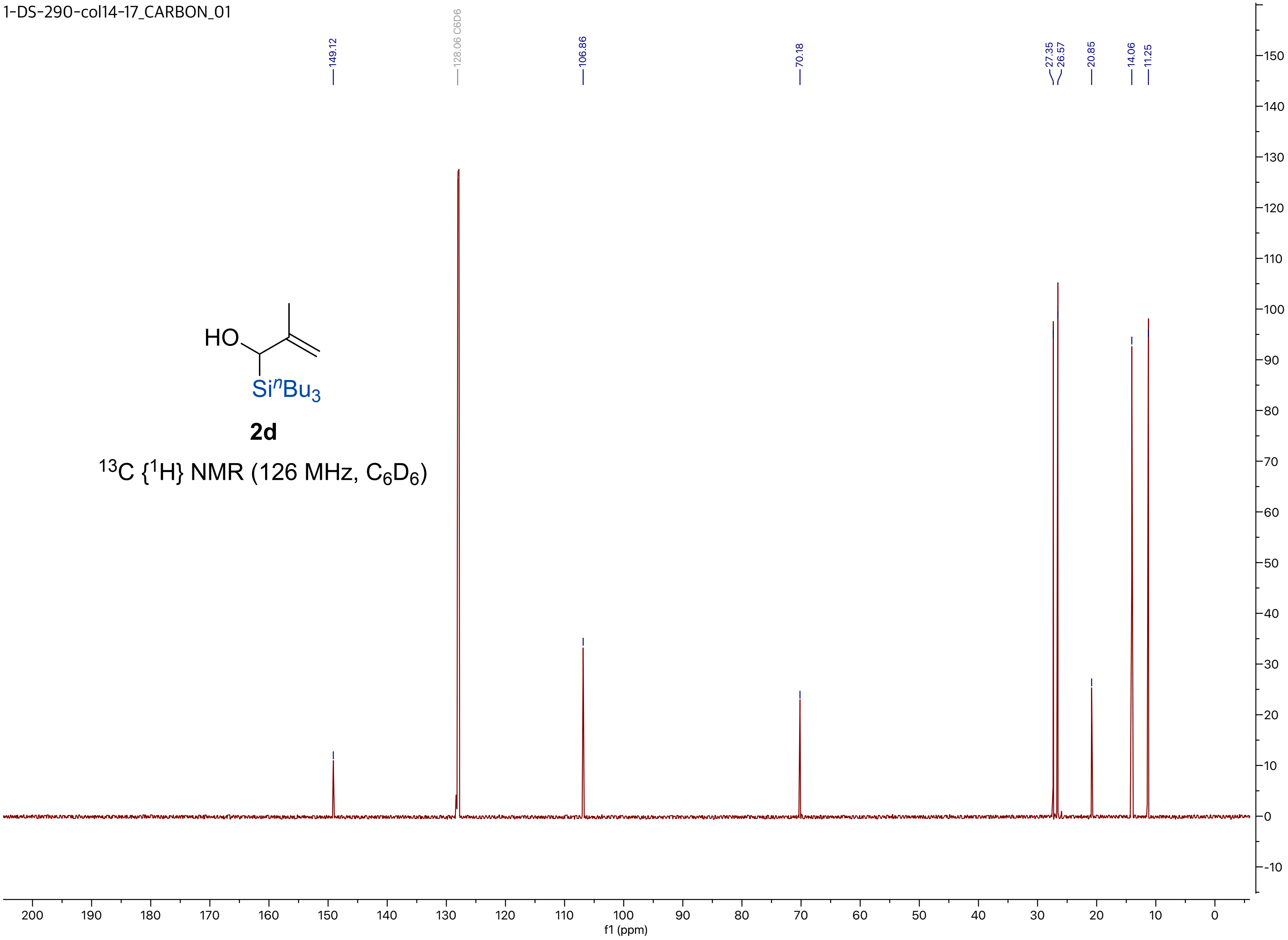
1-DS-290-col14-17_CARBON_01
149.12
128.06 C6D6
106.86
70.18
27.35
26.57
20.85
14.06
11.25
HO
Si^{n}Bu$_3$
2d
^{13}C {^{1}H} NMR (126 MHz, C_6D_6)
f1 (ppm)

1-DS-290-TMS_s2pul_01

2d

^{29}Si NMR (99 MHz, C_6D_6)

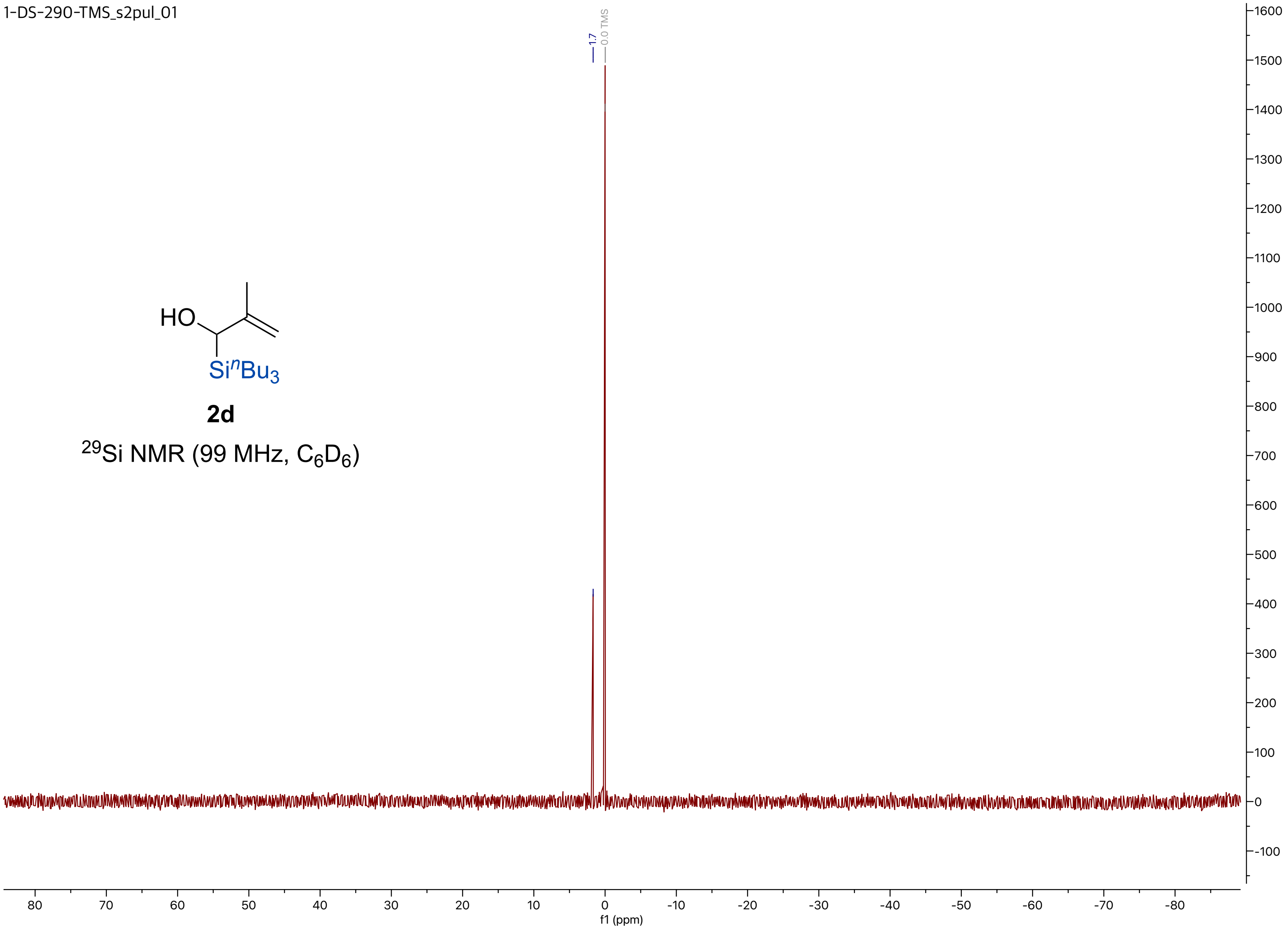

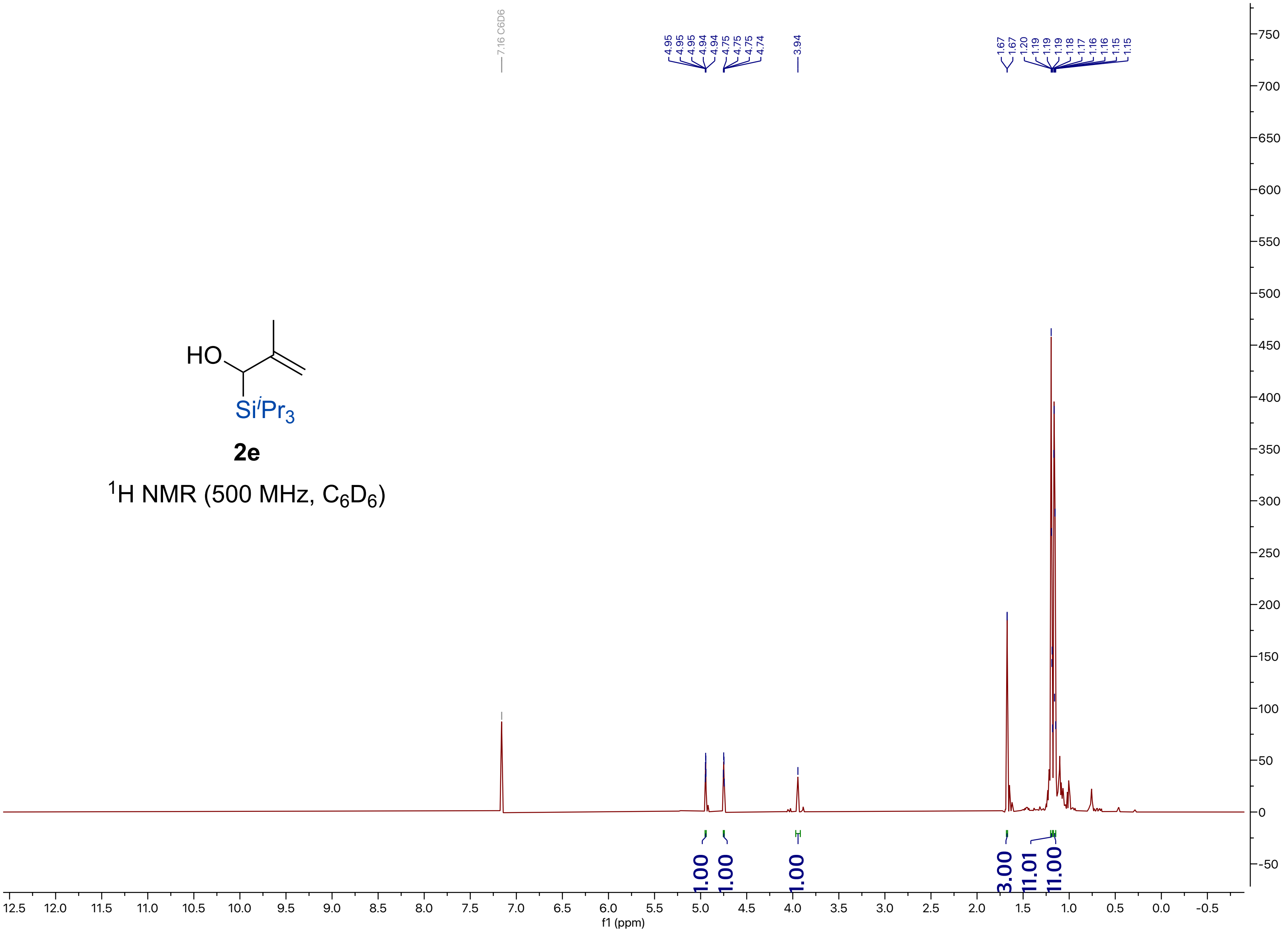

2e

^{1}H NMR (500 MHz, C_6D_6)

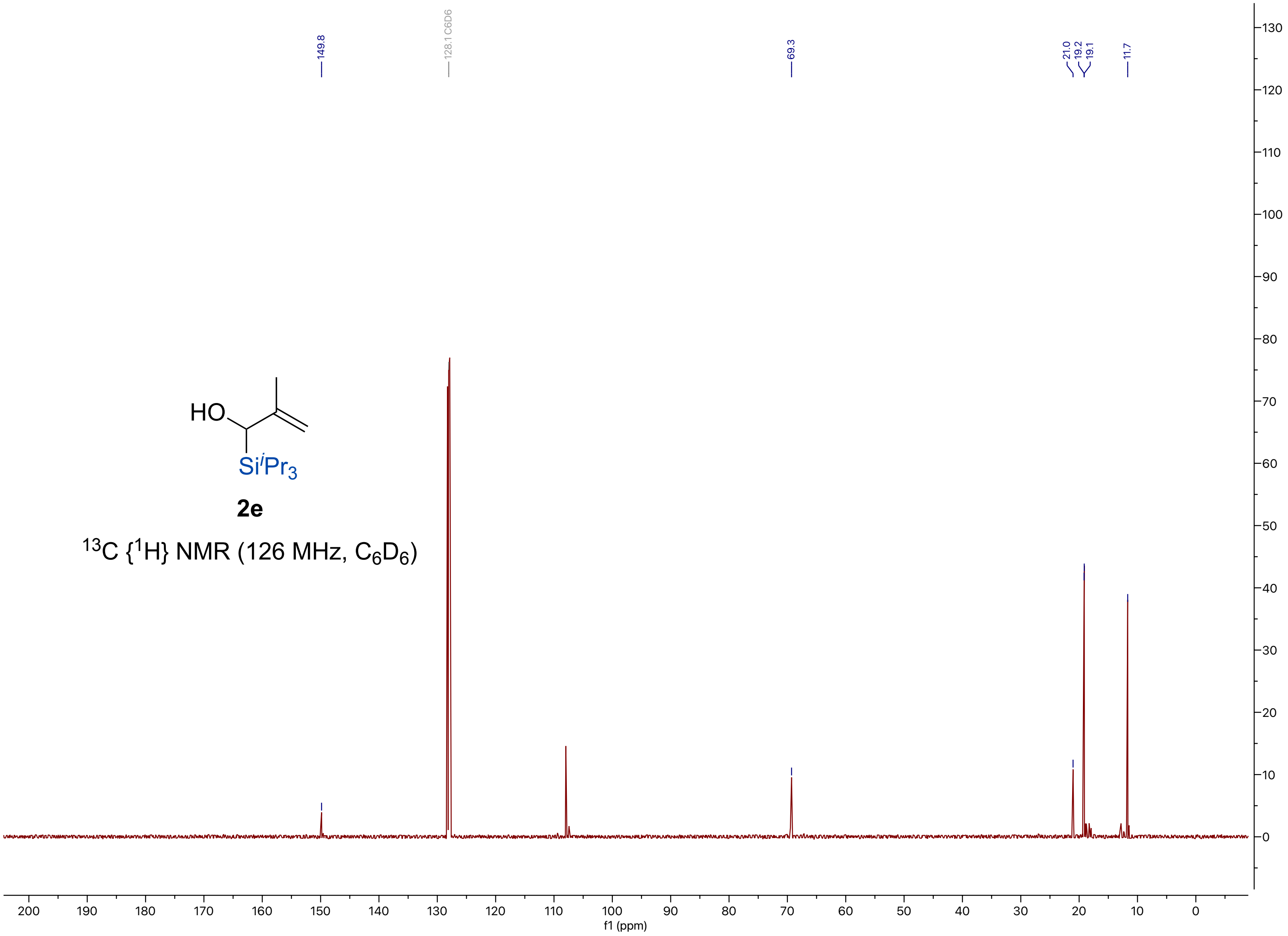

2e

^{13}C {^{1}H} NMR (126 MHz, C_6D_6)

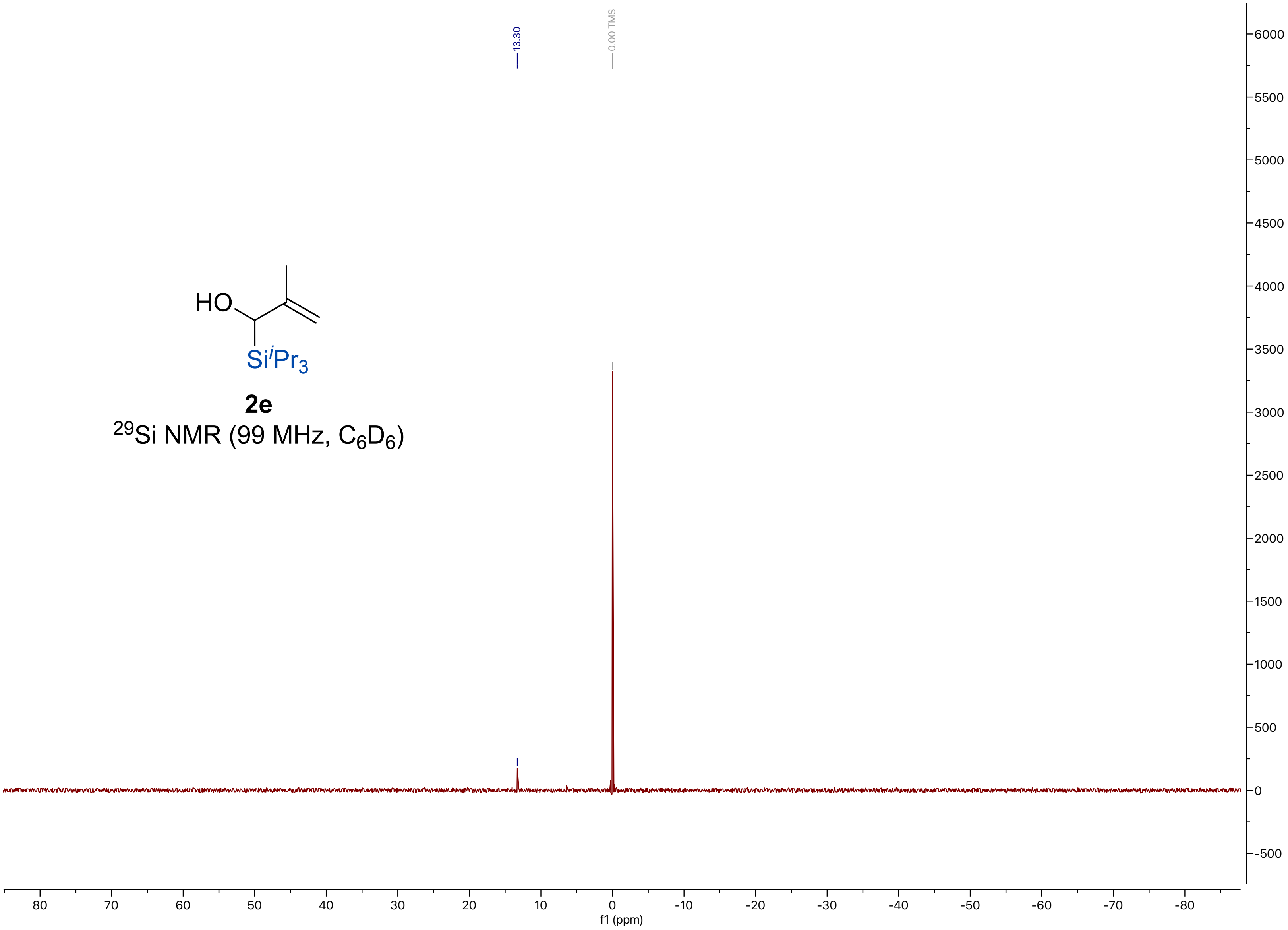

HO
Si^{i}Pr$_3$
2e
^{29}Si NMR (99 MHz, C_6D_6)
13.30
0.00 TMS
f1 (ppm)

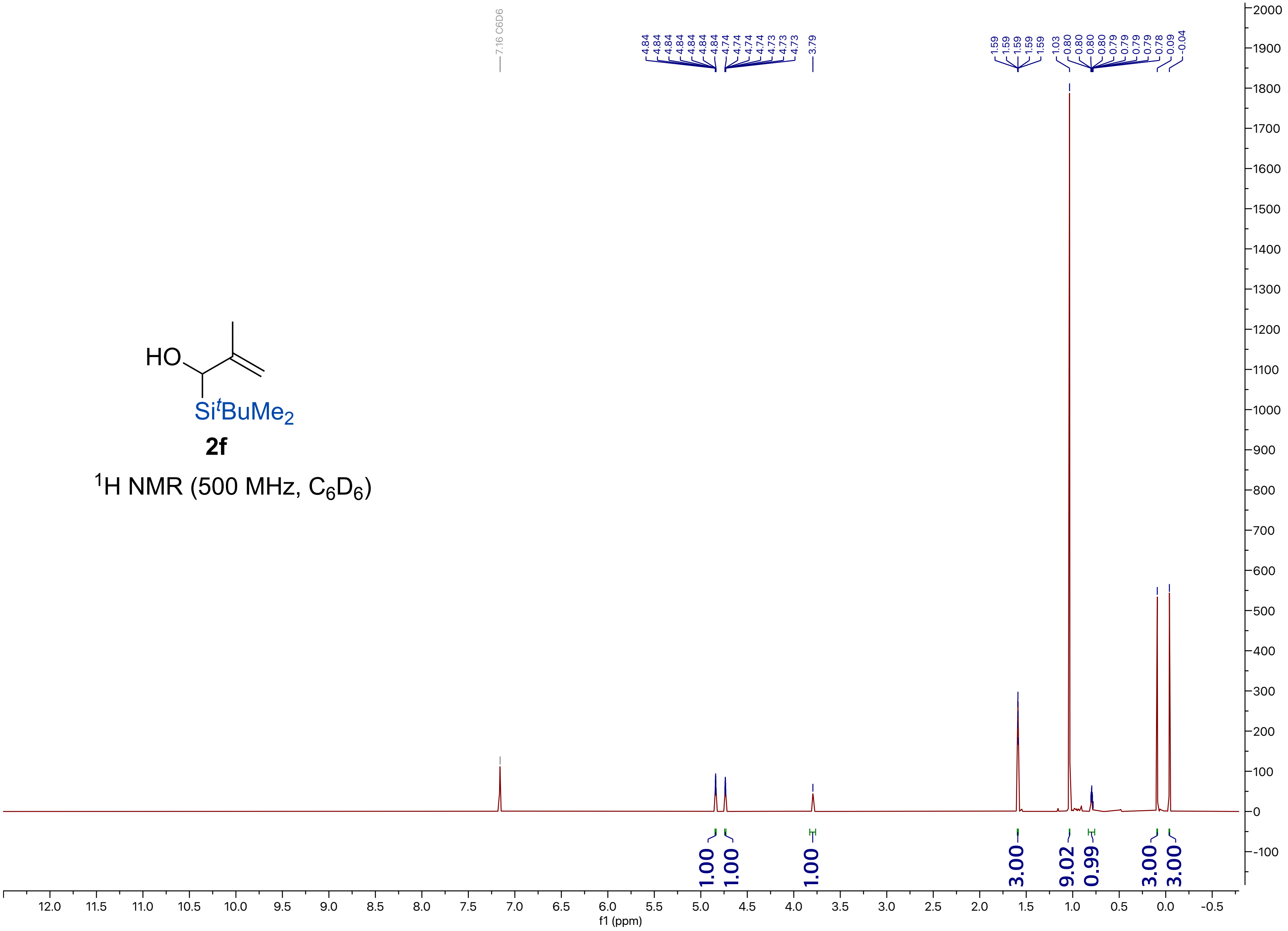

2f

^1H NMR (500 MHz, C_6D_6)

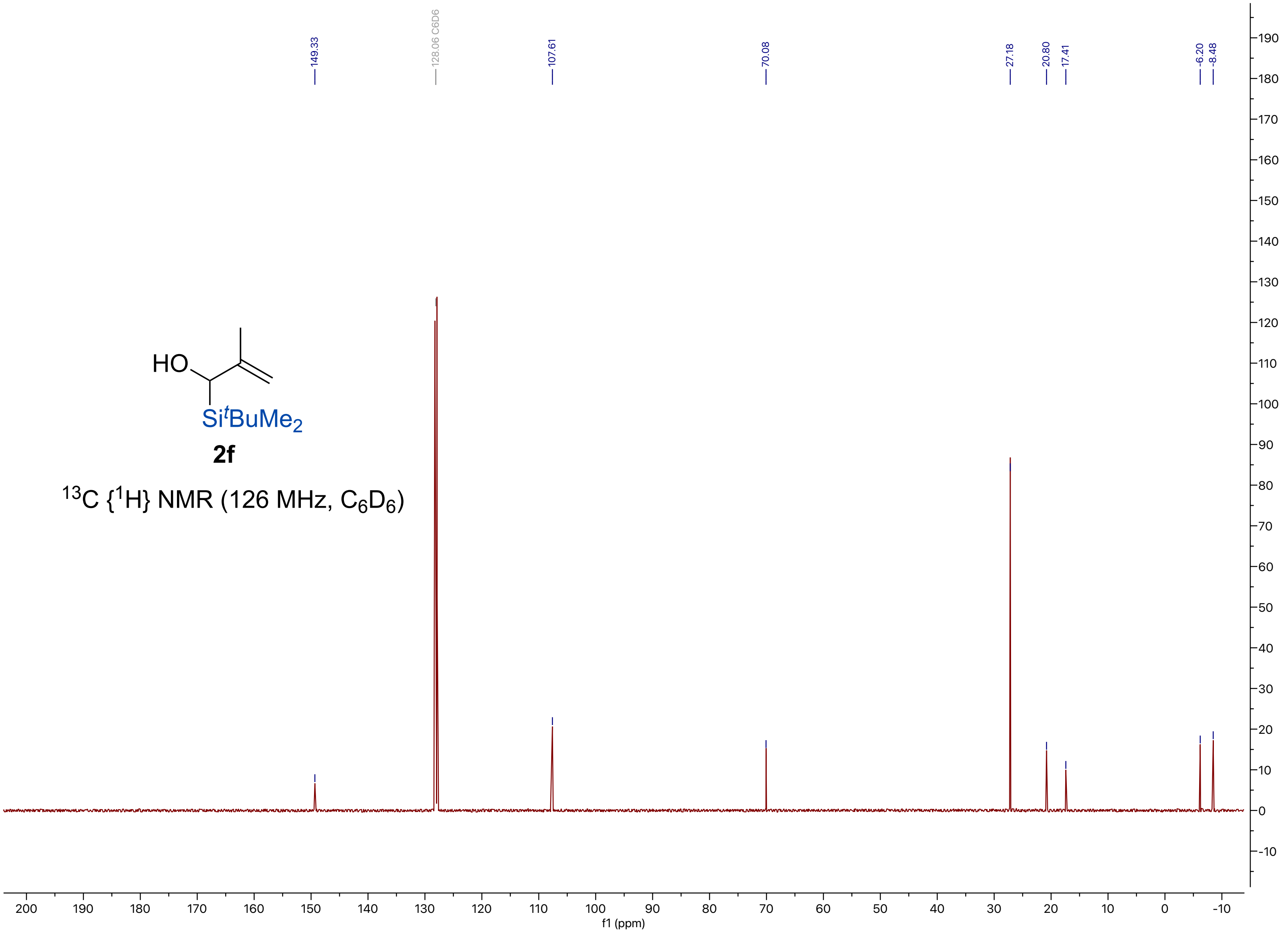

2f

^{13}C {^1H} NMR (126 MHz, C_6D_6)

2-DS-43SM_TMS_s2pul_01

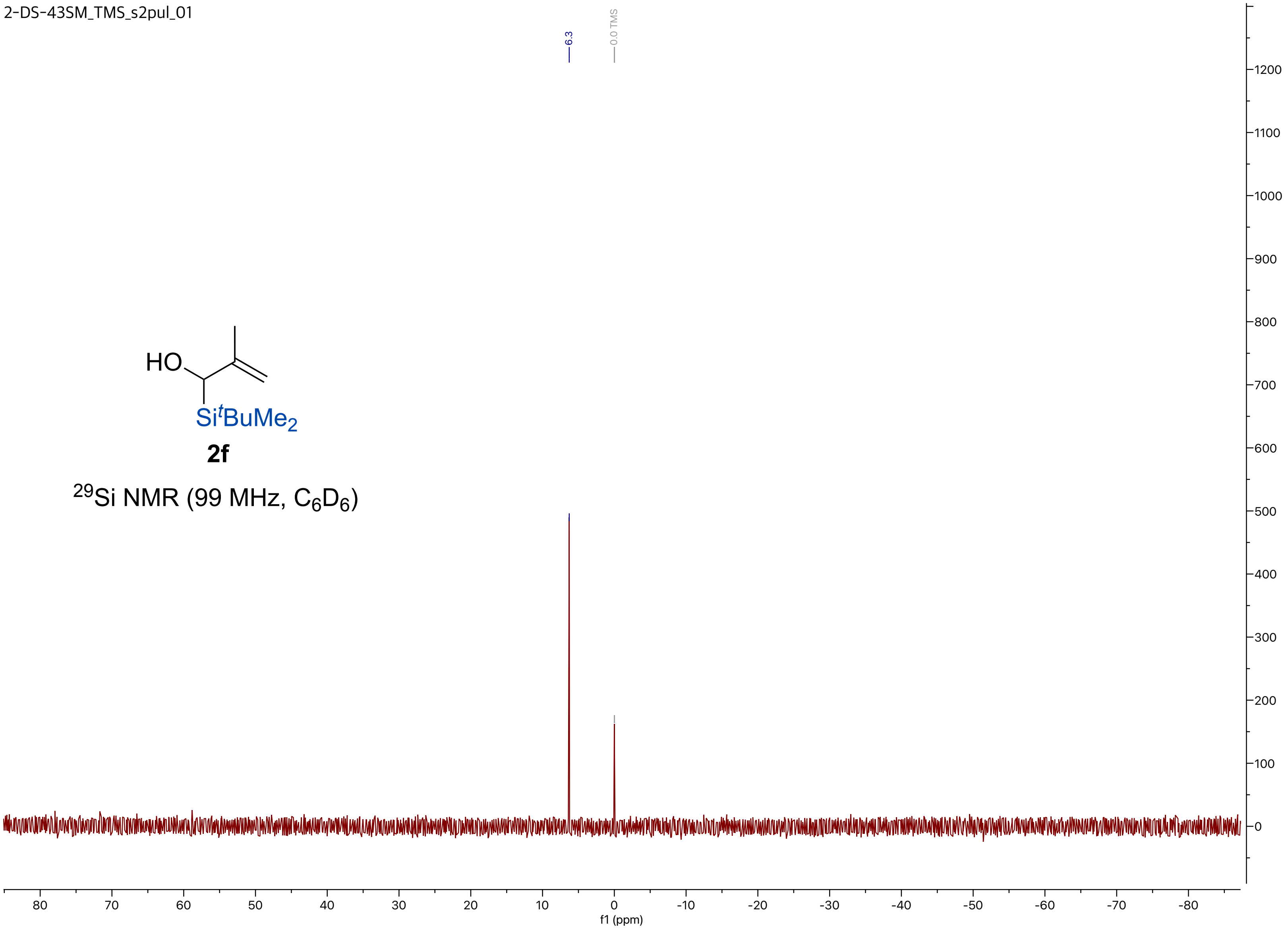

2f

^{29}Si NMR (99 MHz, C_6D_6)

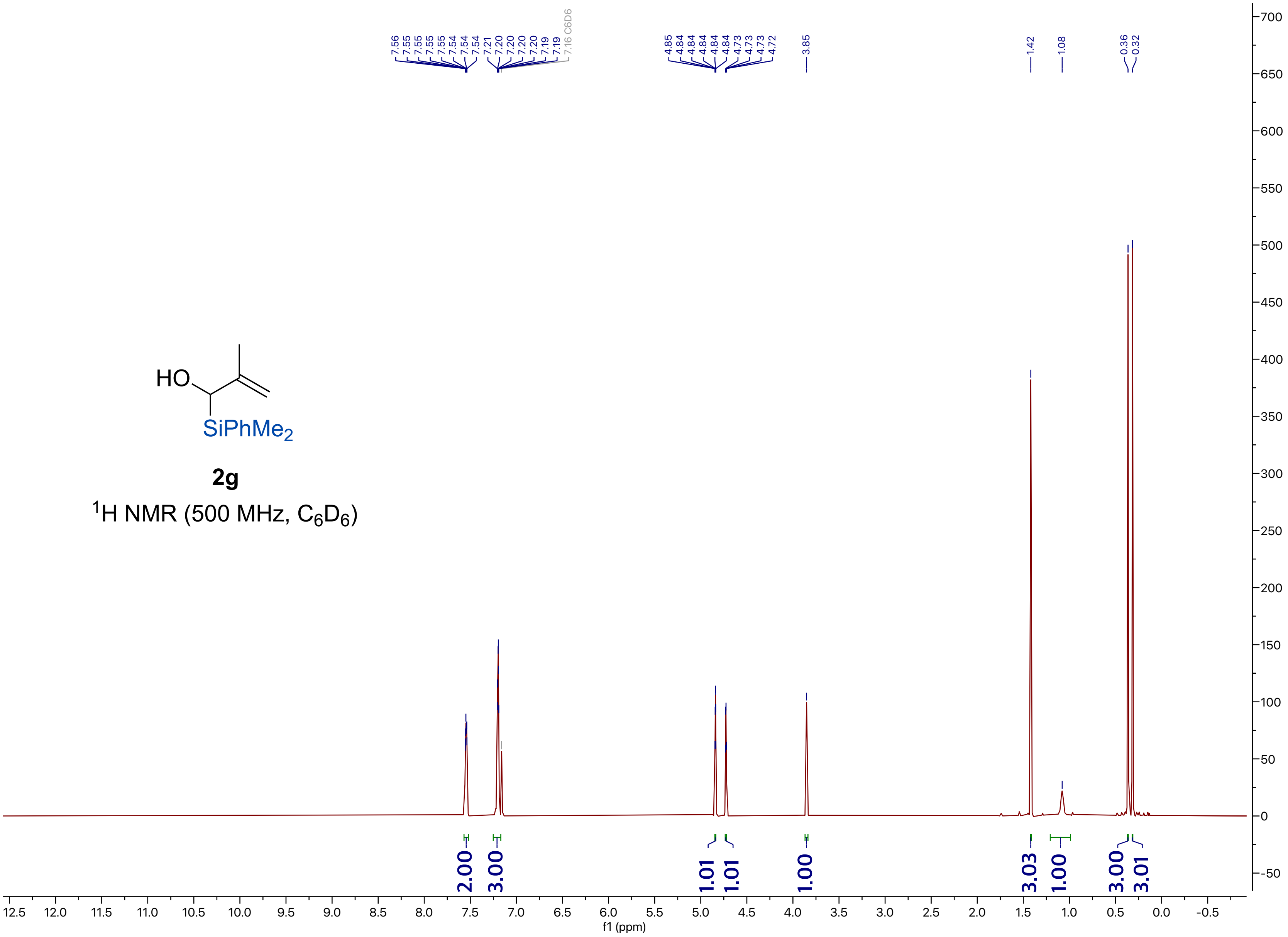

2g

^{1}H NMR (500 MHz, C_6D_6)

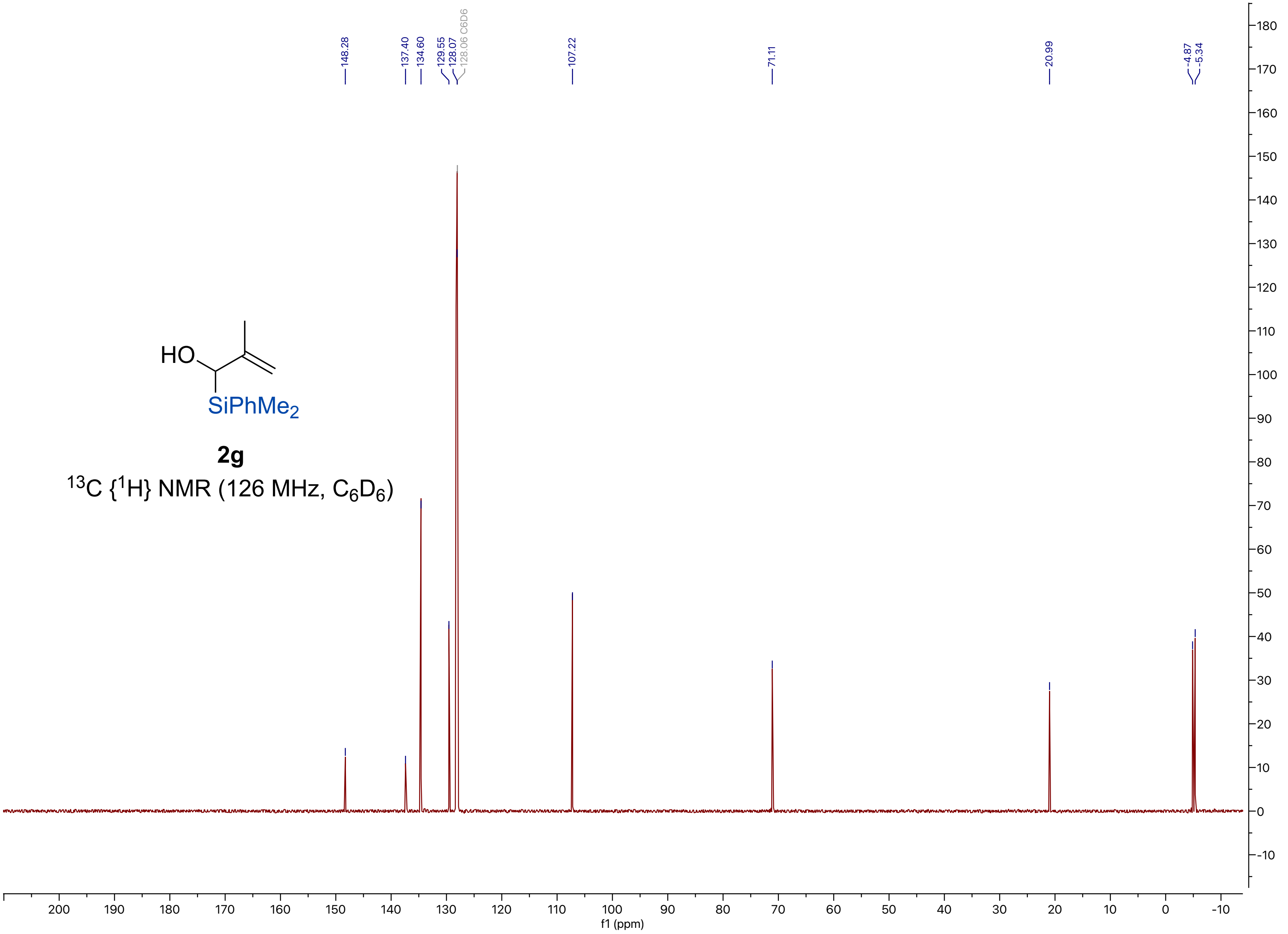

148.28
137.40
134.60
129.55
128.07
128.06 C6D6
107.22
71.11
20.99
-4.87
-5.34
SiPhMe2
HO
2g
13C {1H} NMR (126 MHz, C6D6)
f1 (ppm)

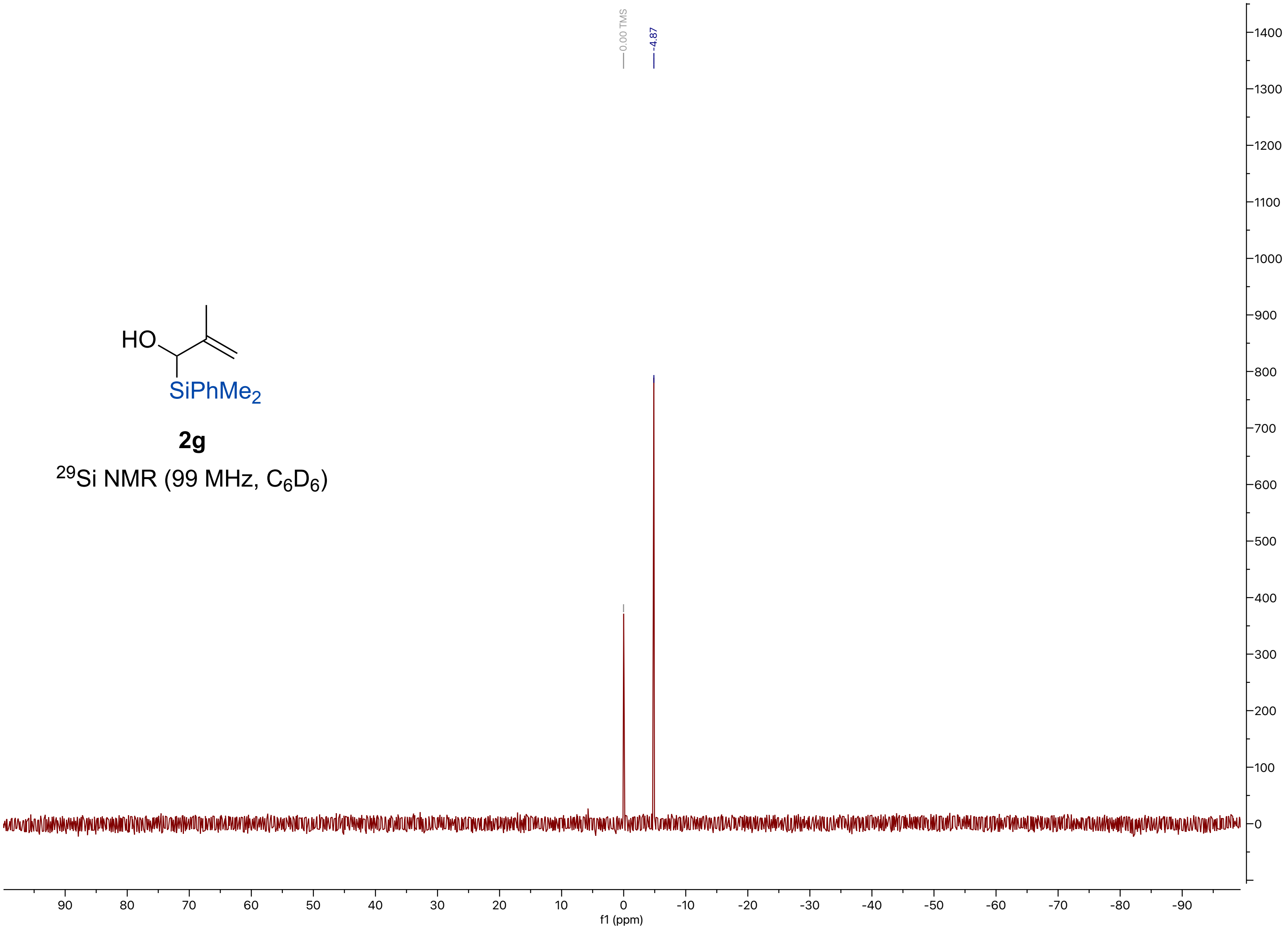

2g

^{29}Si NMR (99 MHz, C_6D_6)

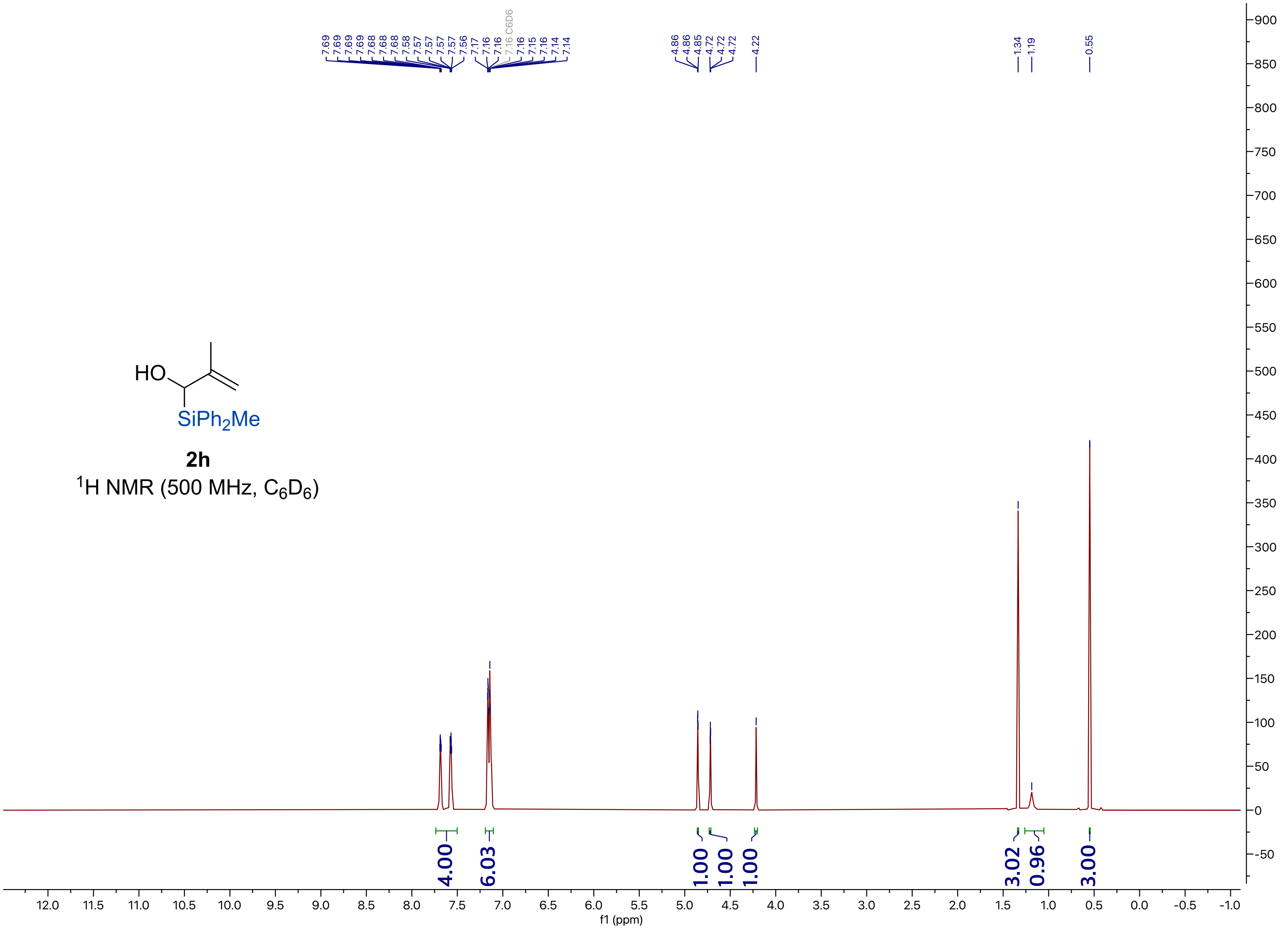

HO
SiPh2Me
2h
1H NMR (500 MHz, C6D6)
f1 (ppm)
4.00
6.03
1.00
1.00
1.00
3.02
0.96
3.00

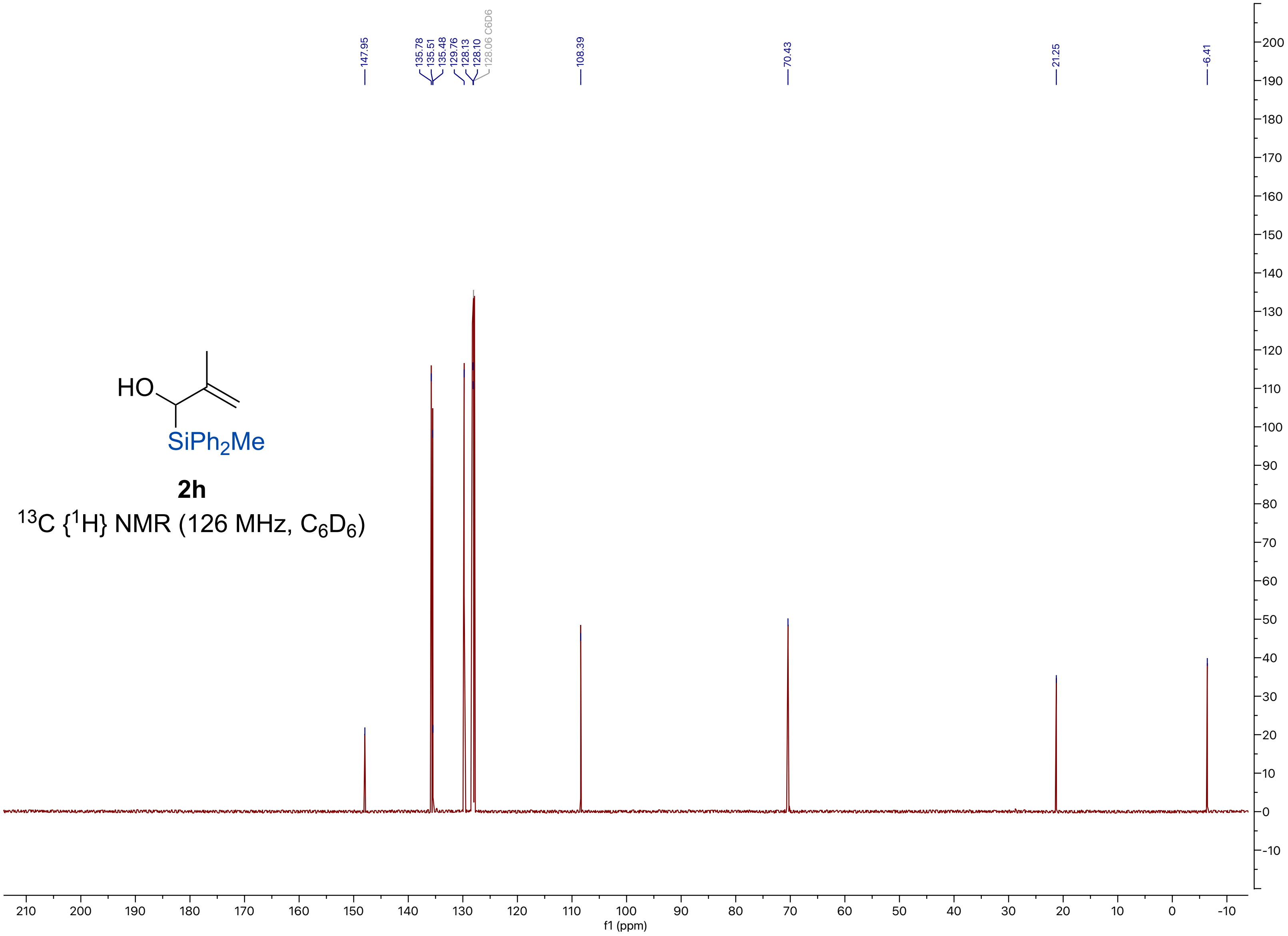

2h

$^{13}C\ \{^{1}H\}$ NMR (126 MHz, C_6D_6)

2-DS-17-SM_TMS_s2pul_01

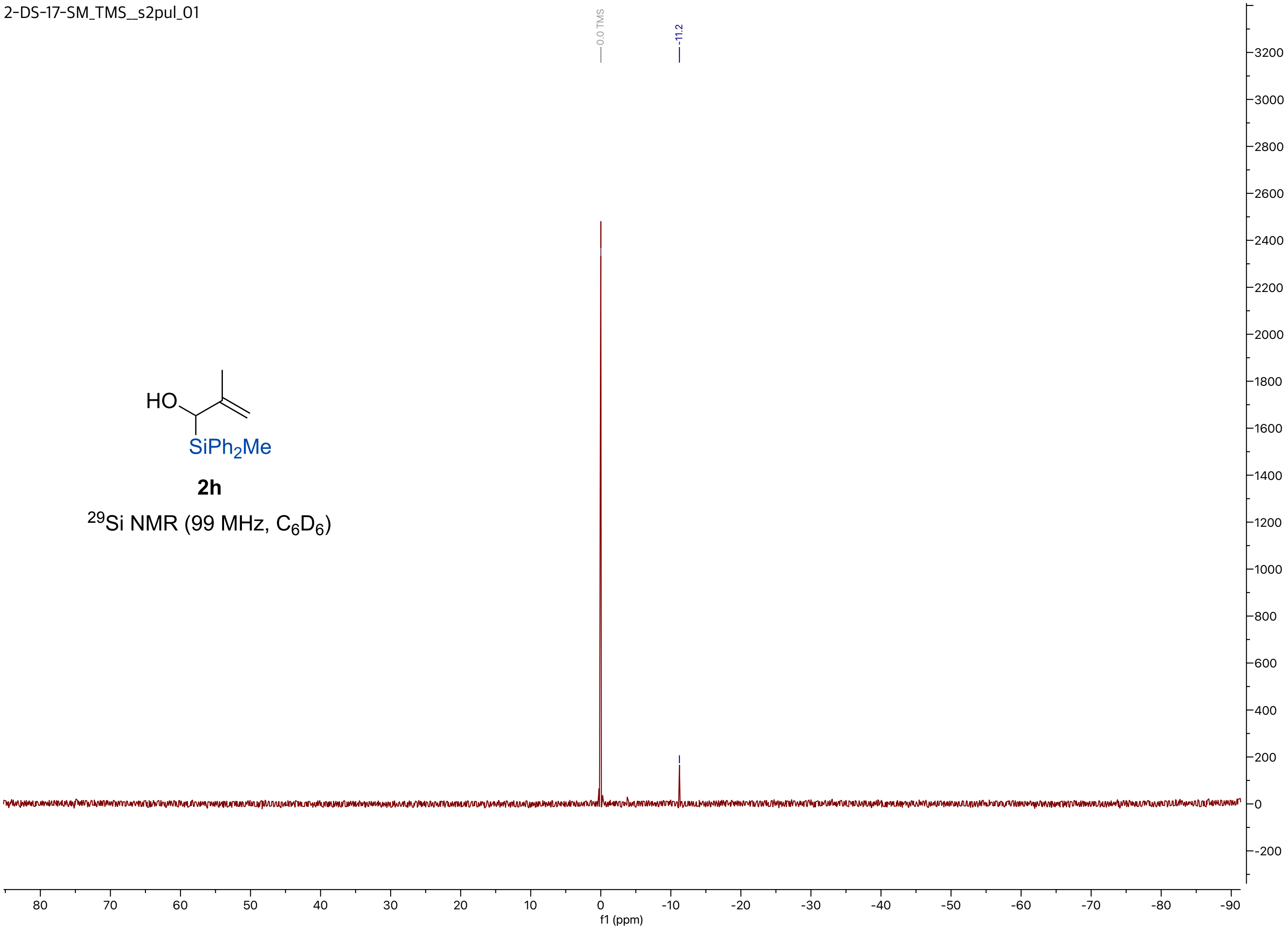

2h

^{29}Si NMR (99 MHz, C_6D_6)

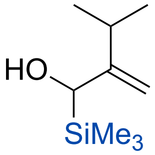

2i

^{1}H NMR (500 MHz, C_6D_6)

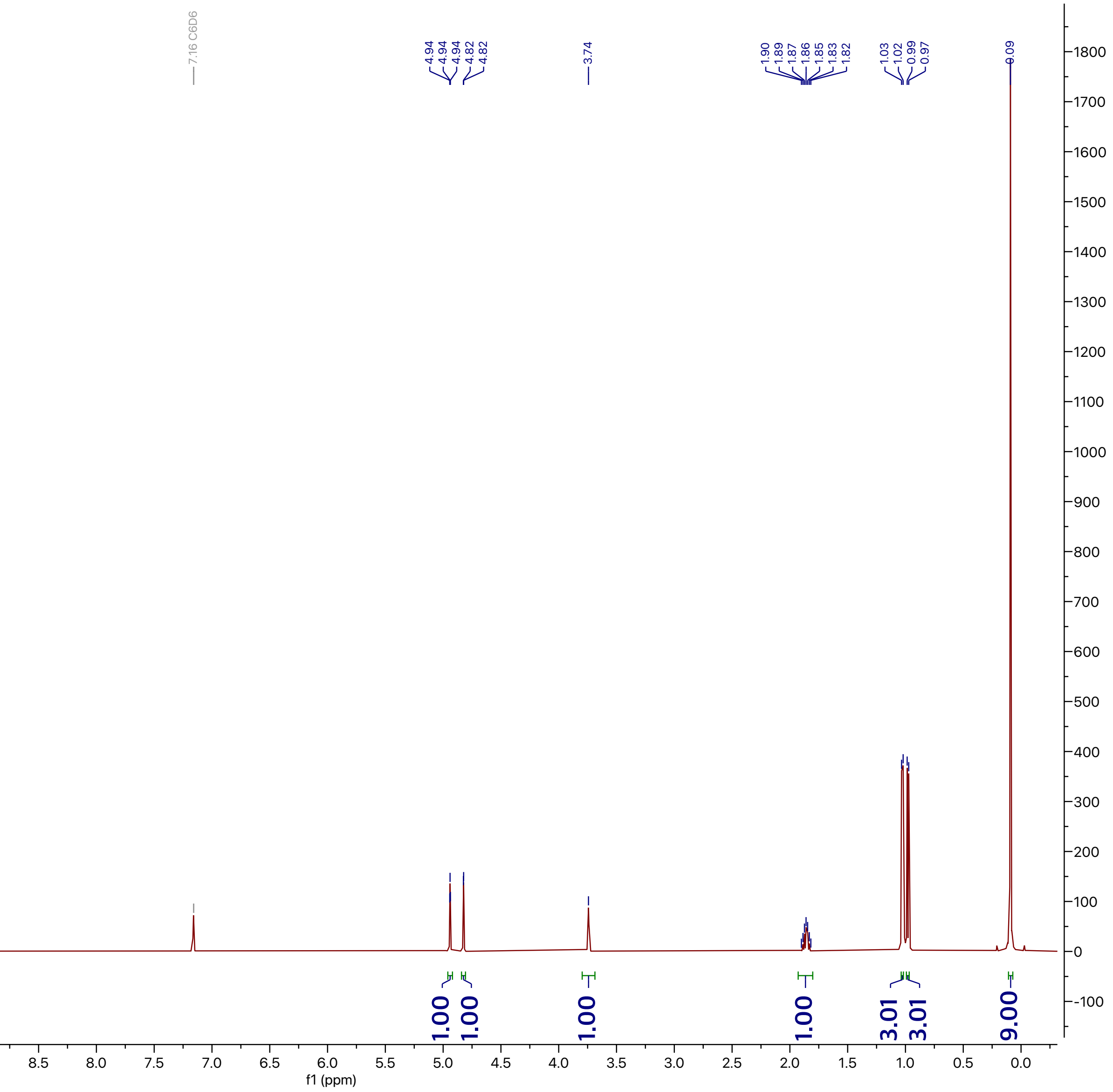

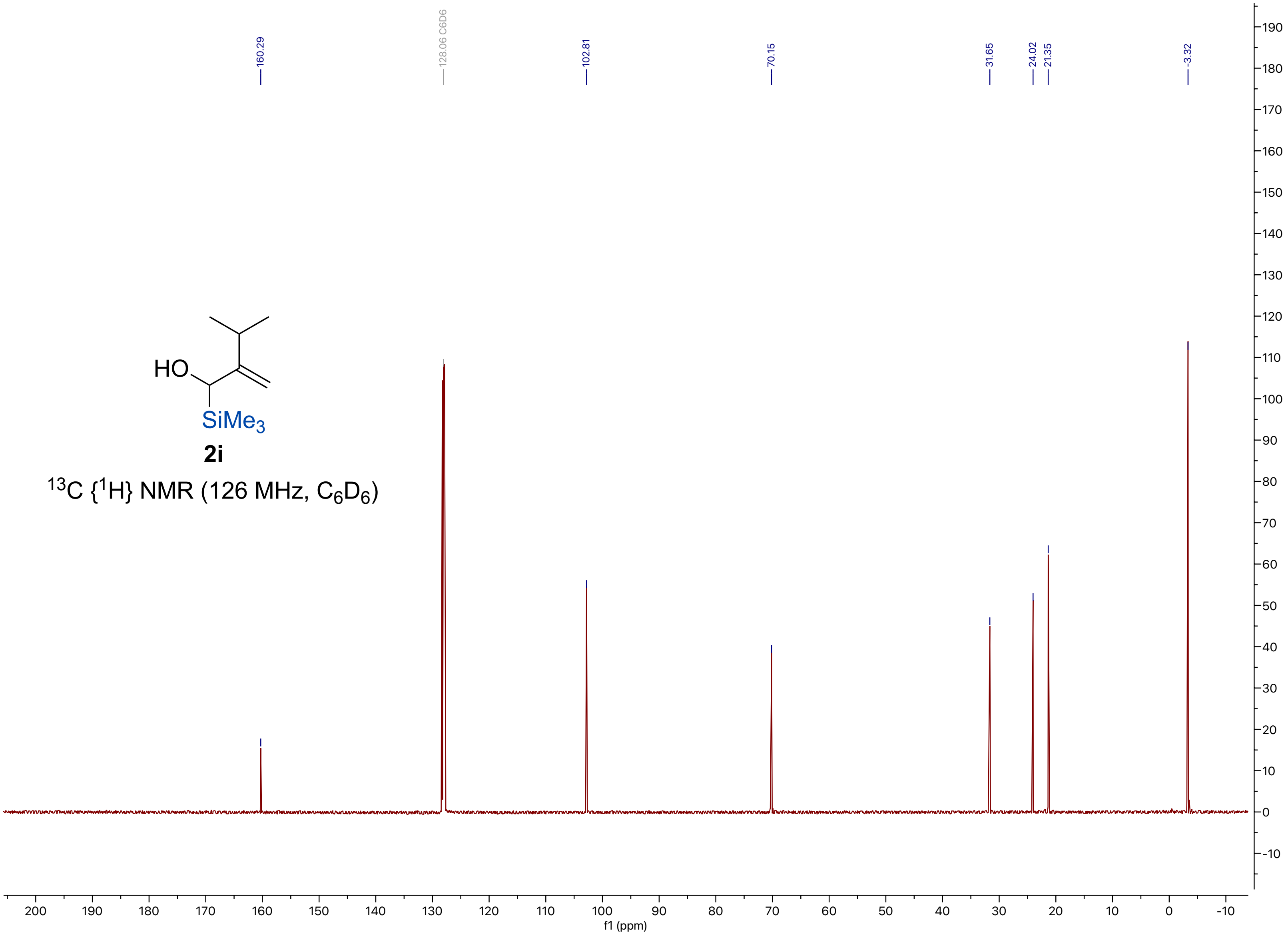

2i

$^{13}C\ \{^{1}H\}$ NMR (126 MHz, C_6D_6)

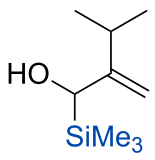

2i

^{29}Si NMR (99 MHz, C_6D_6)

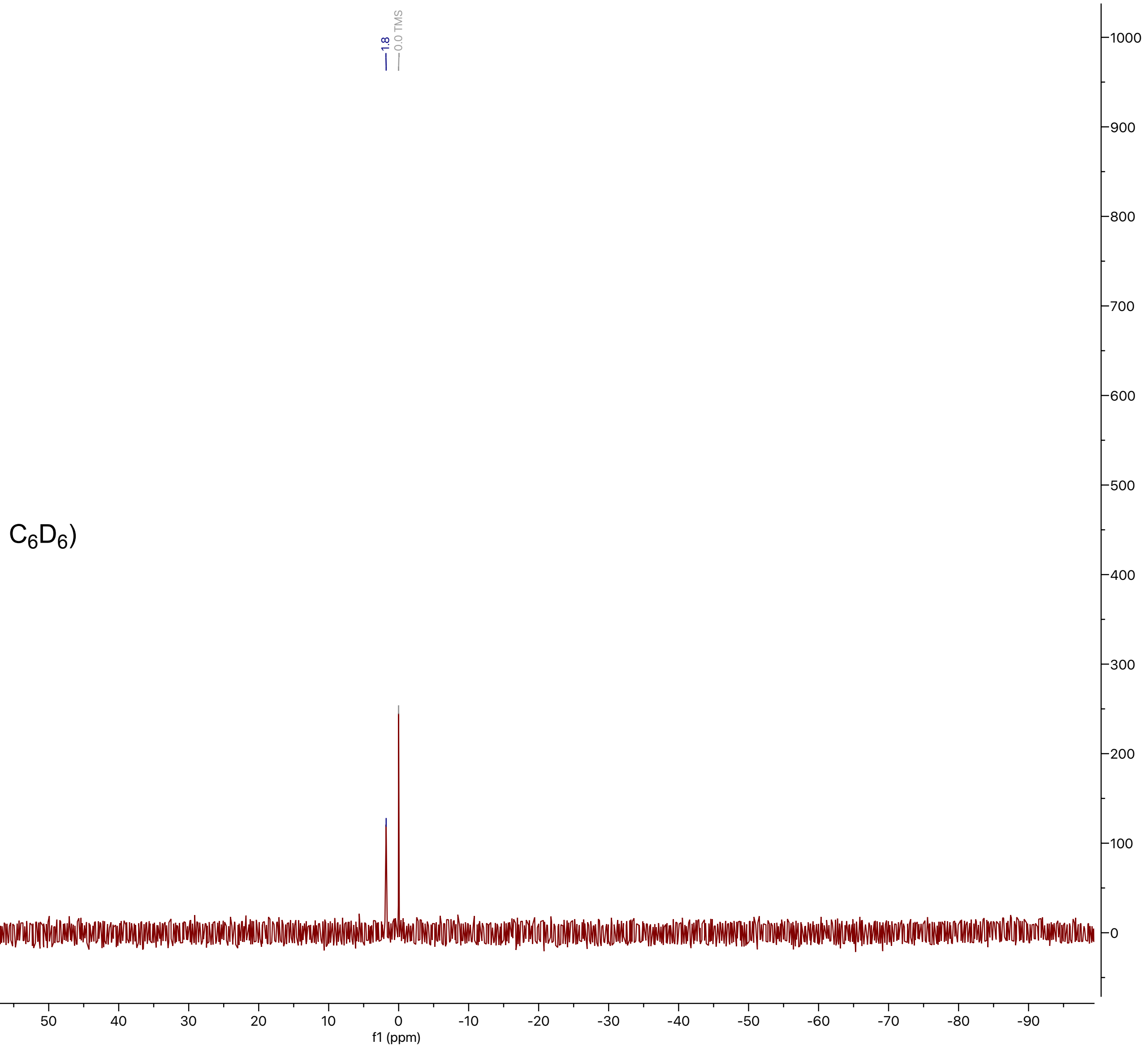

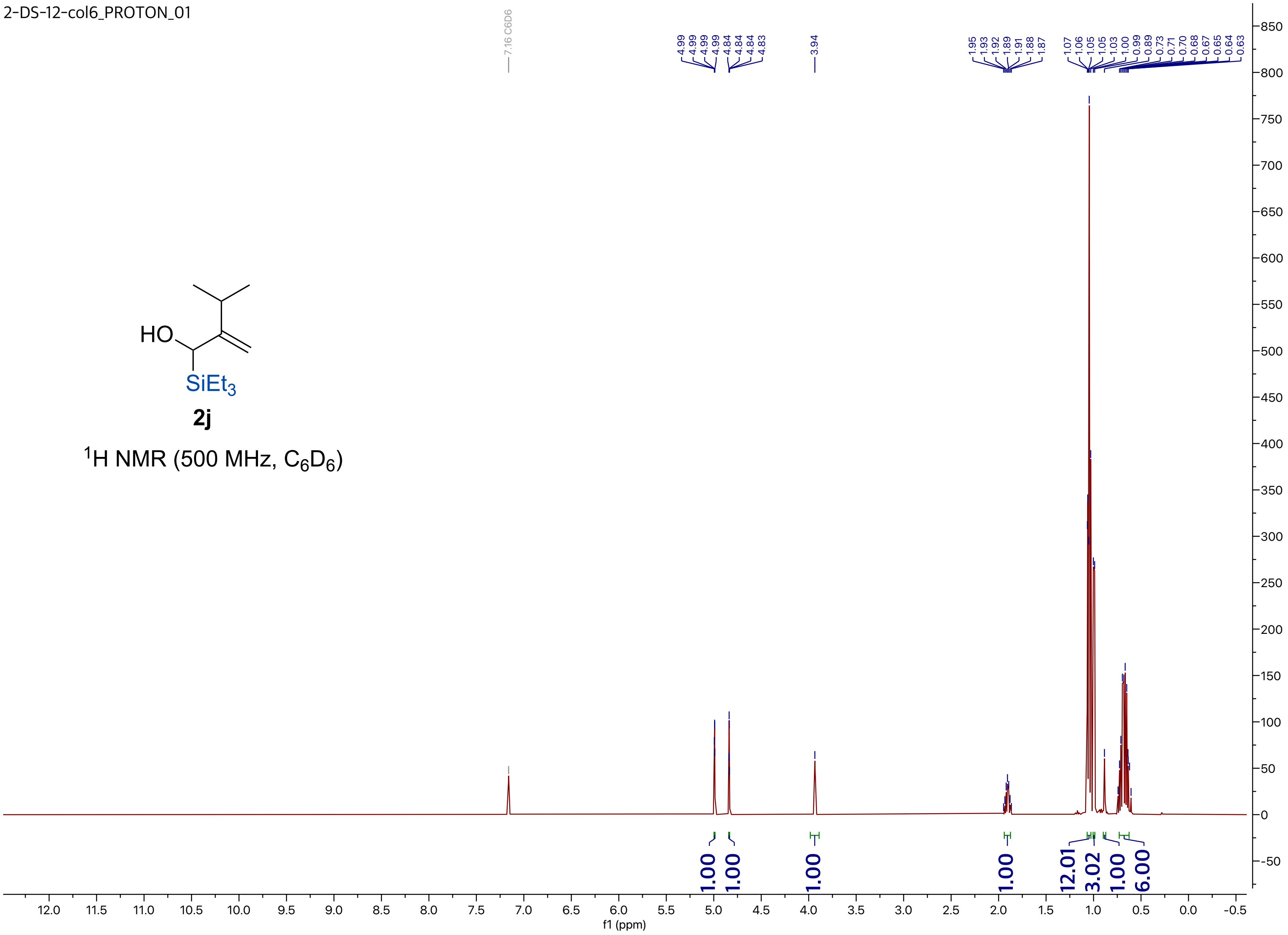

2j

^1H NMR (500 MHz, C_6D_6)

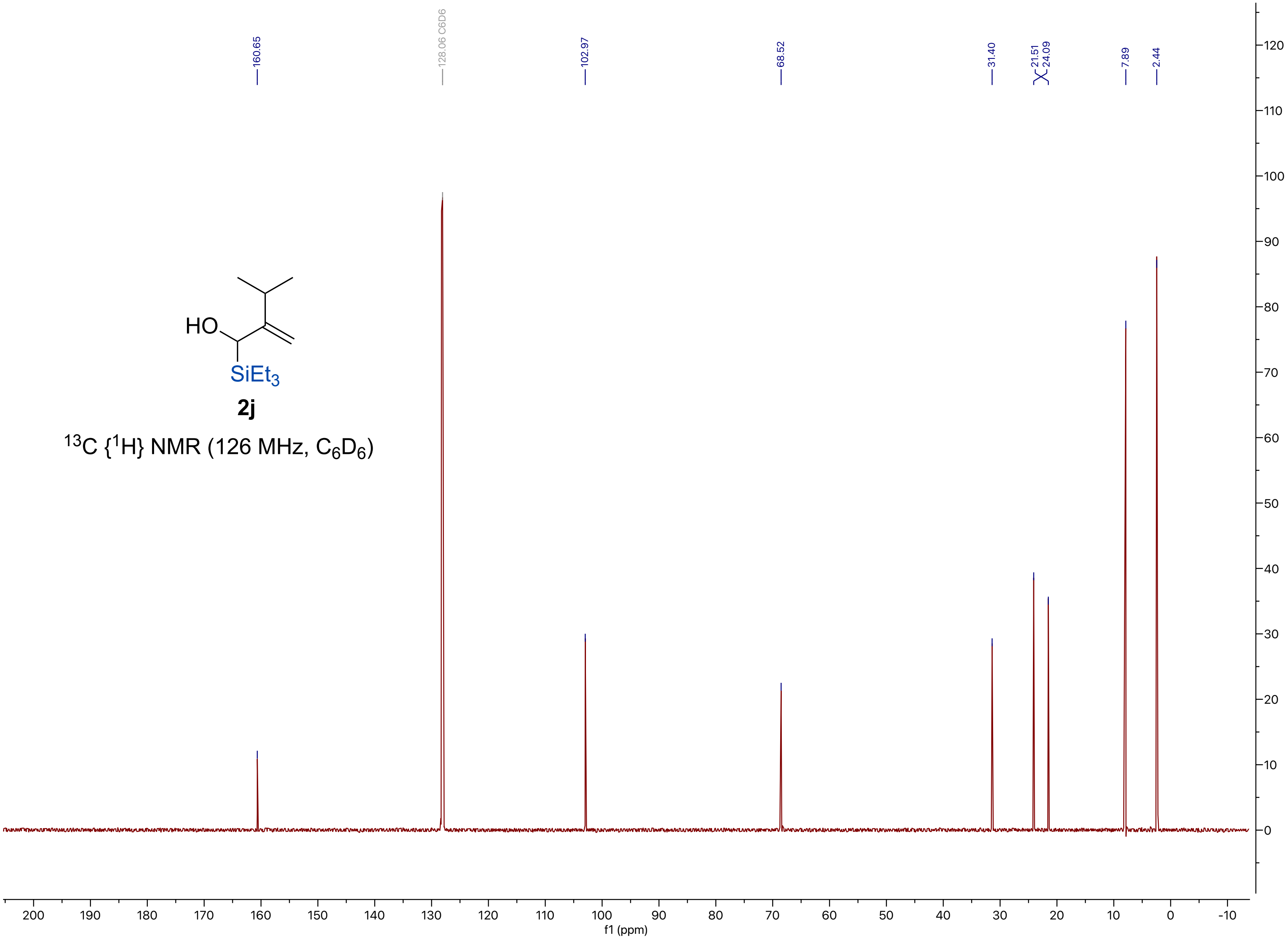

2j

$^{13}C\{^{1}H\}$ NMR (126 MHz, C_6D_6)

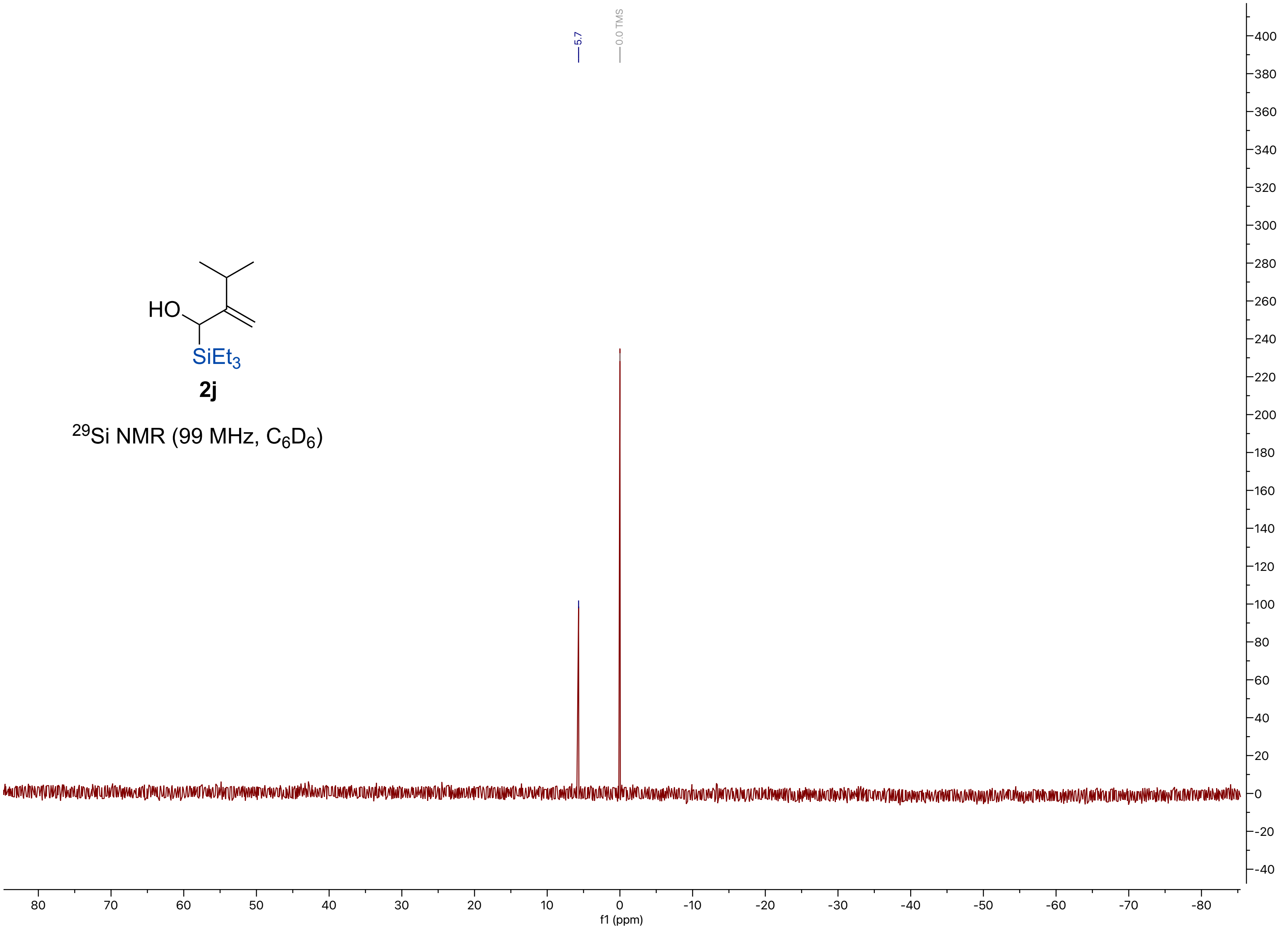

2j

^{29}Si NMR (99 MHz, C_6D_6)

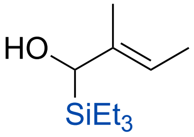

2k

^{1}H NMR (500 MHz, C_6D_6)

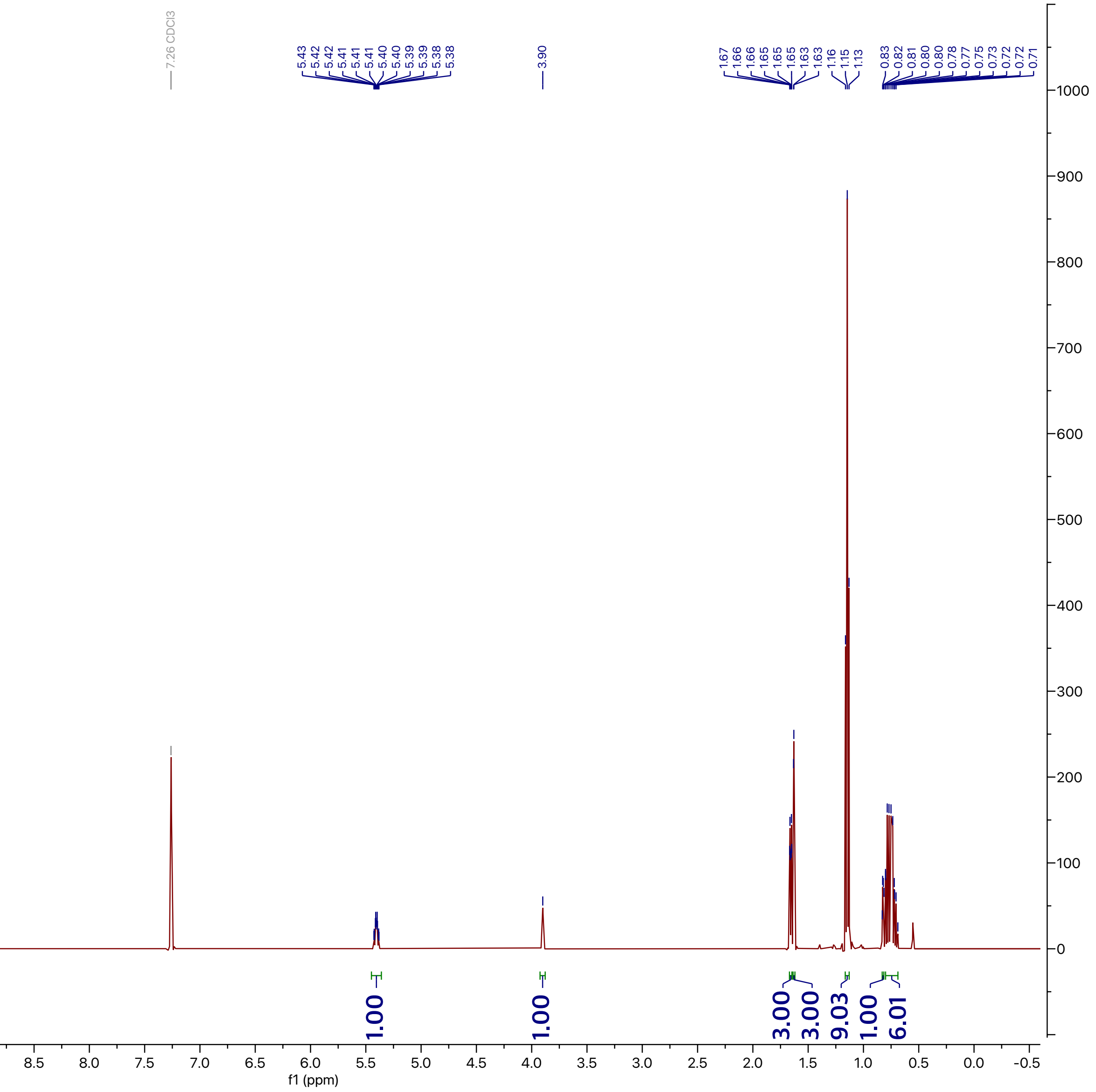

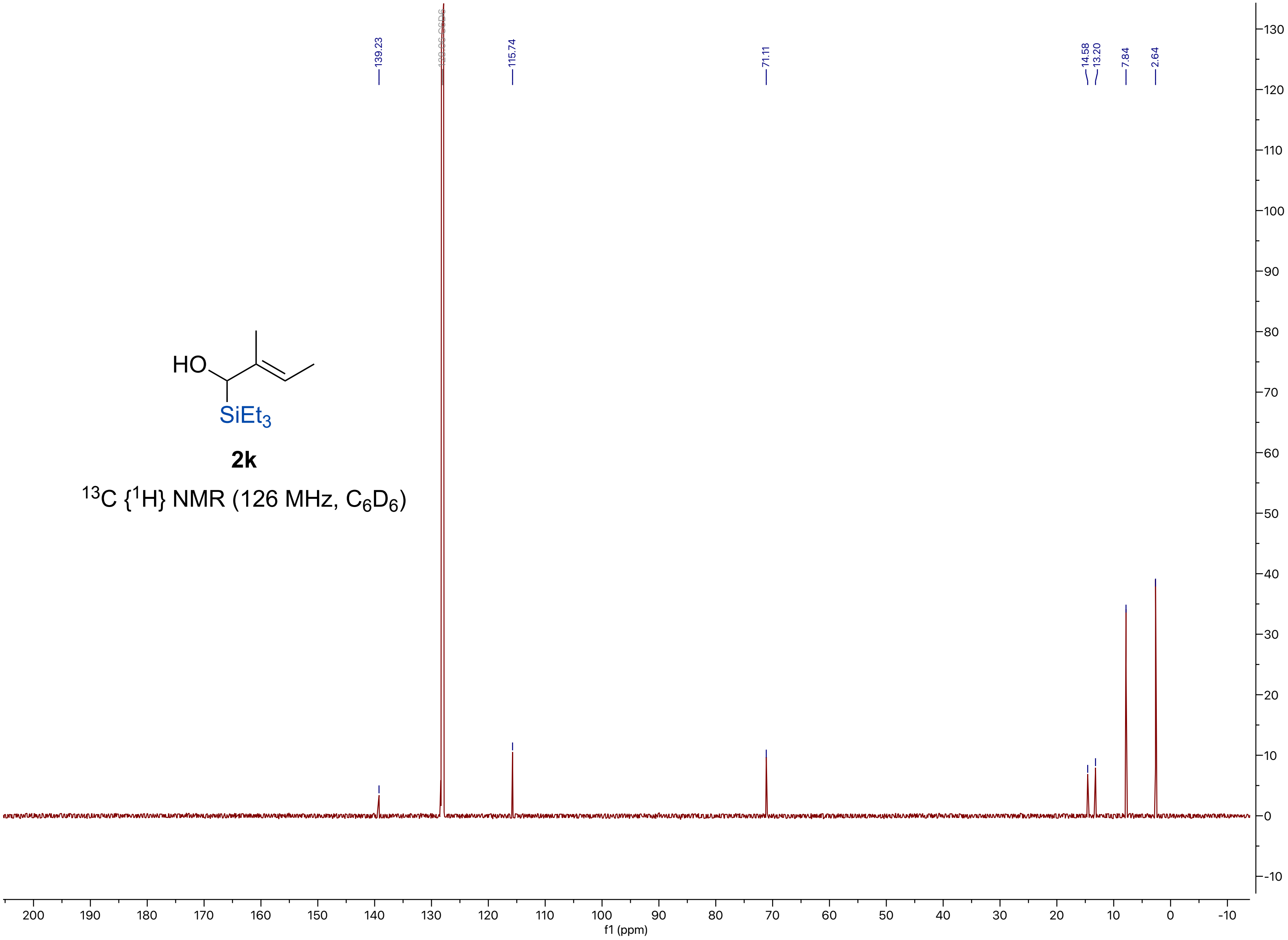

139.23
115.74
71.11
14.58
13.20
7.84
2.64
HO
SiEt3
2k
13C {1H} NMR (126 MHz, C6D6)
f1 (ppm)

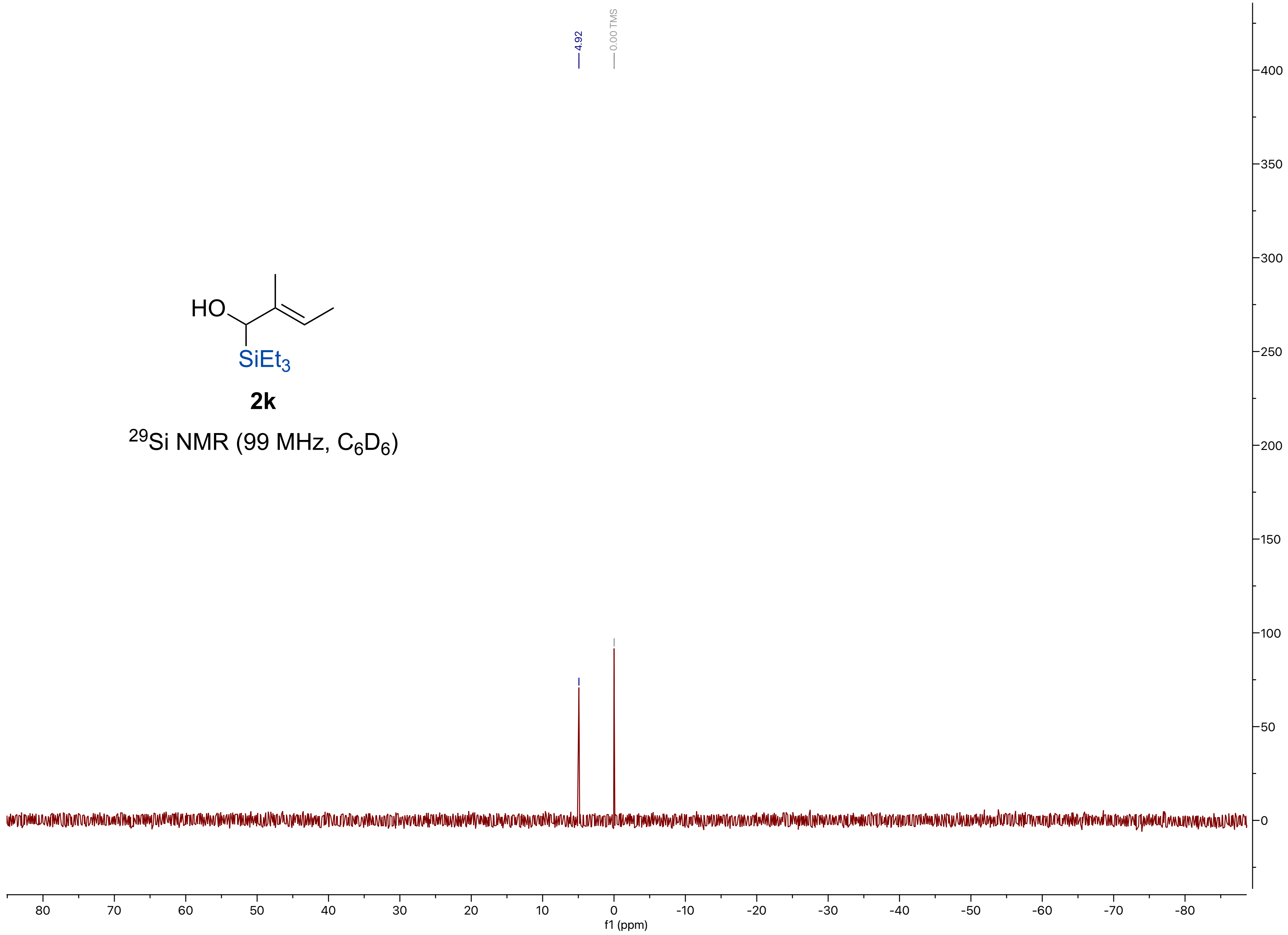

2k

^{29}Si NMR (99 MHz, C_6D_6)

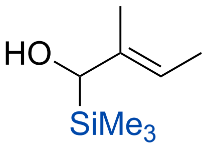

2l

^{1}H NMR (500 MHz, C_6D_6)

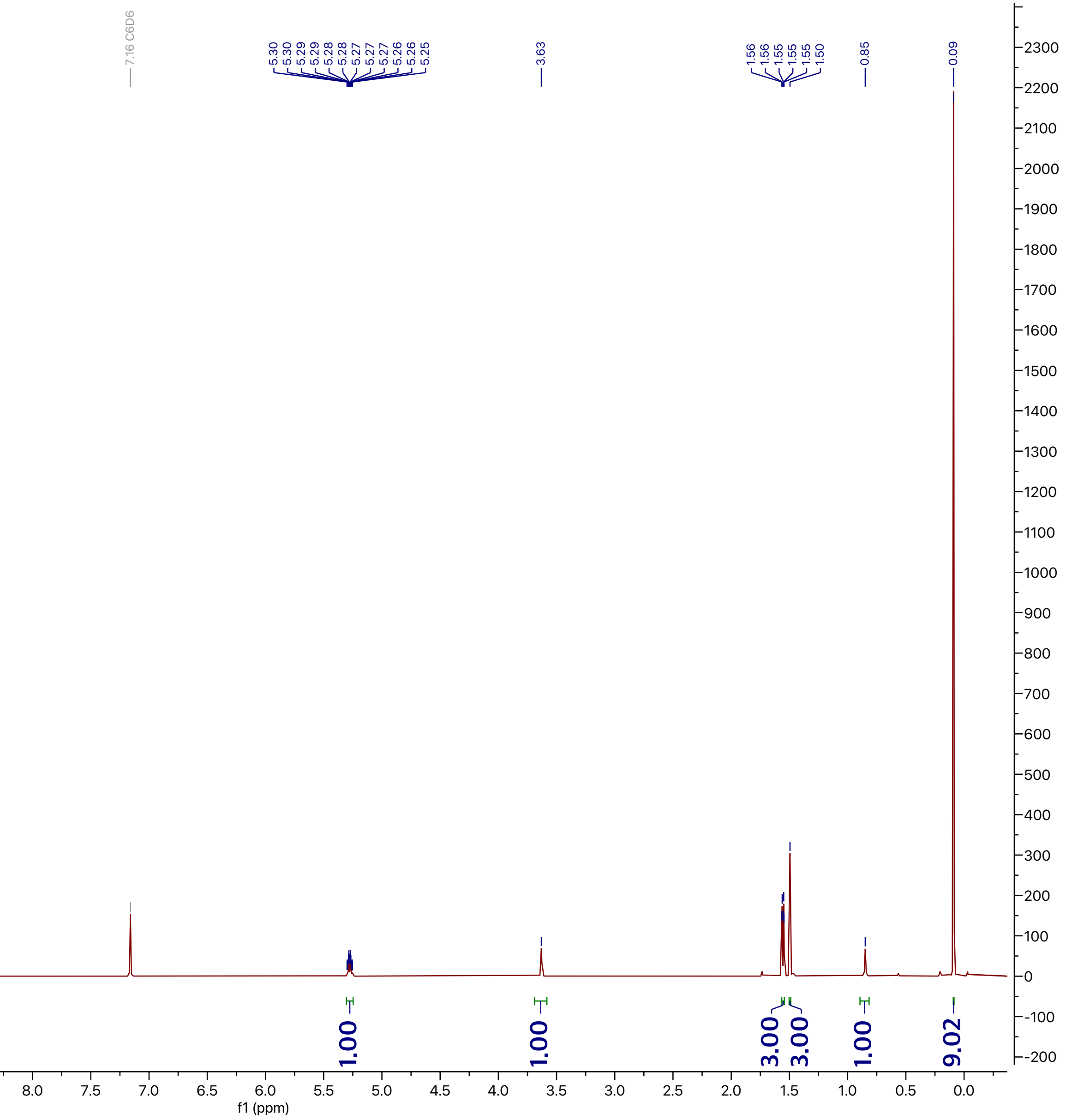

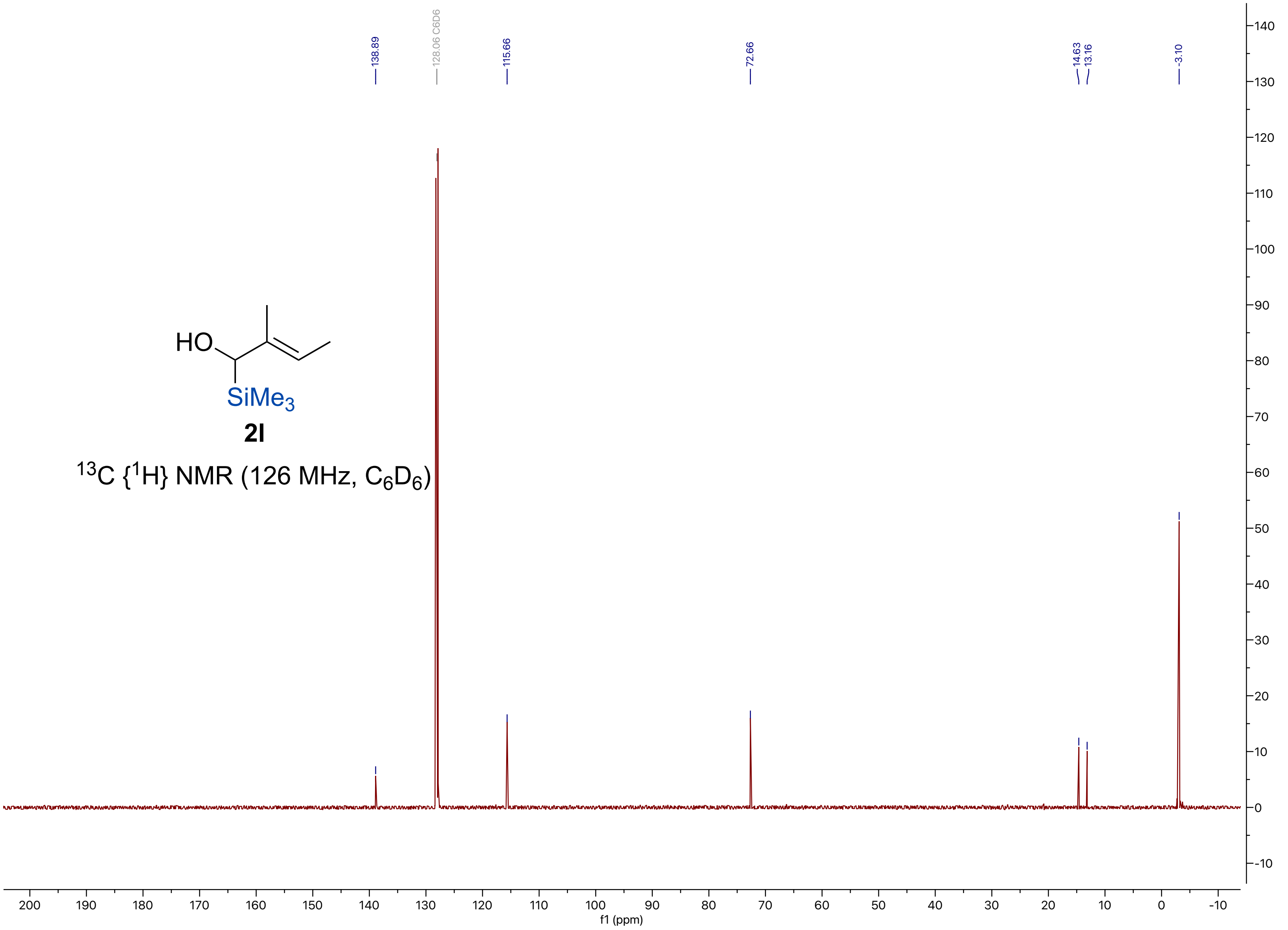
HO
SiMe3
2l
13C {1H} NMR (126 MHz, C6D6)
138.89
128.06 C6D6
115.66
72.66
14.63
13.16
-3.10
f1 (ppm)

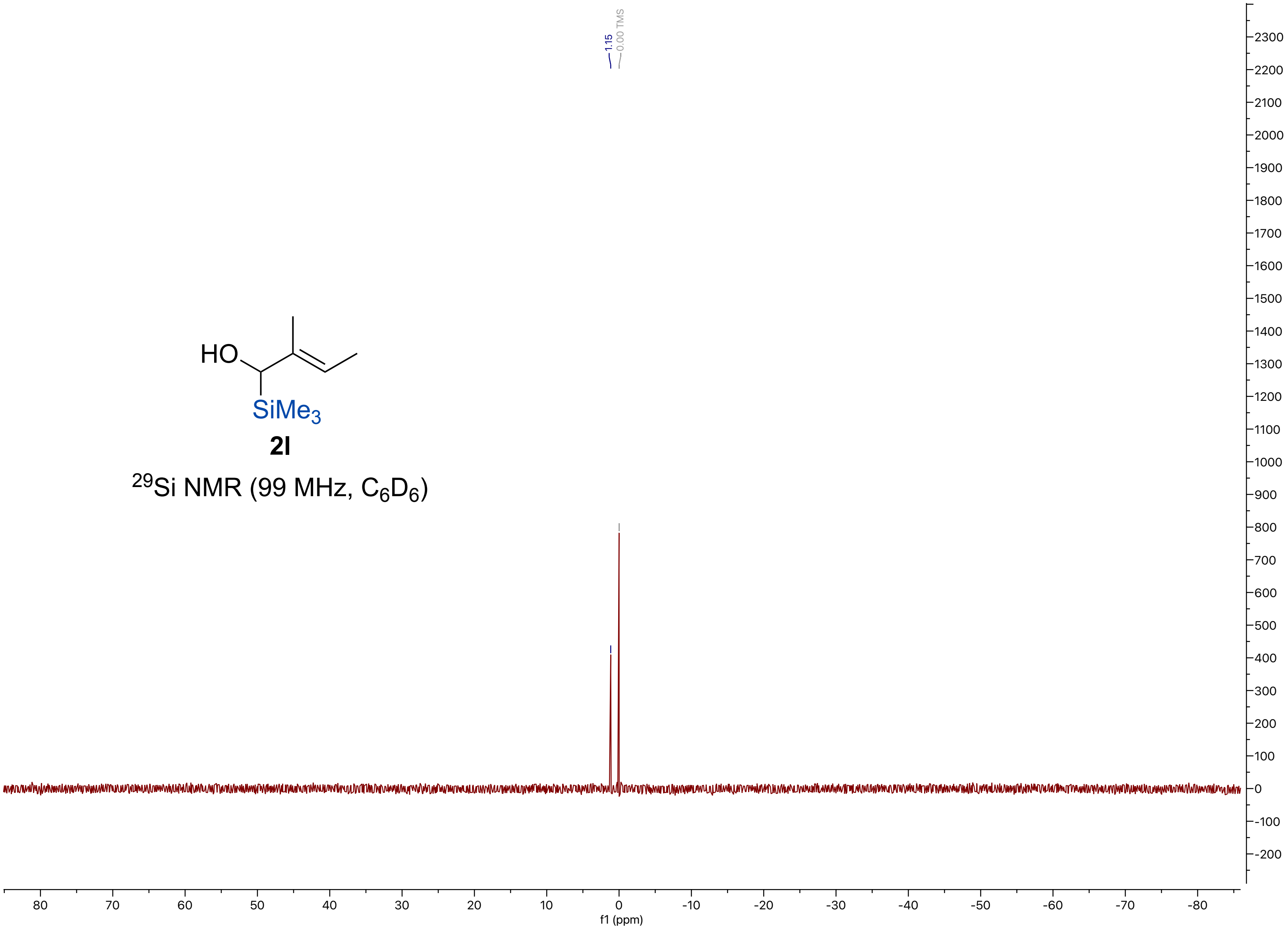

2l

^{29}Si NMR (99 MHz, C_6D_6)

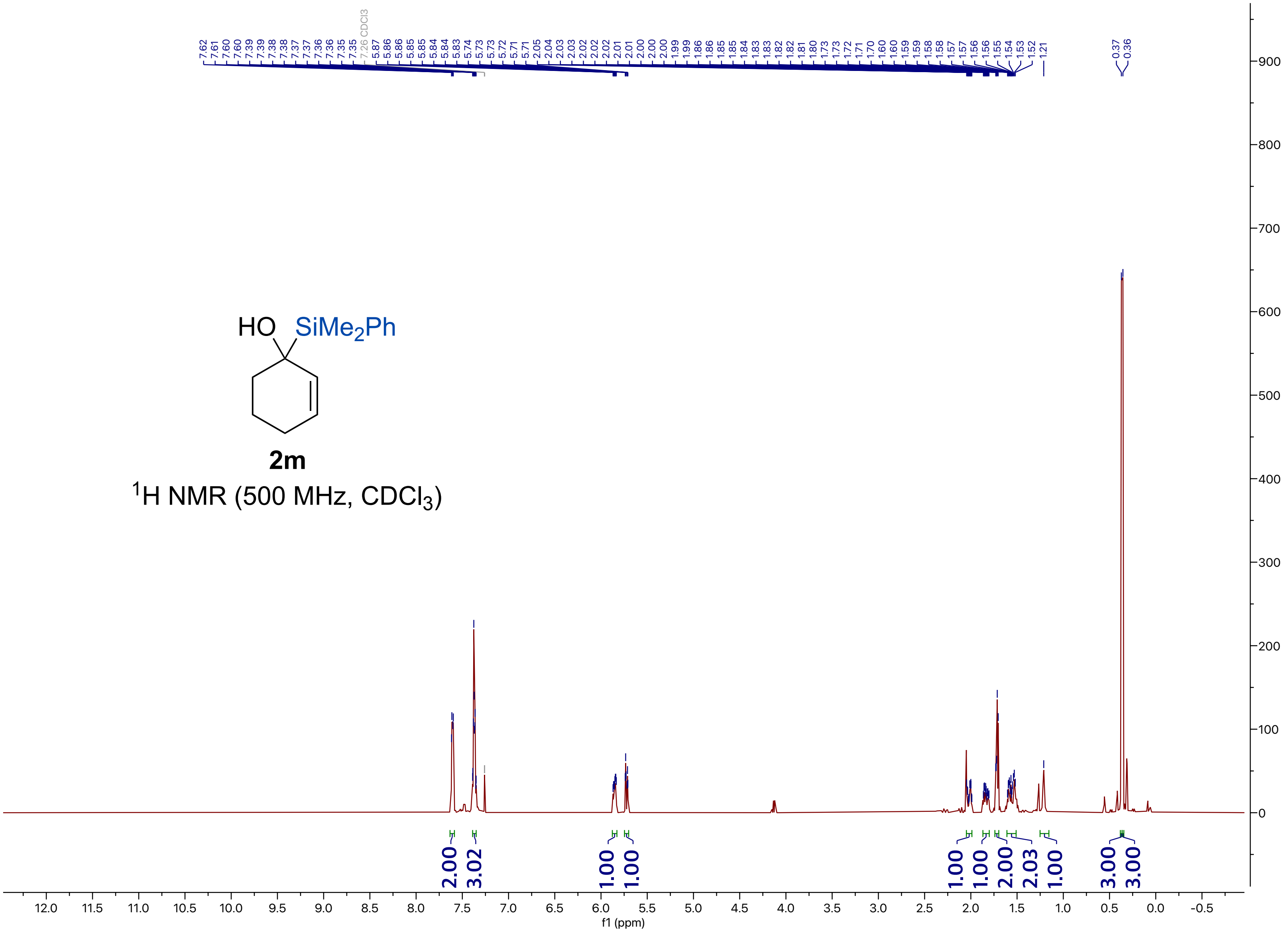

2m

^{1}H NMR (500 MHz, $CDCl_3$)

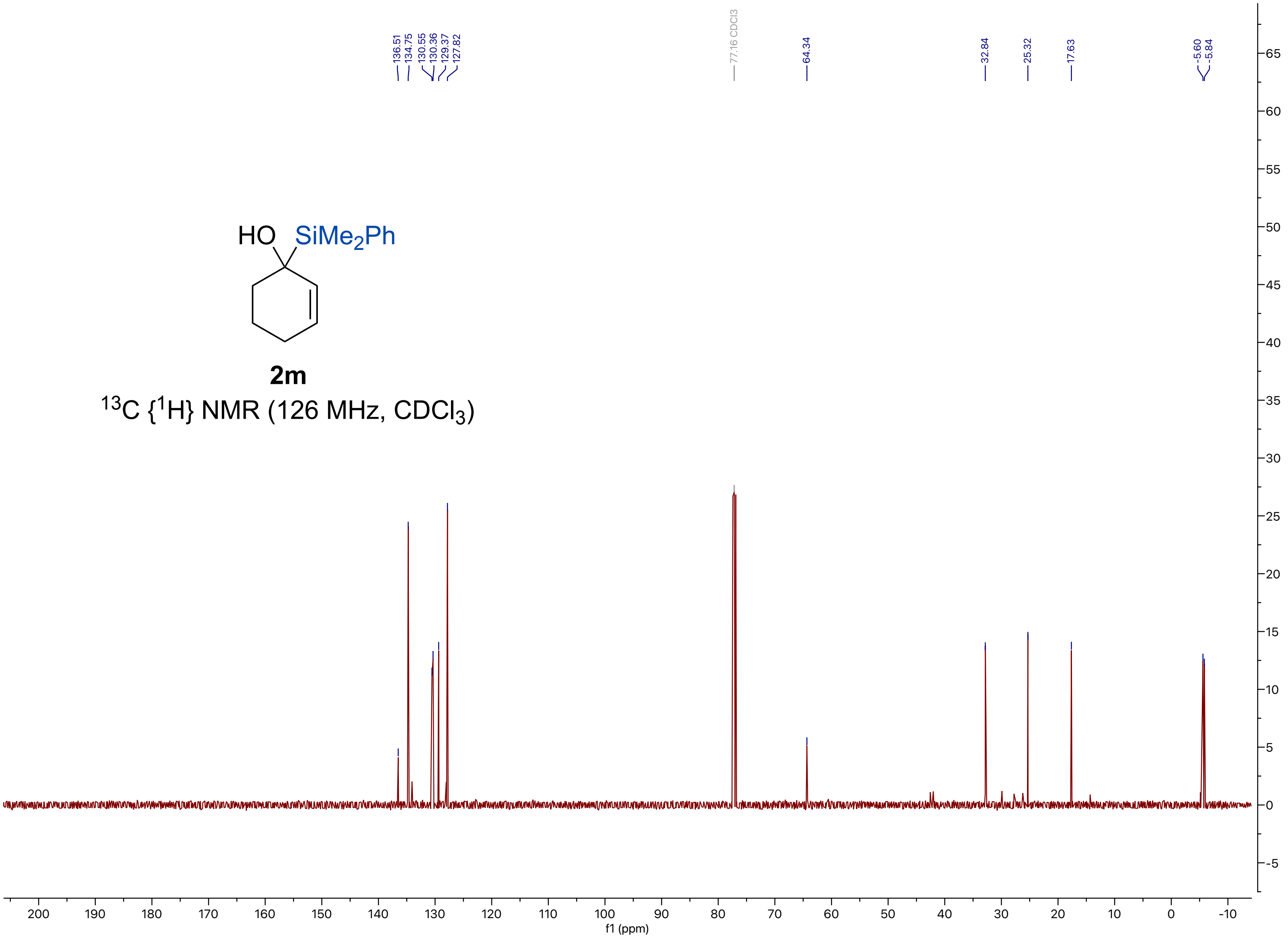

2m

$^{13}C\ \{^{1}H\}$ NMR (126 MHz, $CDCl_3$)

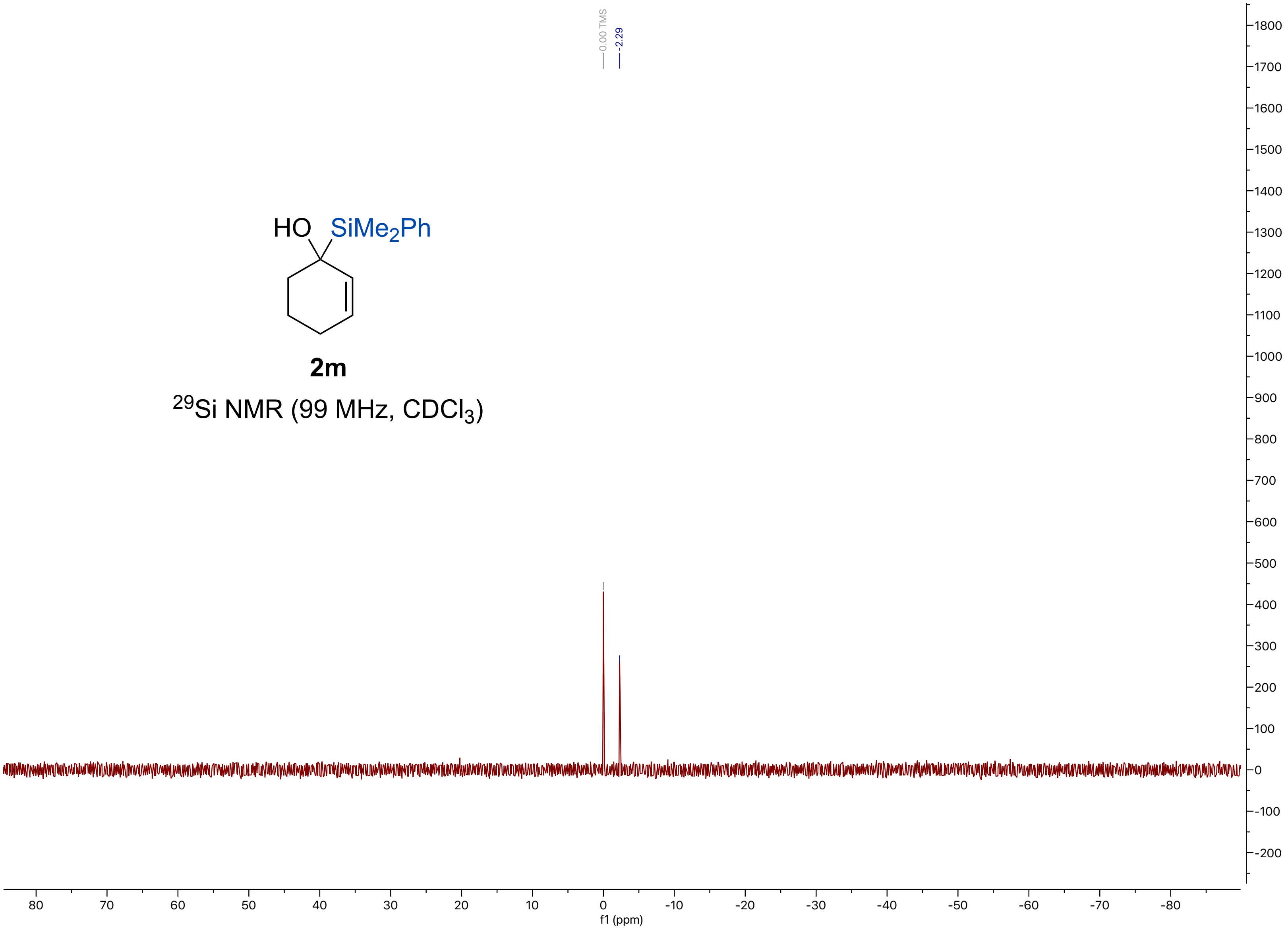

2m

^{29}Si NMR (99 MHz, $CDCl_3$)

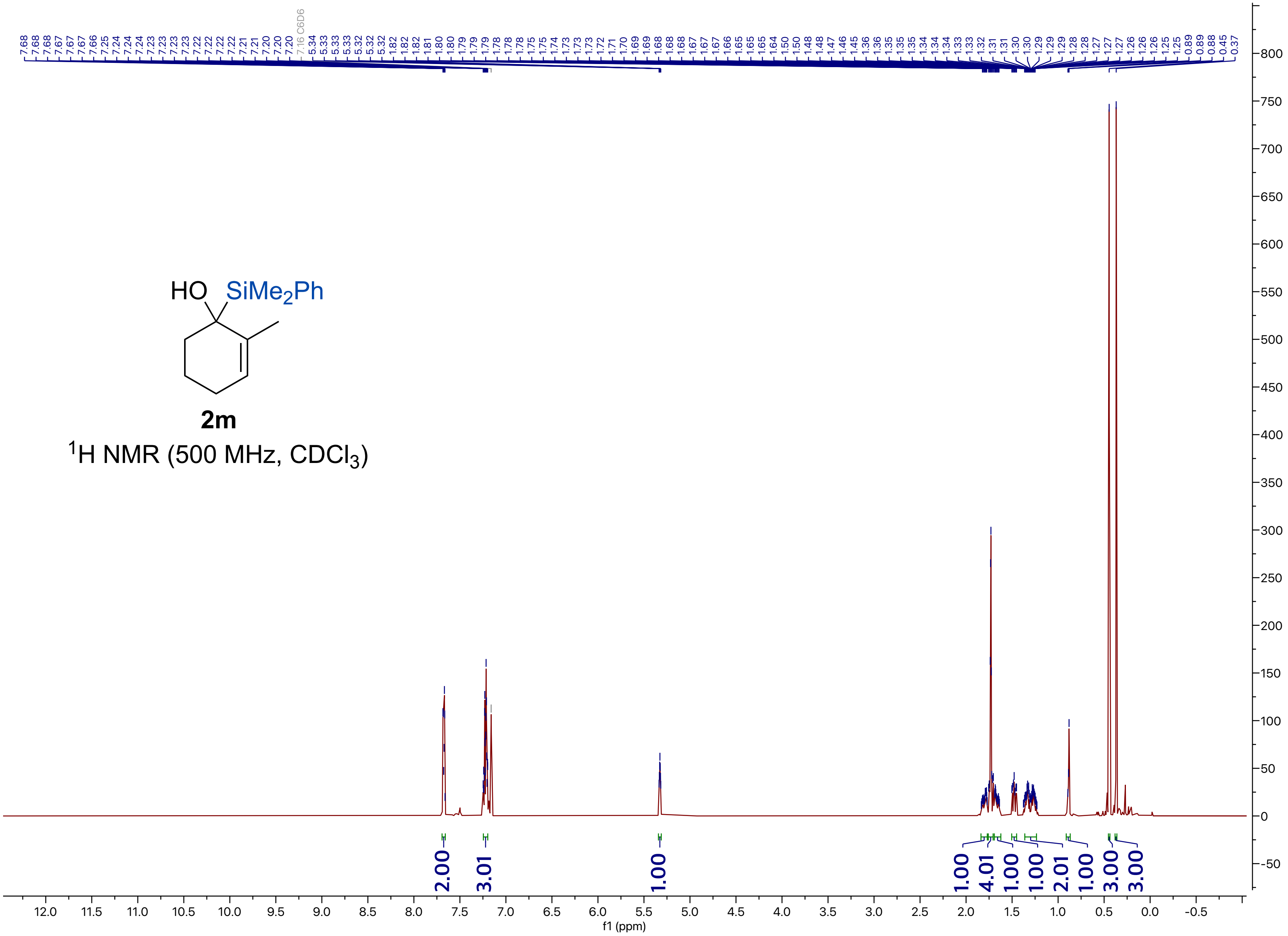

2m

^{1}H NMR (500 MHz, $CDCl_3$)

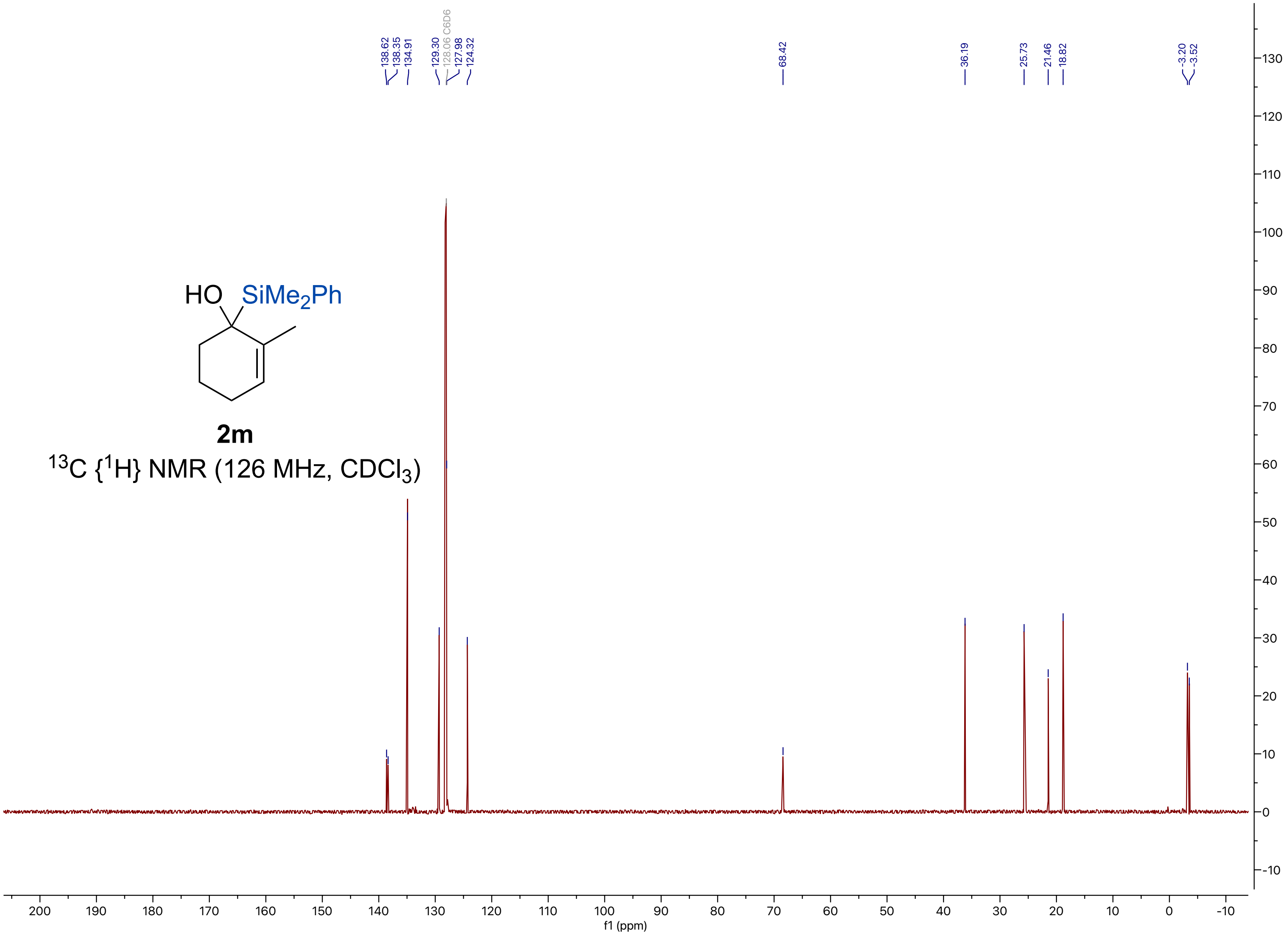

2m

^{13}C {^{1}H} NMR (126 MHz, $CDCl_3$)

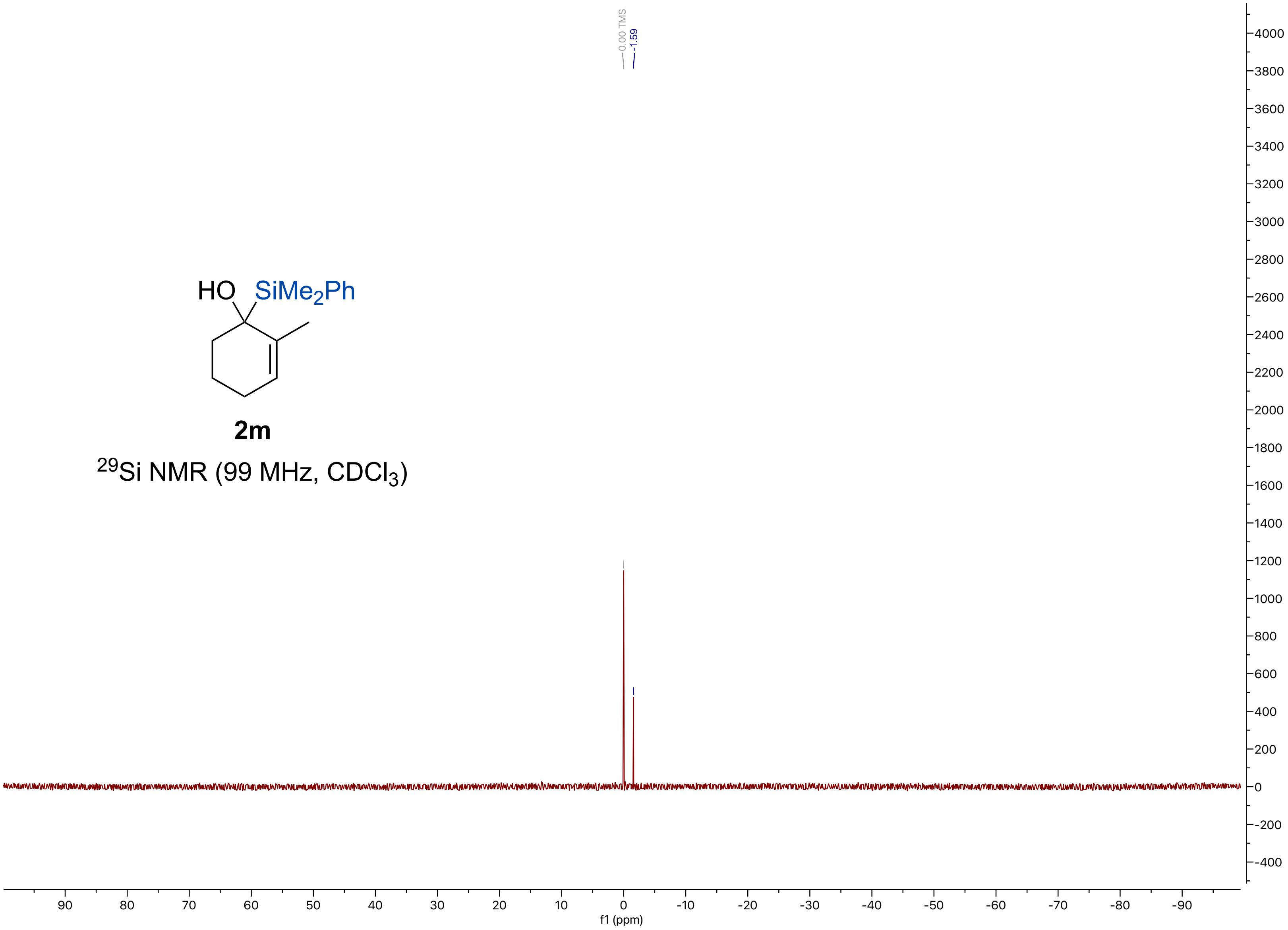

2m

^{29}Si NMR (99 MHz, $CDCl_3$)

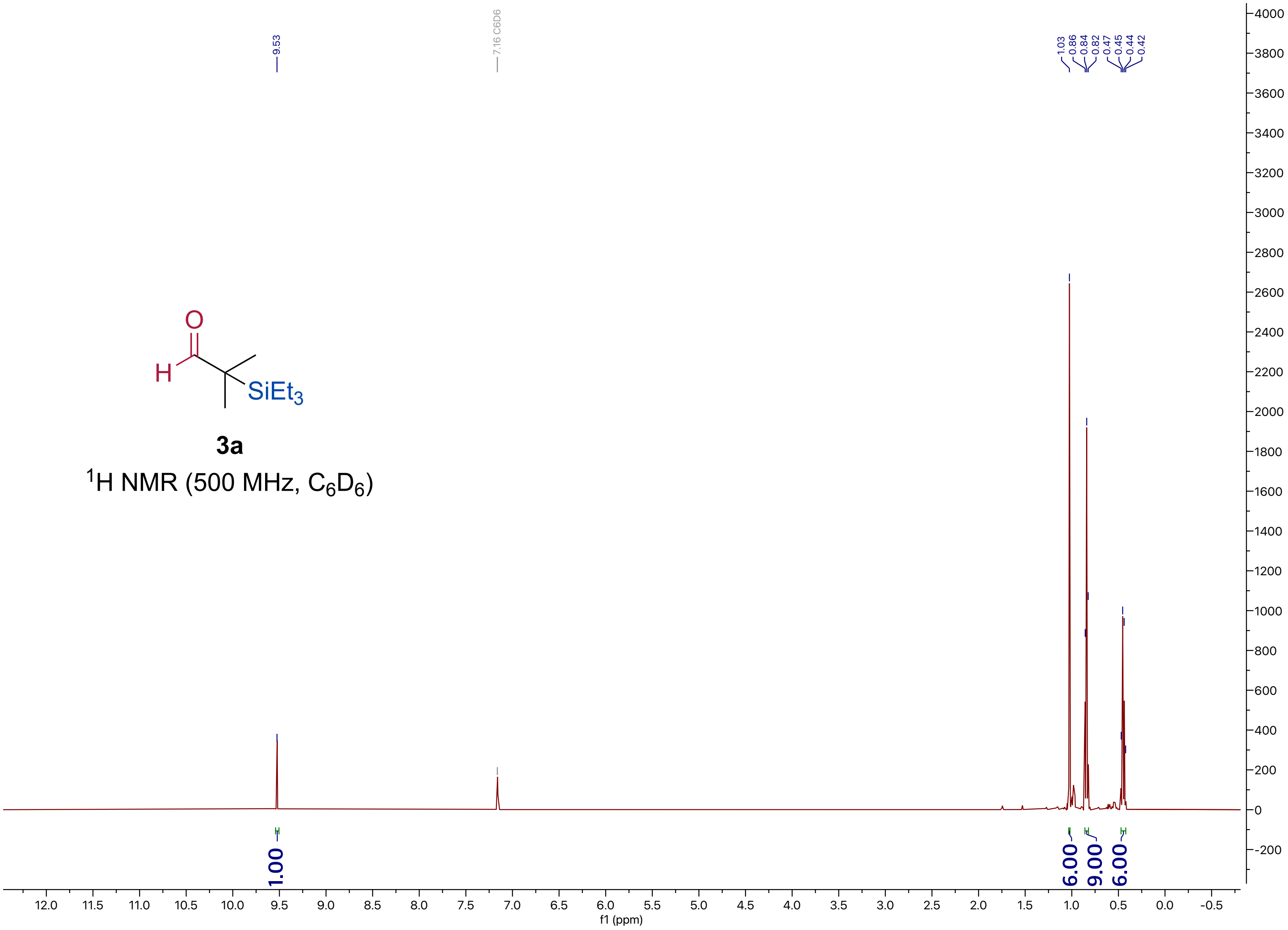

3a

^{1}H NMR (500 MHz, C_6D_6)

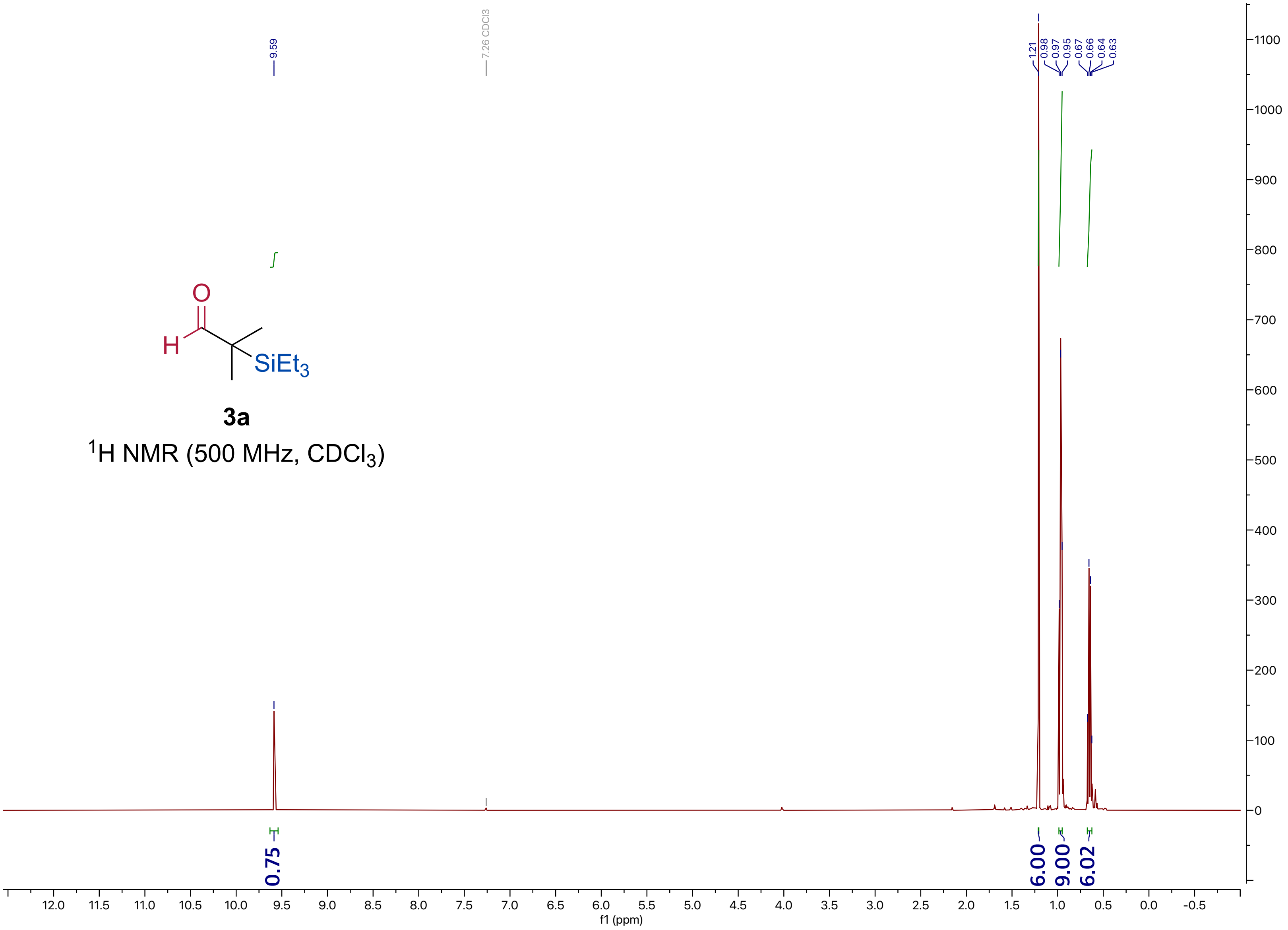

3a

^{1}H NMR (500 MHz, $CDCl_3$)

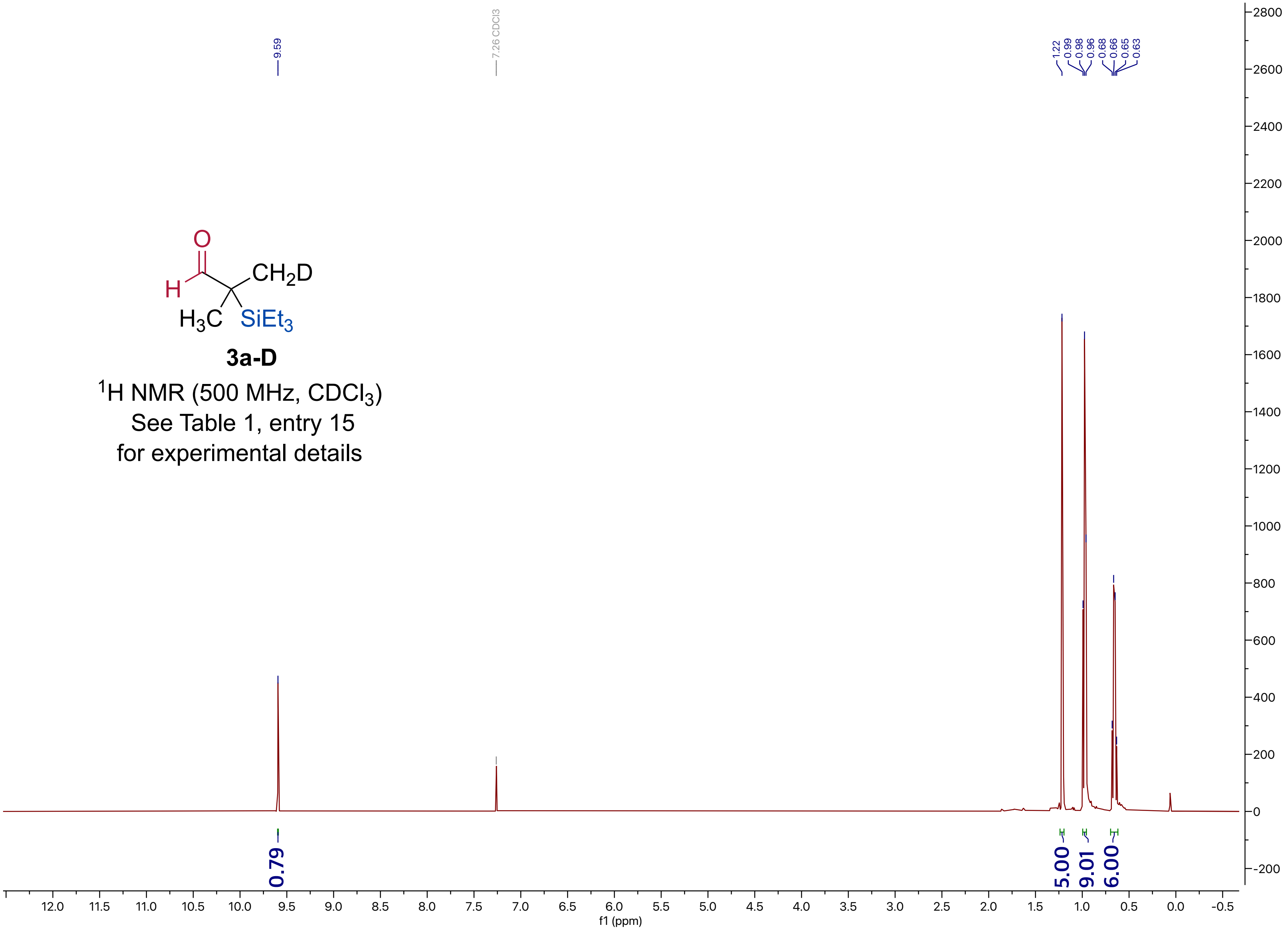

3a-D

^{1}H NMR (500 MHz, $CDCl_3$)
See Table 1, entry 15
for experimental details

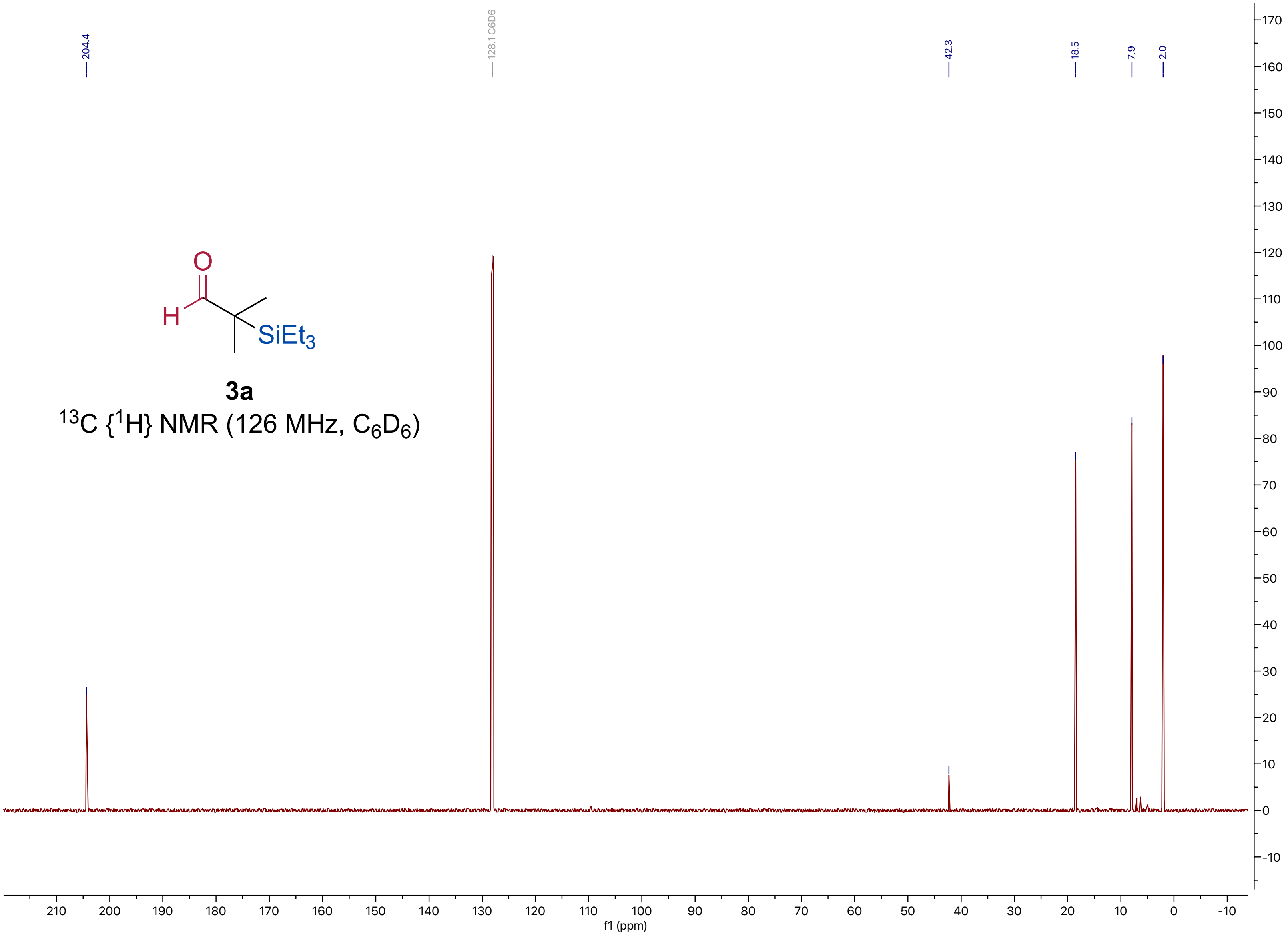

3a

$^{13}C\{^1H\}$ NMR (126 MHz, C_6D_6)

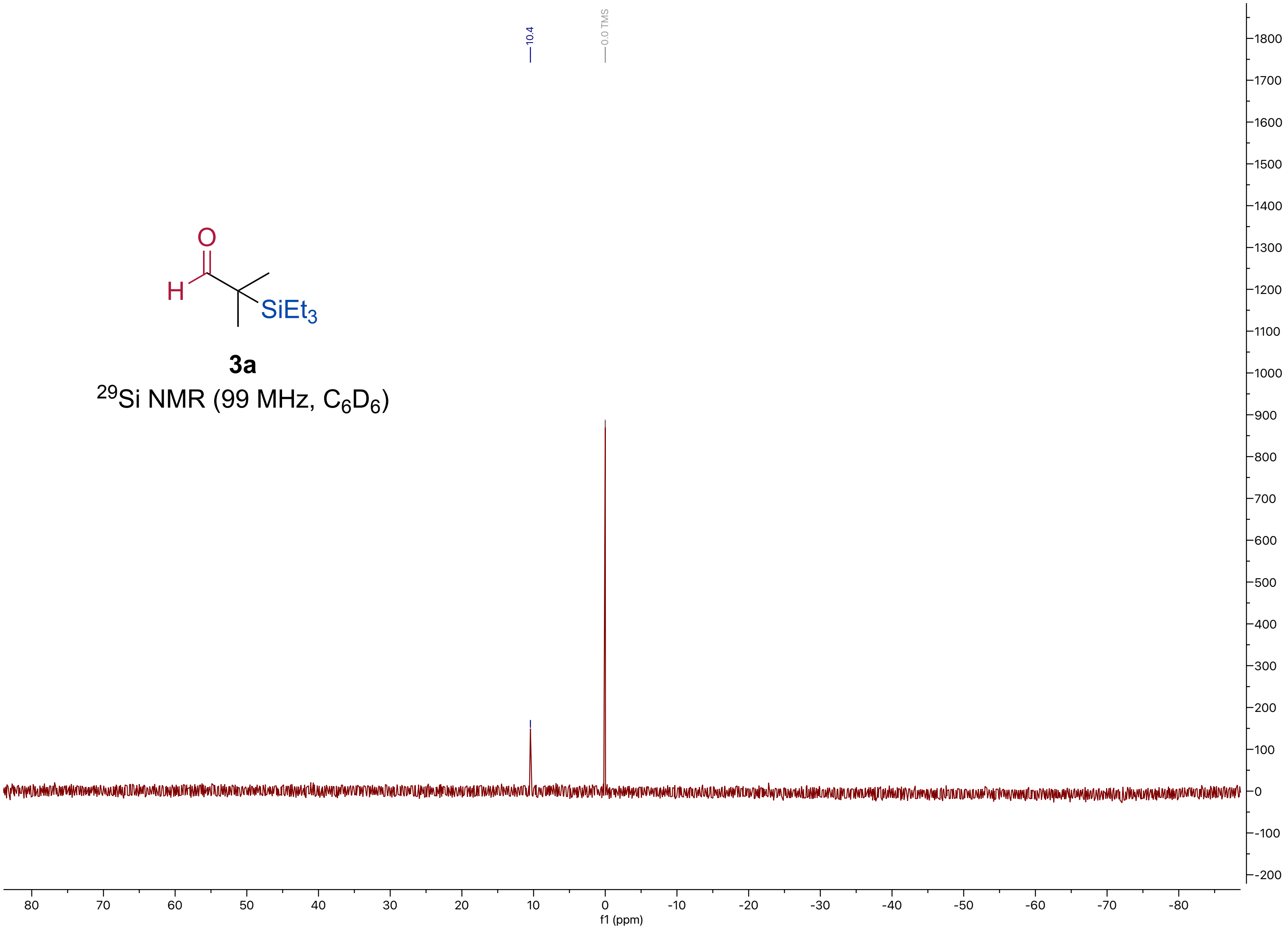

O
H
SiEt3
3a
29Si NMR (99 MHz, C6D6)
10.4
0.0 TMS
f1 (ppm)

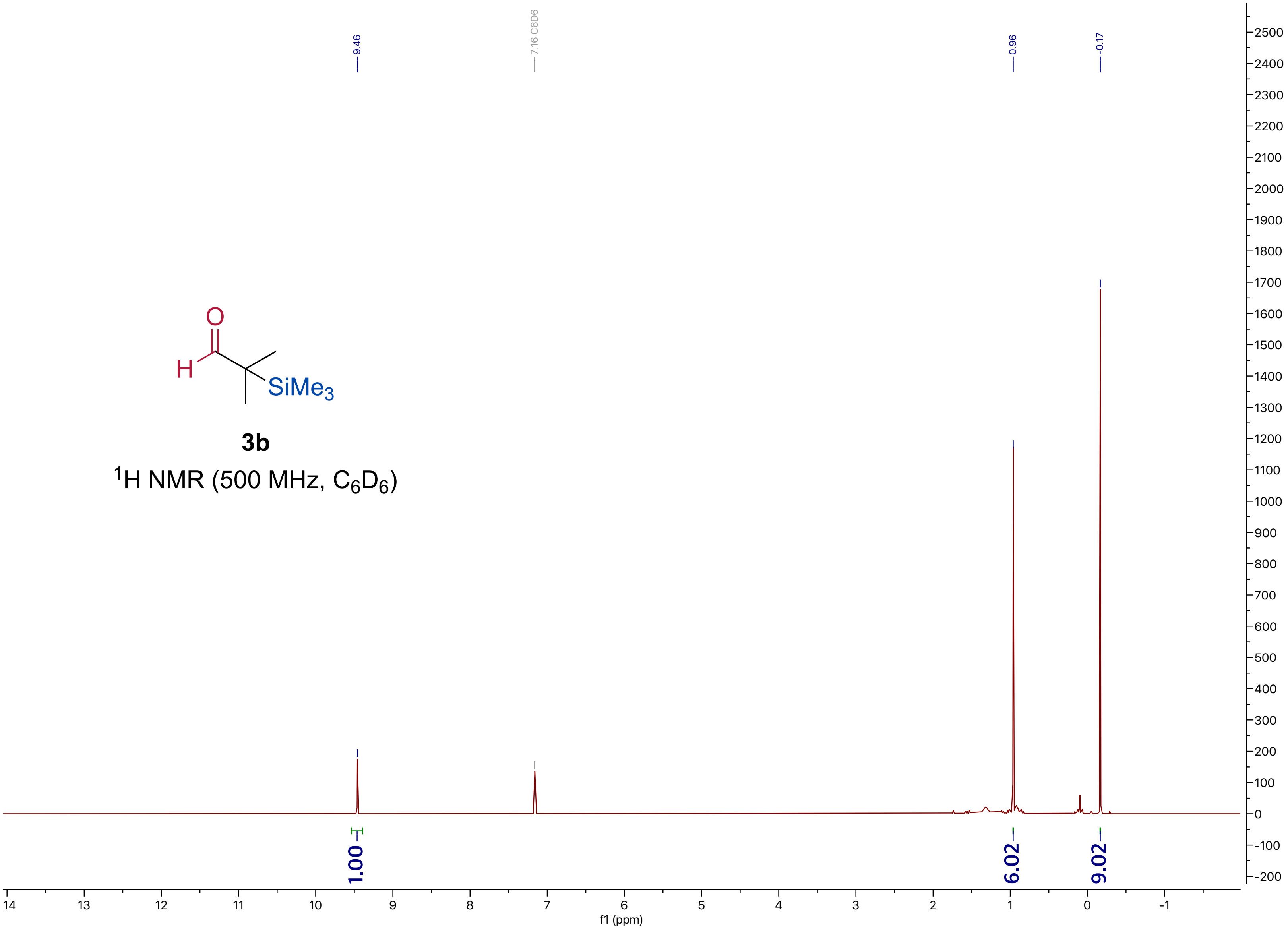

3b

^1H NMR (500 MHz, C_6D_6)

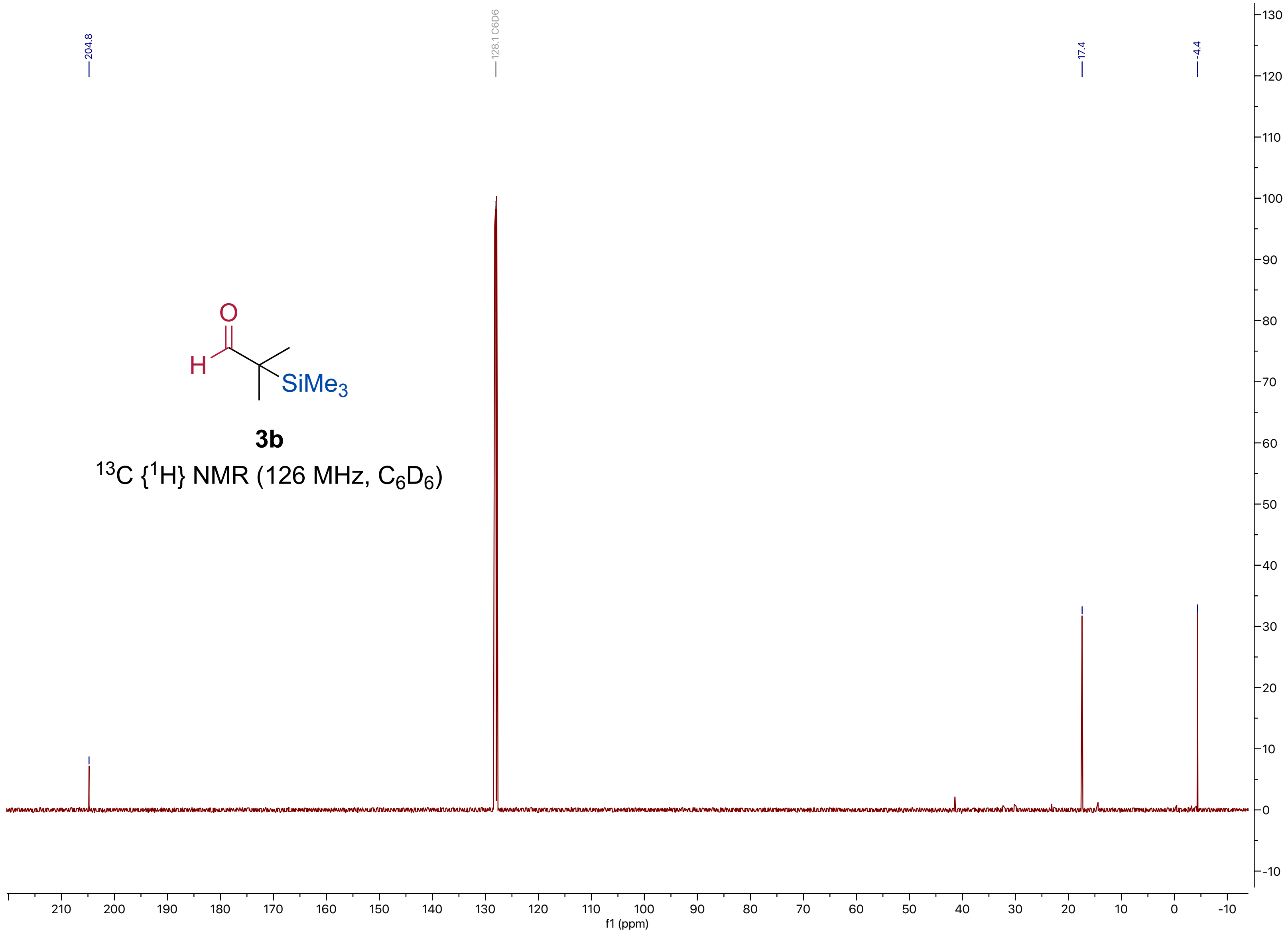

3b

$^{13}C\ \{^{1}H\}$ NMR (126 MHz, C_6D_6)

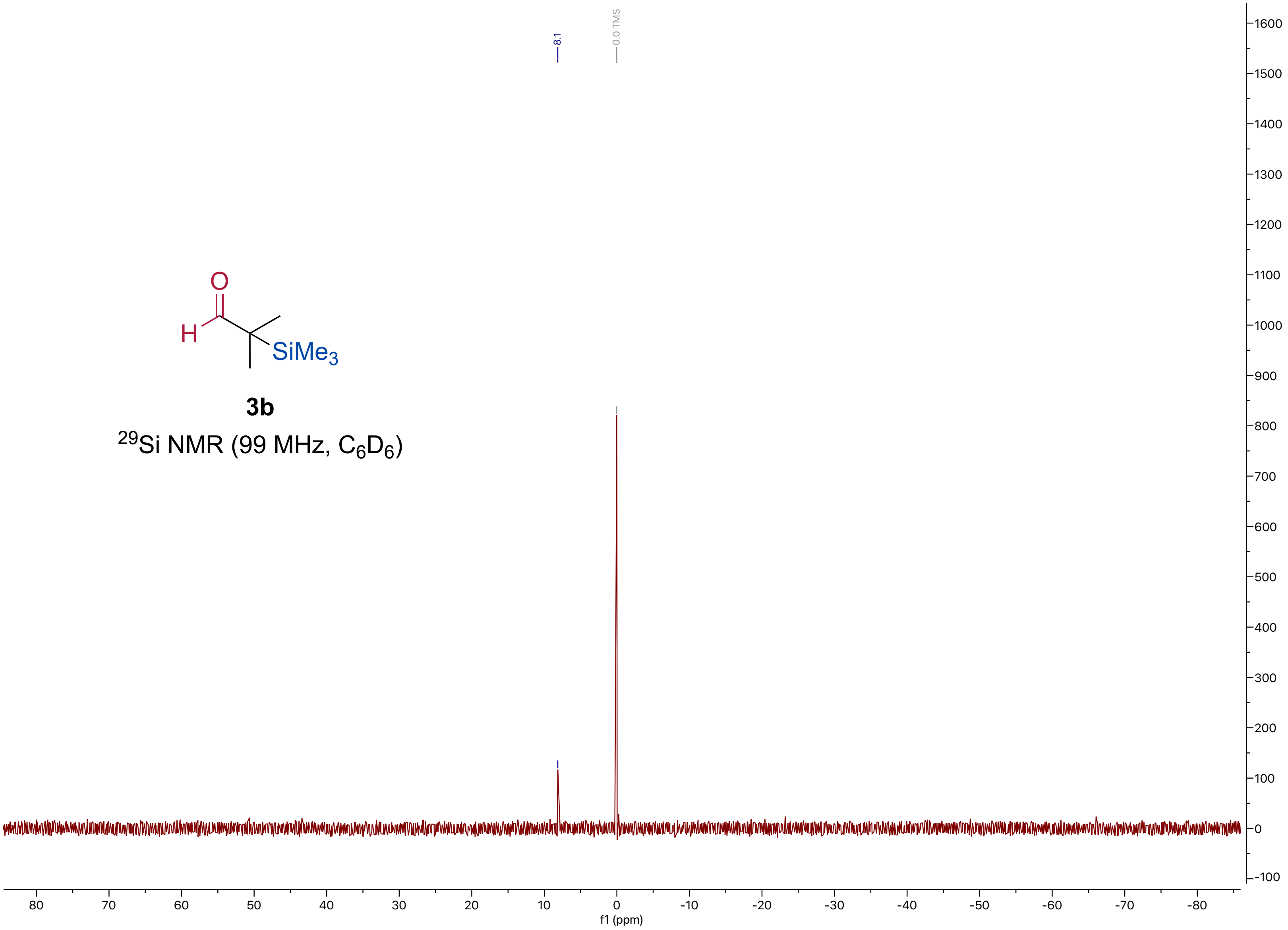

3b

^{29}Si NMR (99 MHz, C_6D_6)

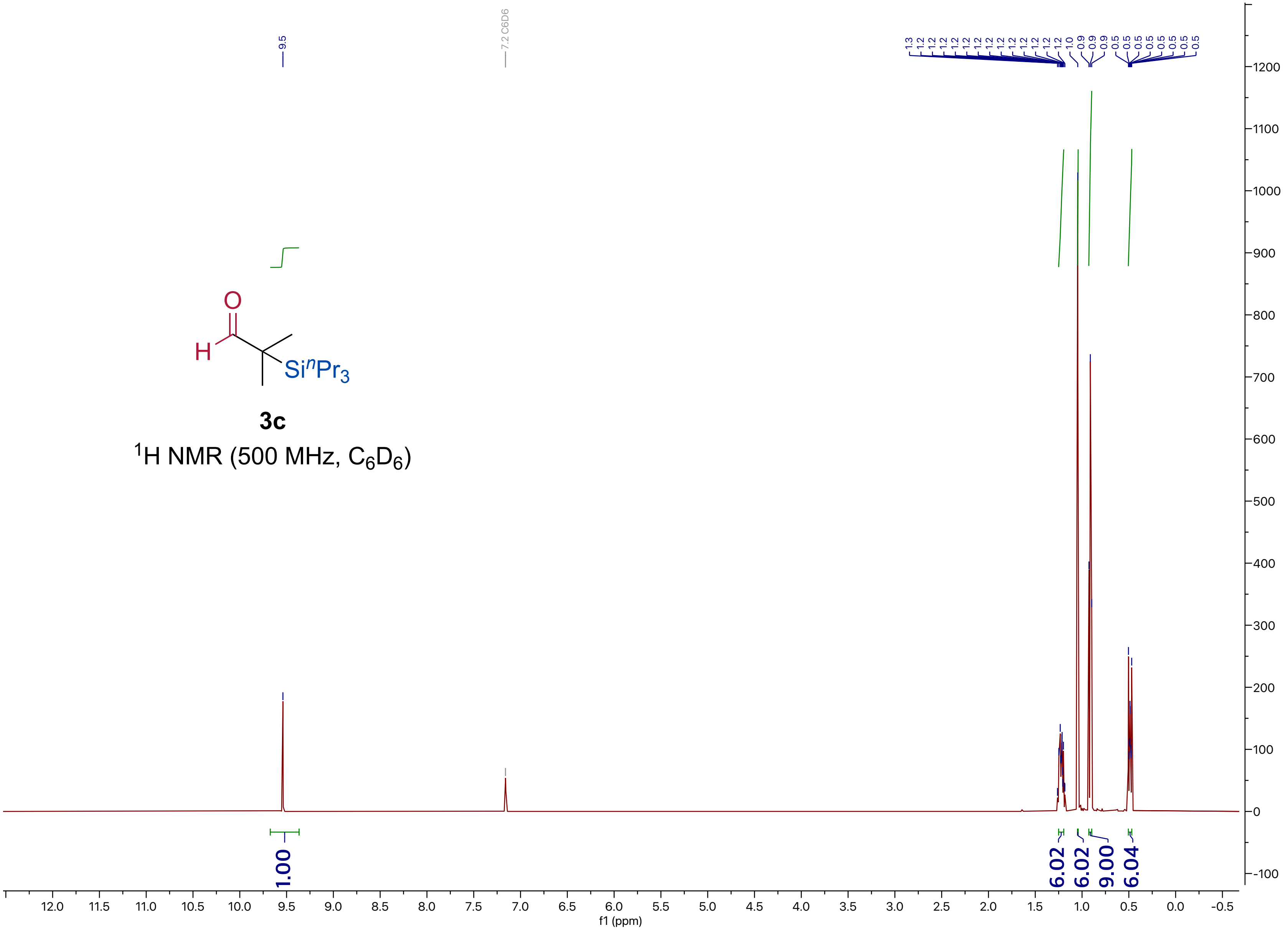

3c

^{1}H NMR (500 MHz, C_6D_6)

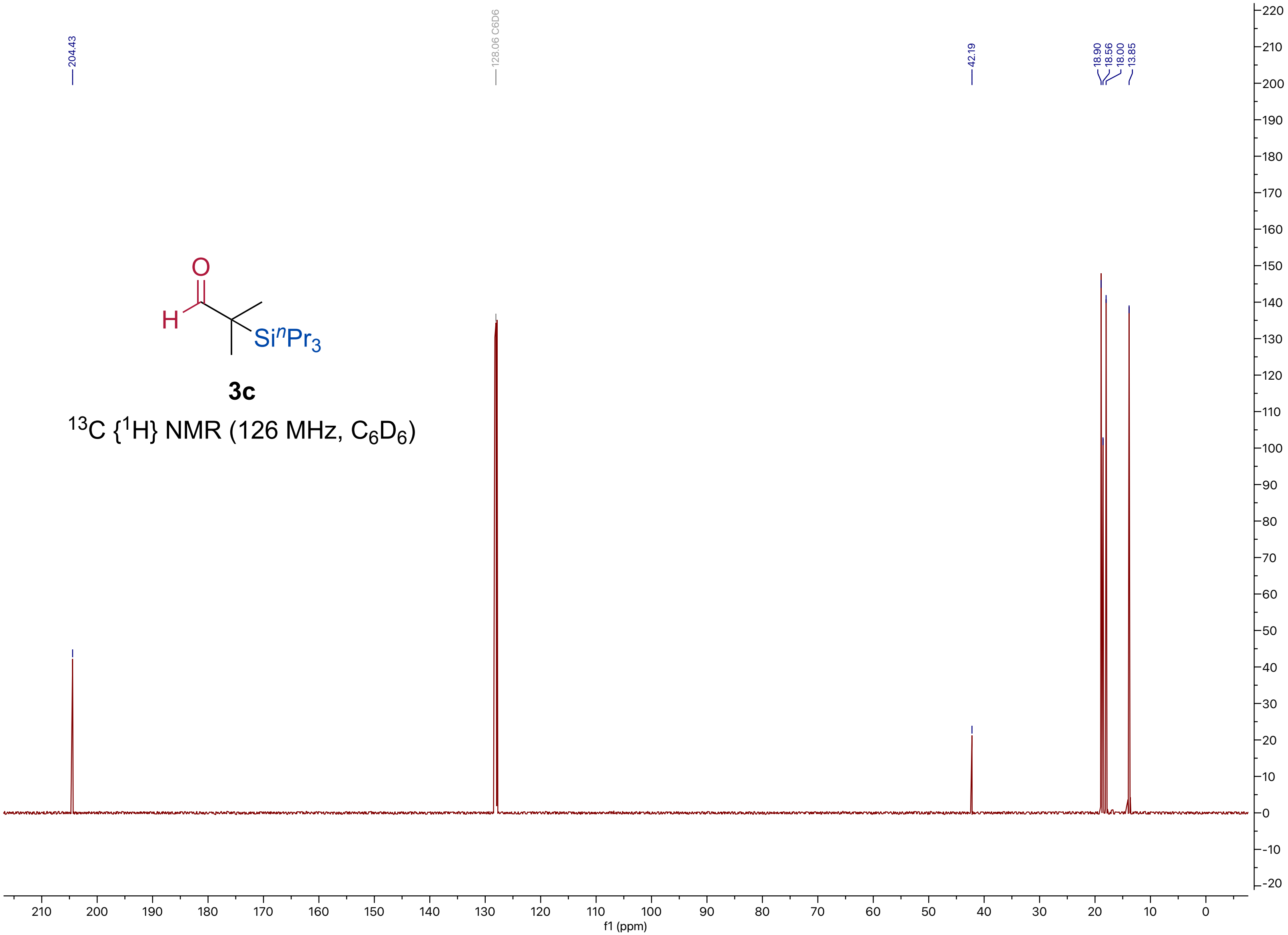

3c

$^{13}C\ \{^1H\}$ NMR (126 MHz, C_6D_6)

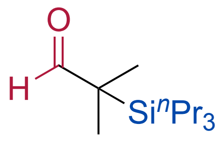

3c

^{29}Si NMR (99 MHz, C_6D_6)

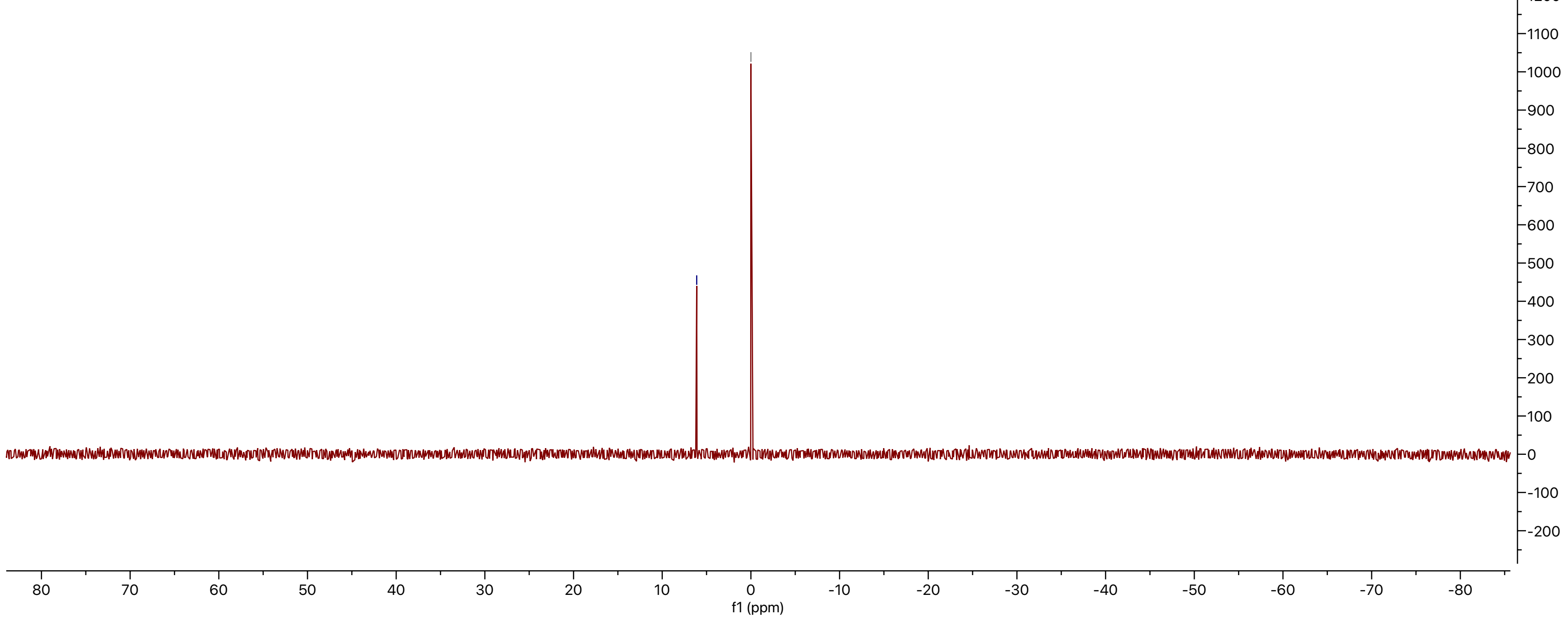

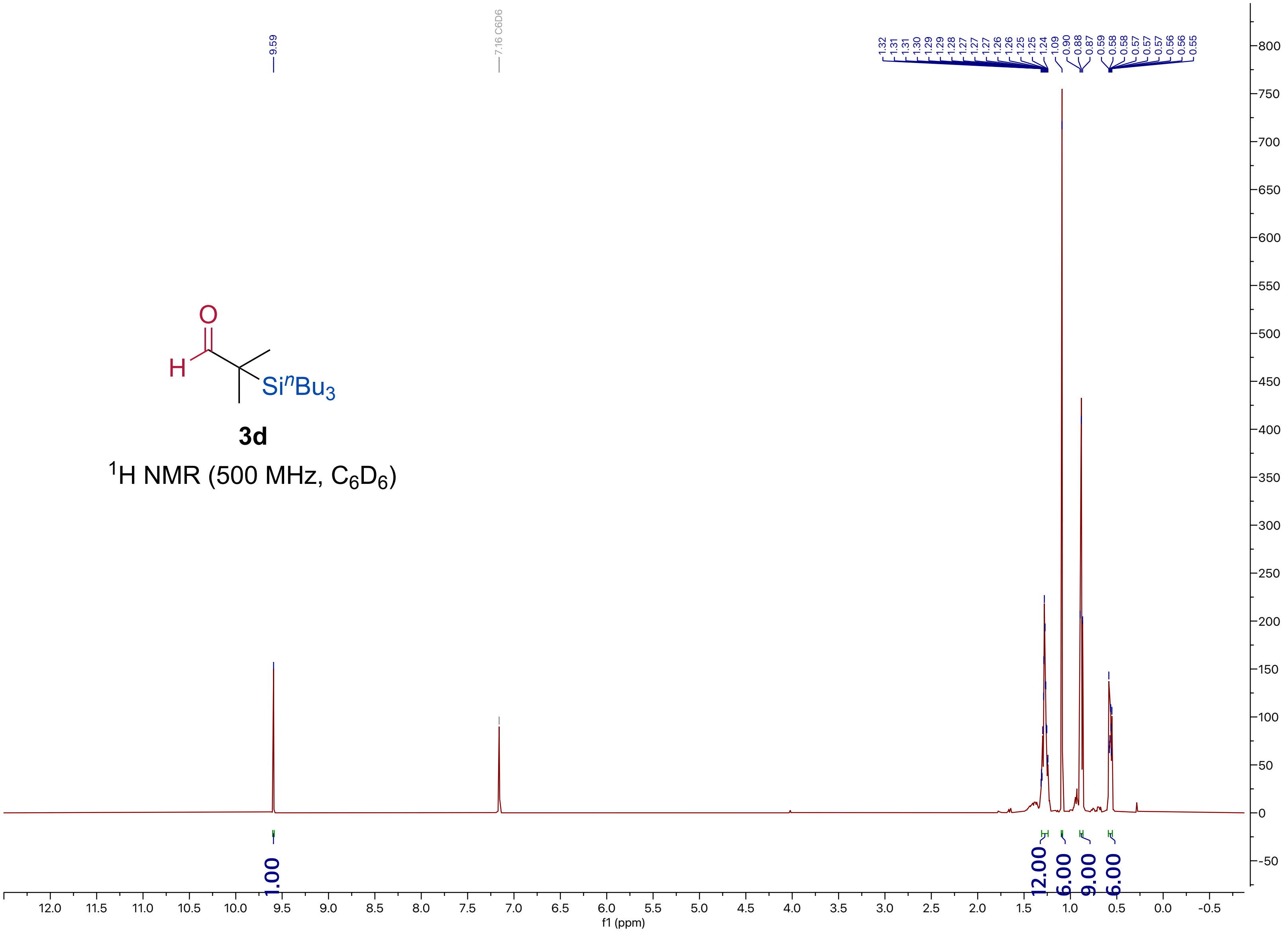

3d

^{1}H NMR (500 MHz, C_6D_6)

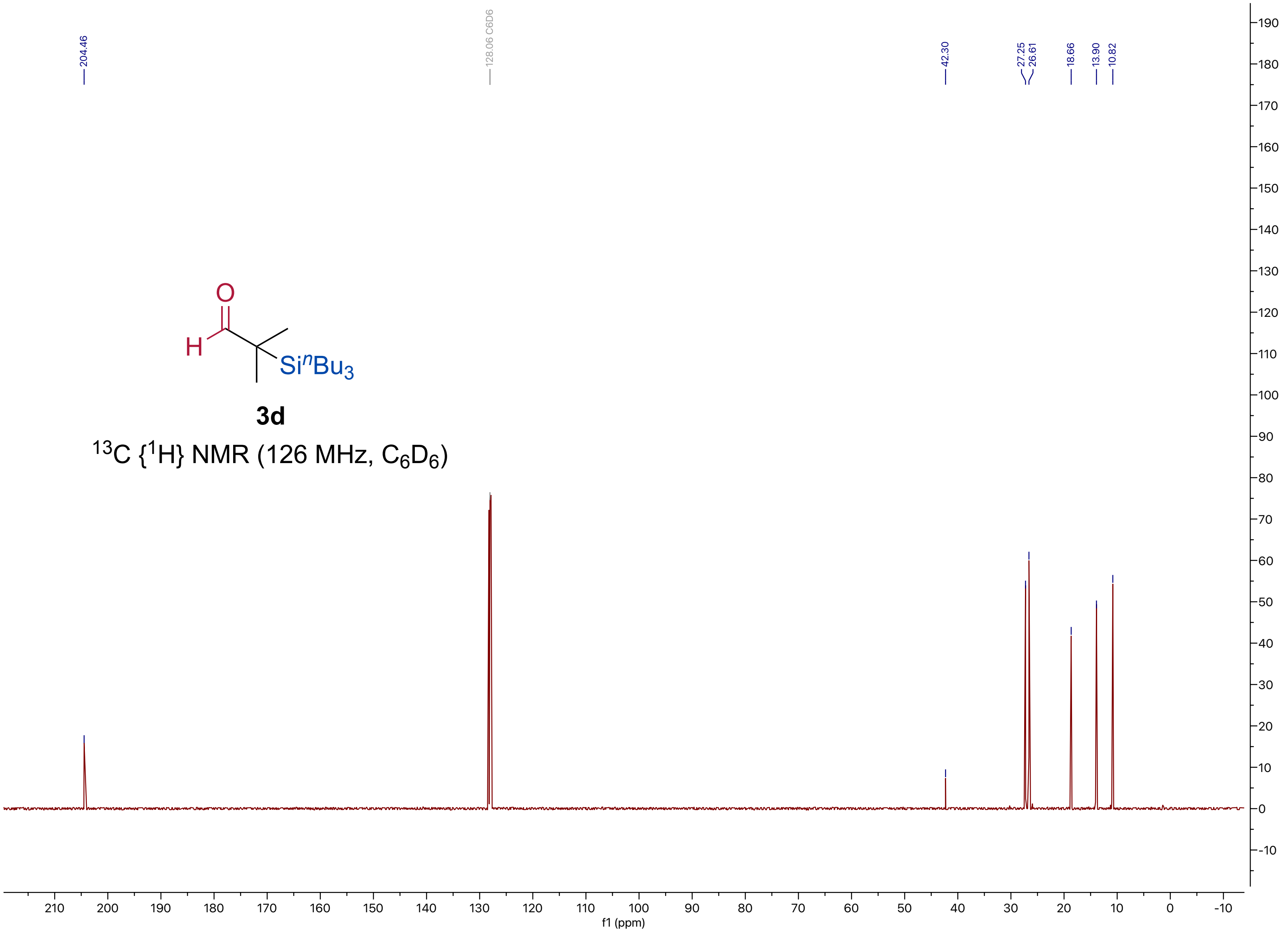

204.46
128.06 C6D6
42.30
27.25
26.61
18.66
13.90
10.82
O
H
Si^{n}Bu$_3$
3d
^{13}C {^{1}H} NMR (126 MHz, C_6D_6)
f1 (ppm)

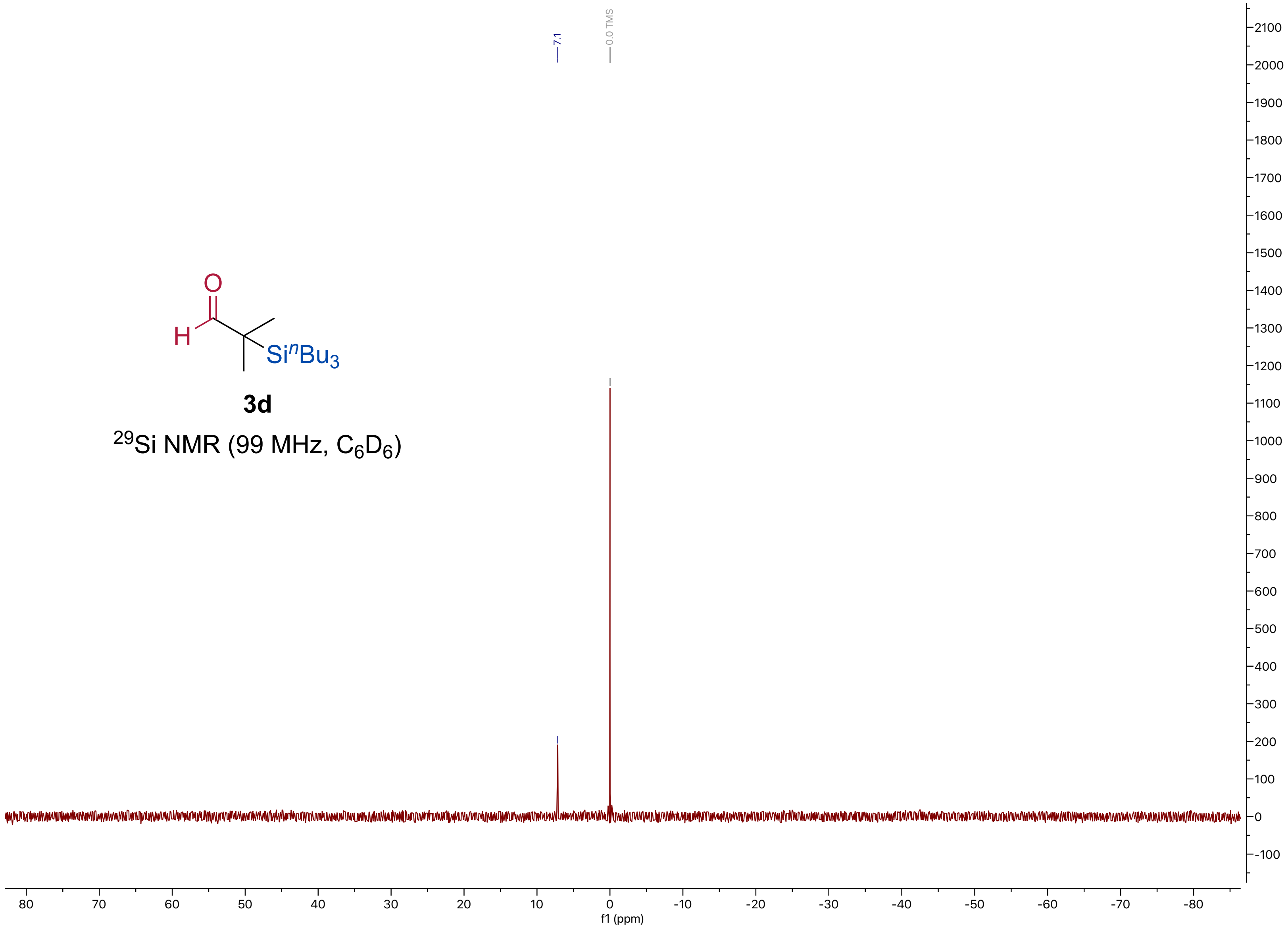

3d

^{29}Si NMR (99 MHz, C_6D_6)

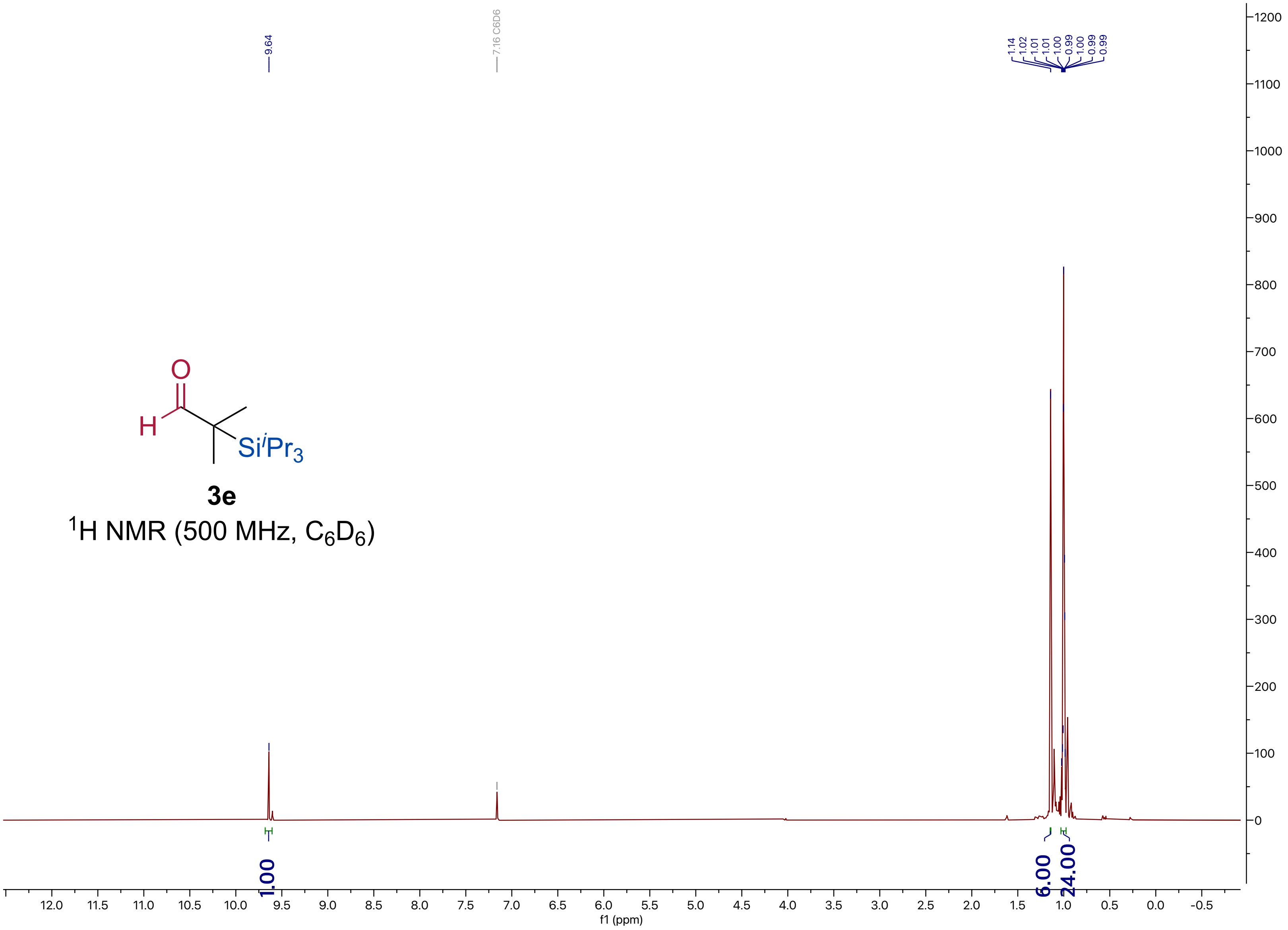

3e

^{1}H NMR (500 MHz, C_6D_6)

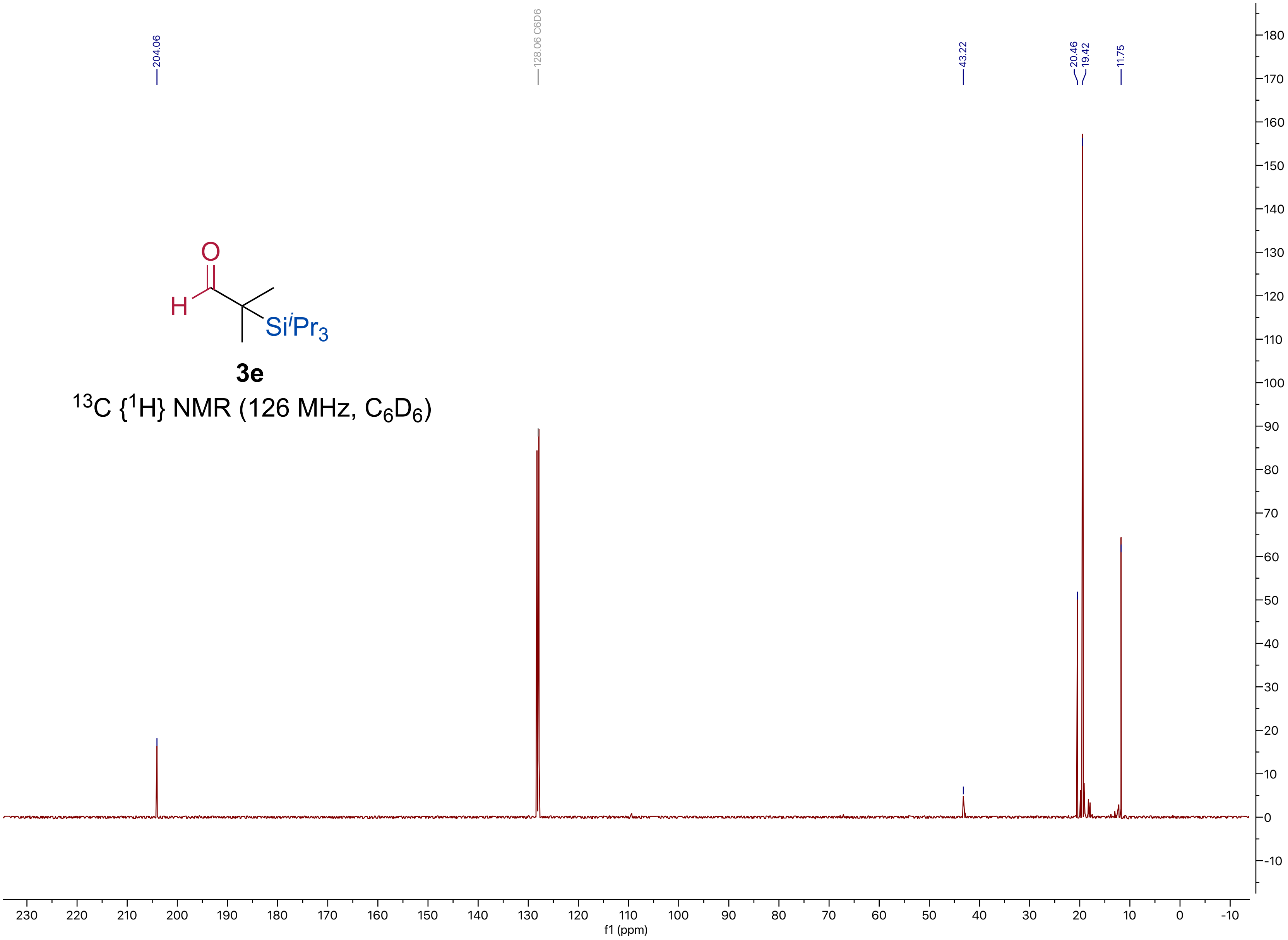

3e

^{13}C {^{1}H} NMR (126 MHz, C_6D_6)

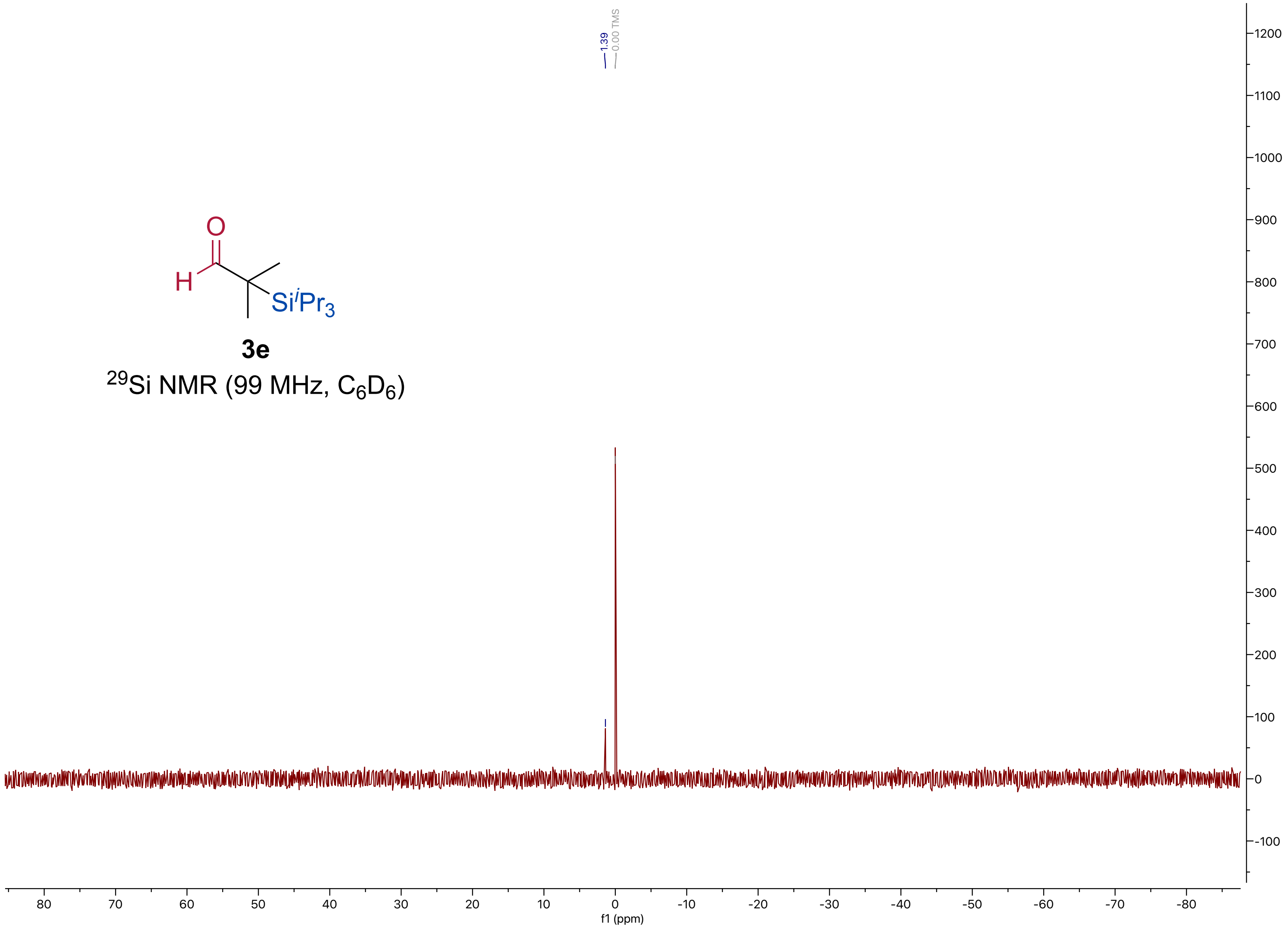

3e

^{29}Si NMR (99 MHz, C_6D_6)

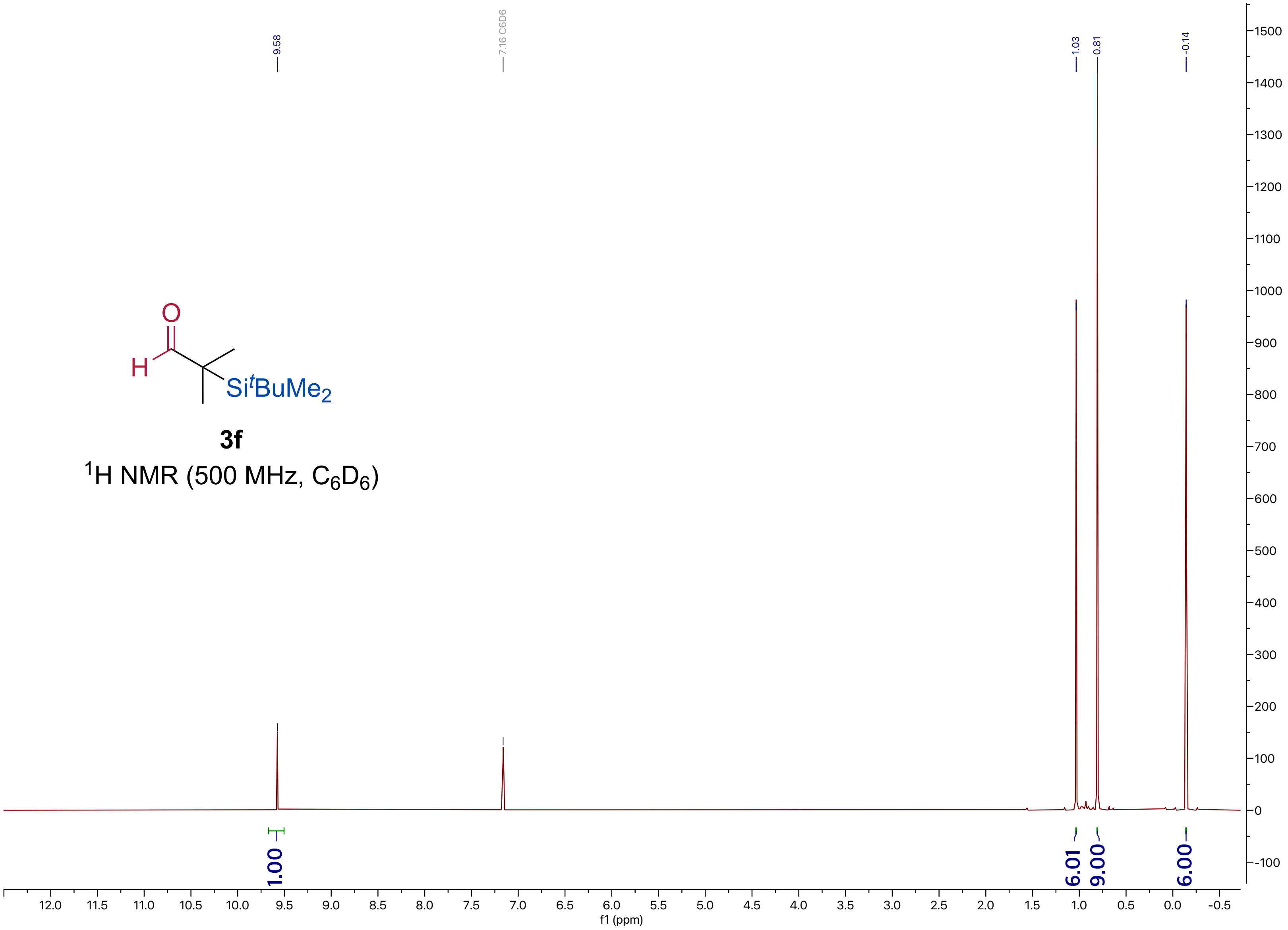

3f

^{1}H NMR (500 MHz, C_6D_6)

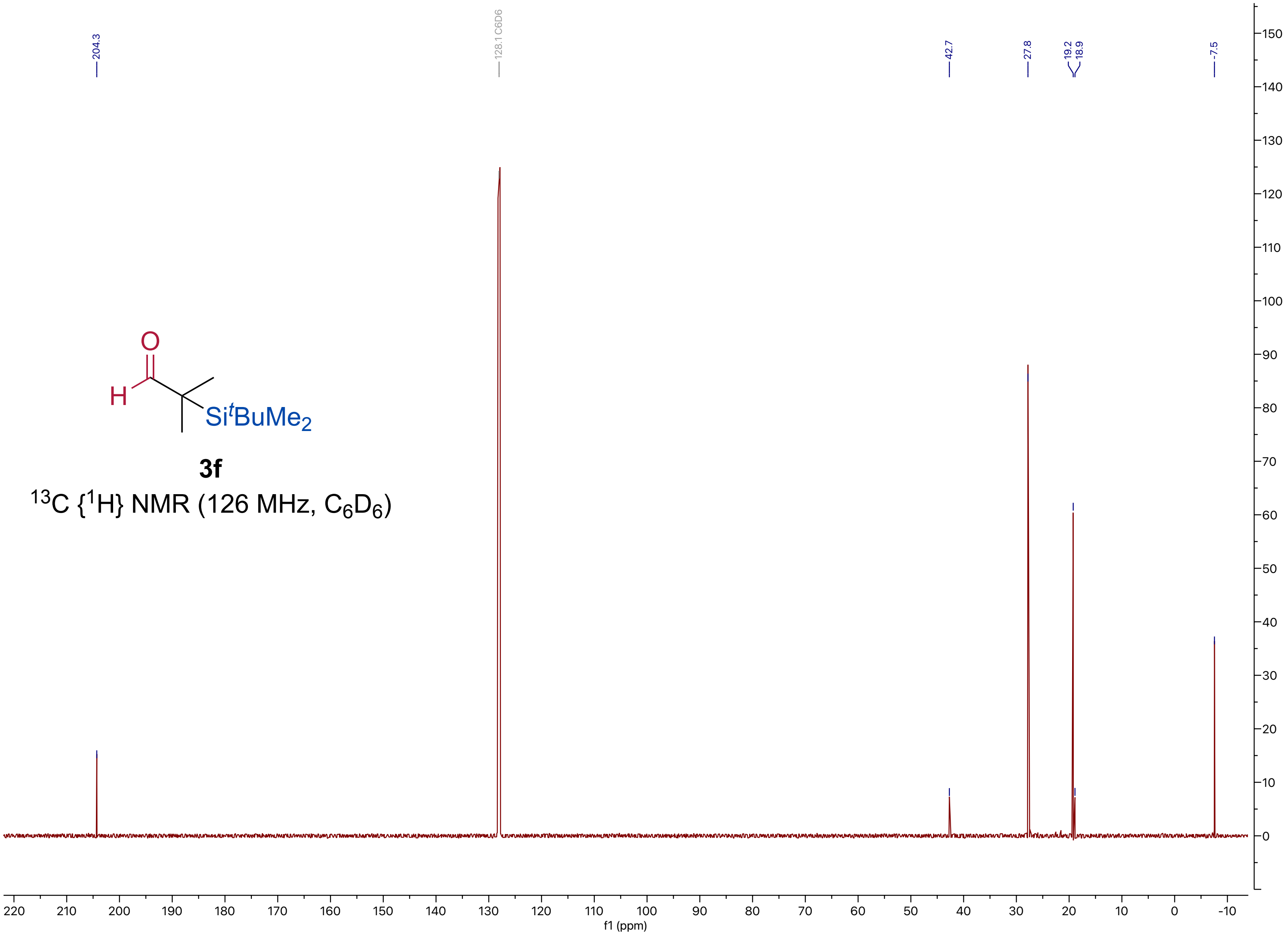

3f

^{13}C {^1H} NMR (126 MHz, C_6D_6)

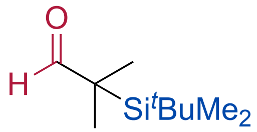

3f

^{29}Si NMR (99 MHz, C_6D_6)

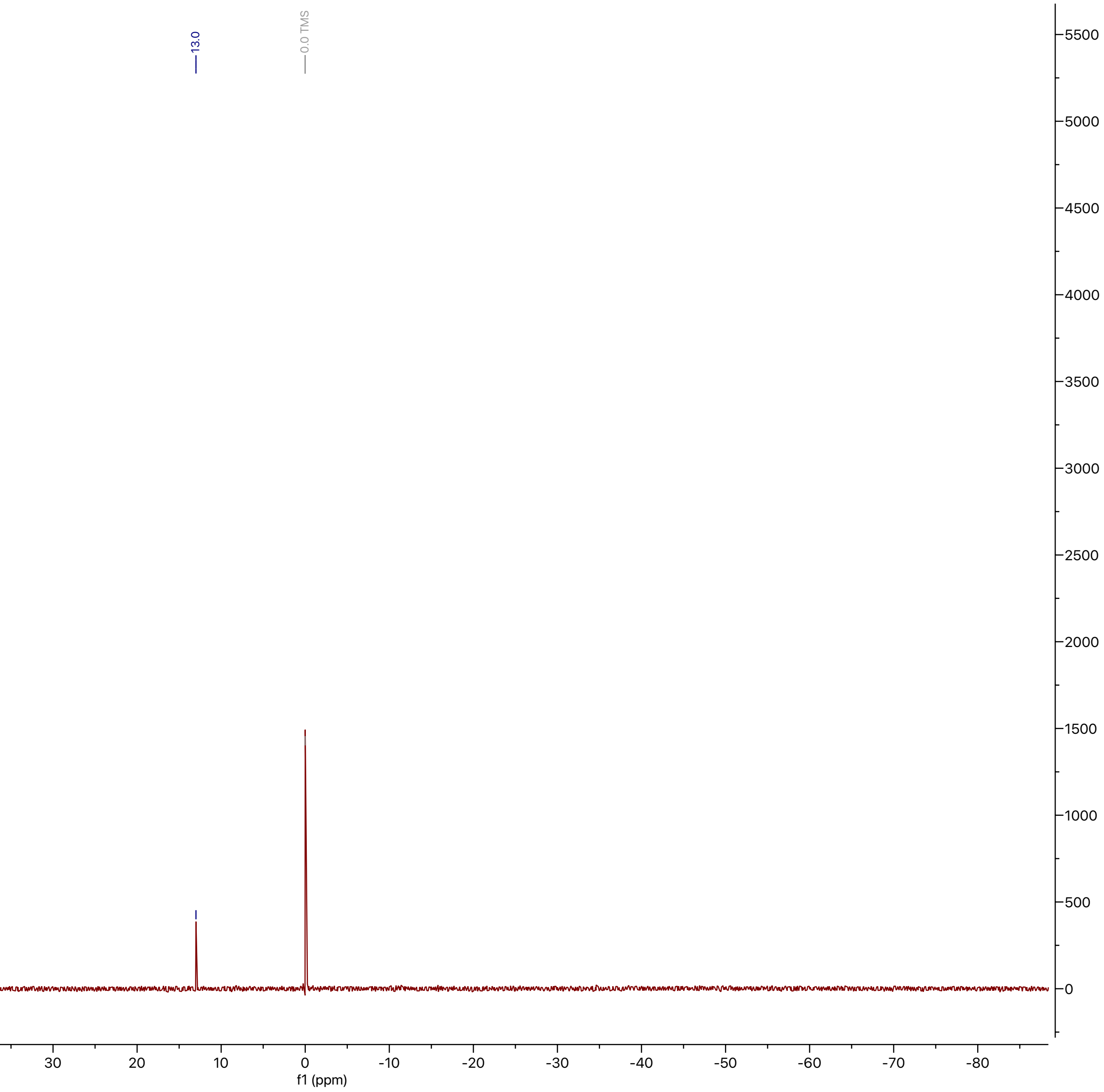

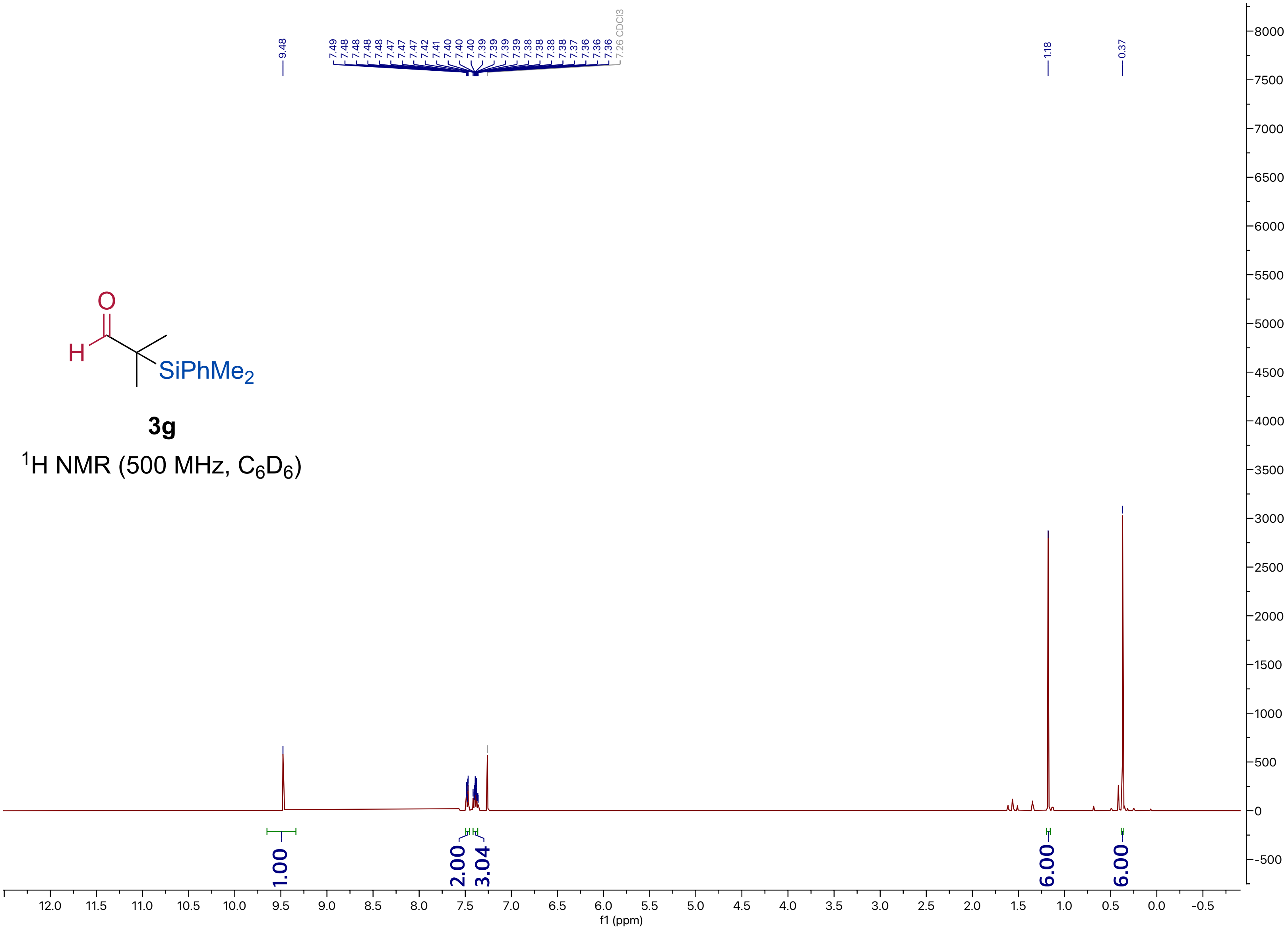

3g

^{1}H NMR (500 MHz, C_6D_6)

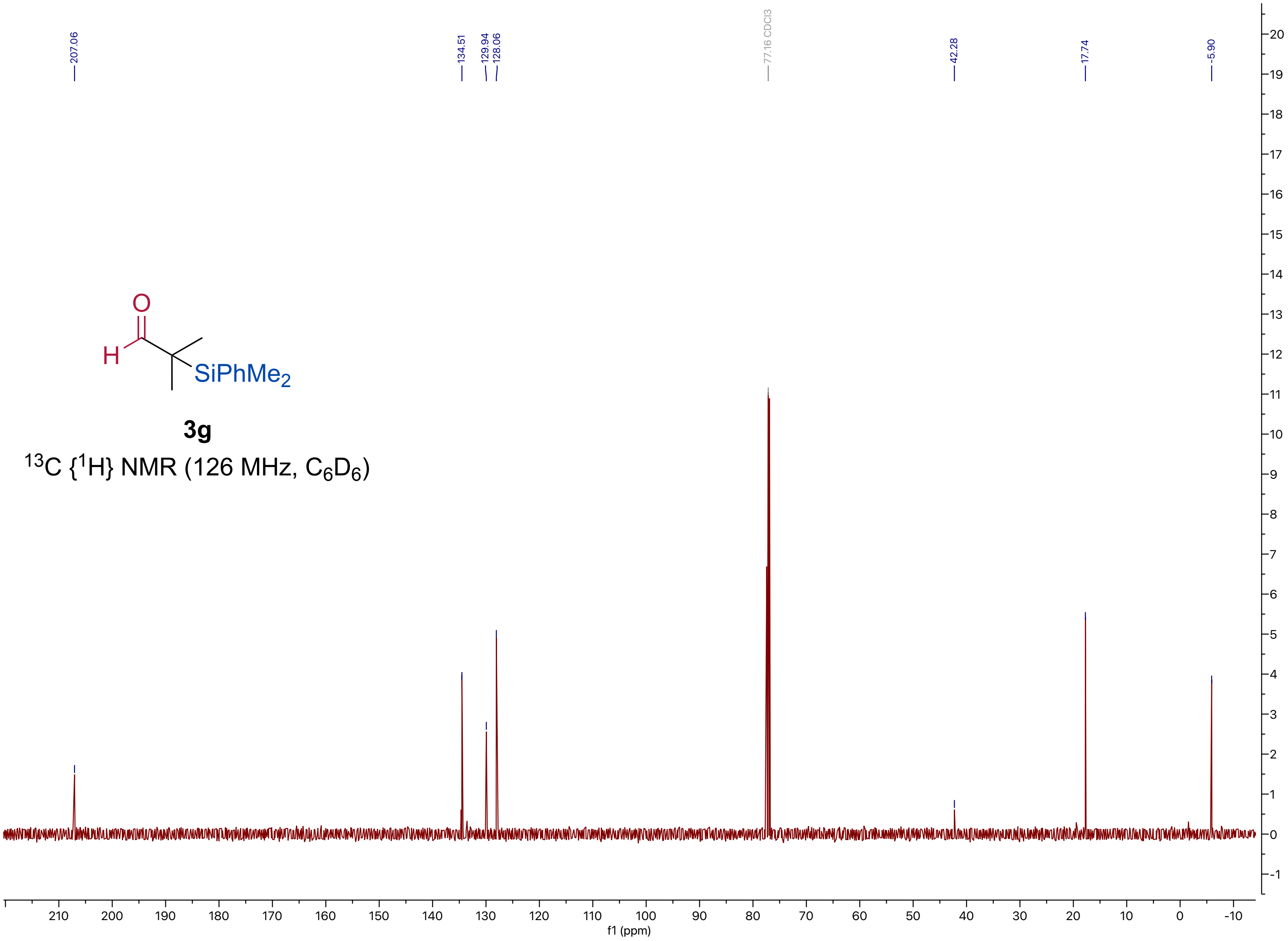

207.06
134.51
129.94
128.06
77.16 CDCl3
42.28
17.74
-5.90
O
H
SiPhMe$_2$
3g
^{13}C {^{1}H} NMR (126 MHz, C_6D_6)
f1 (ppm)

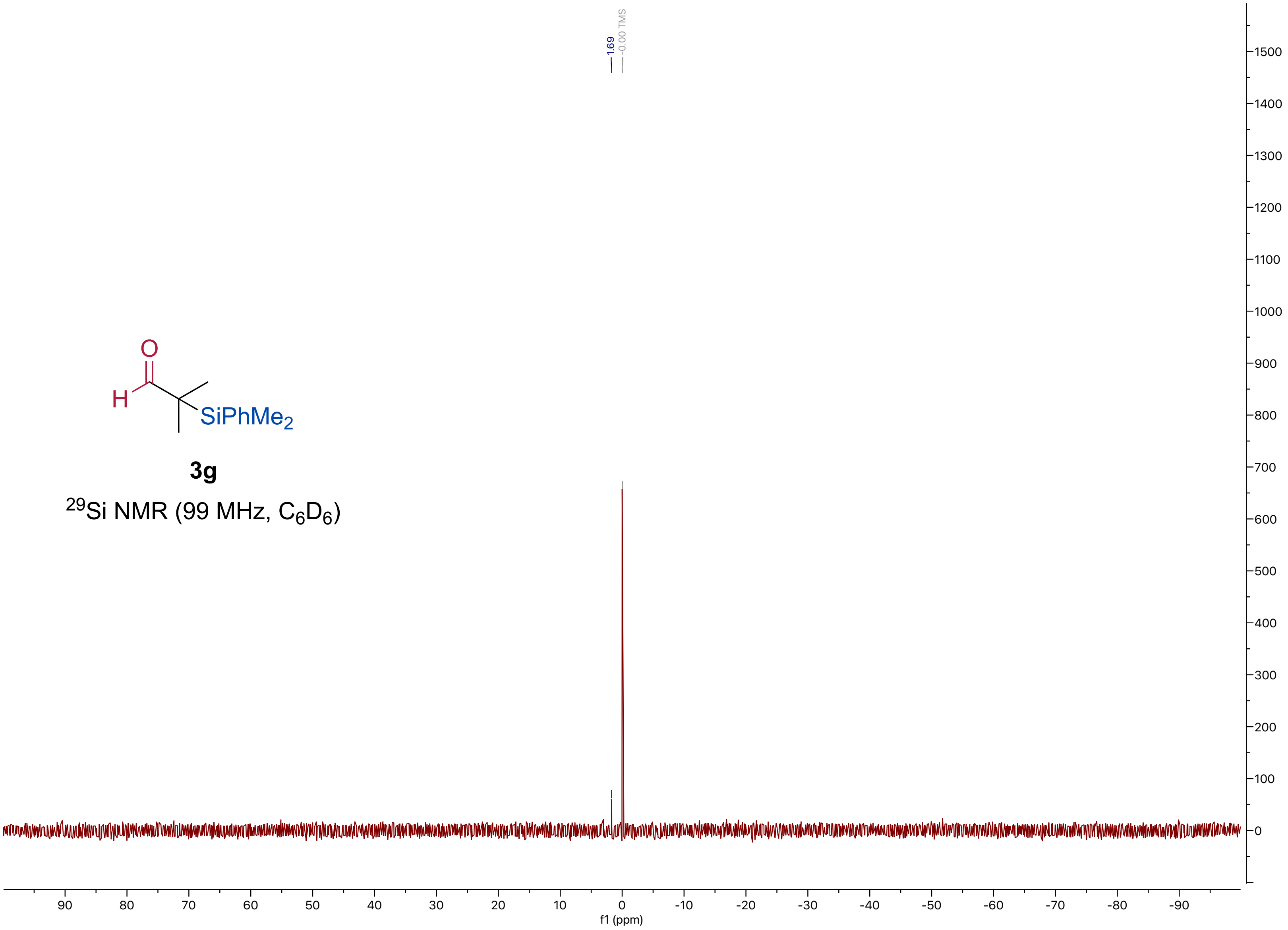

3g

^{29}Si NMR (99 MHz, C_6D_6)

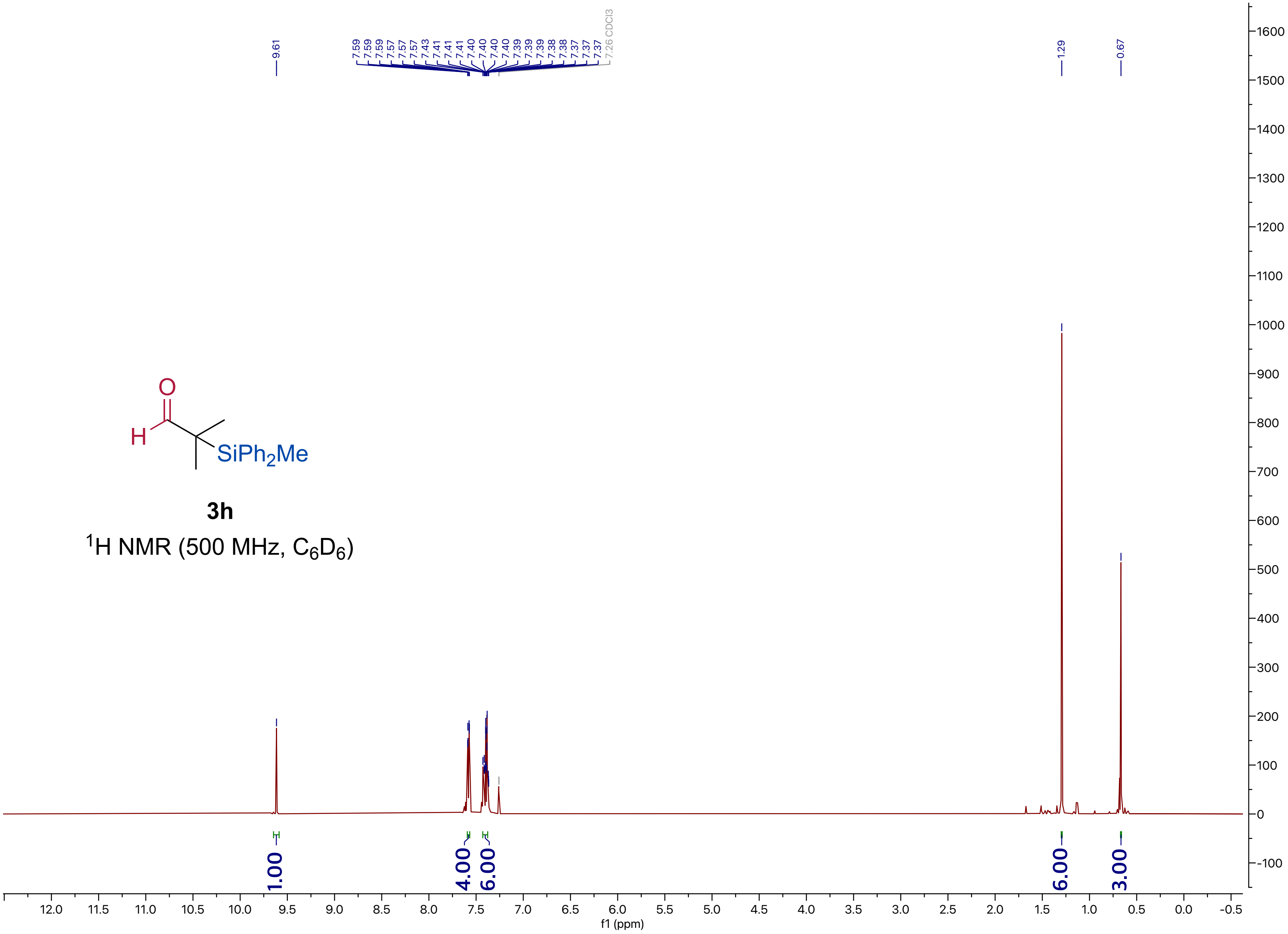

3h

^1H NMR (500 MHz, C_6D_6)

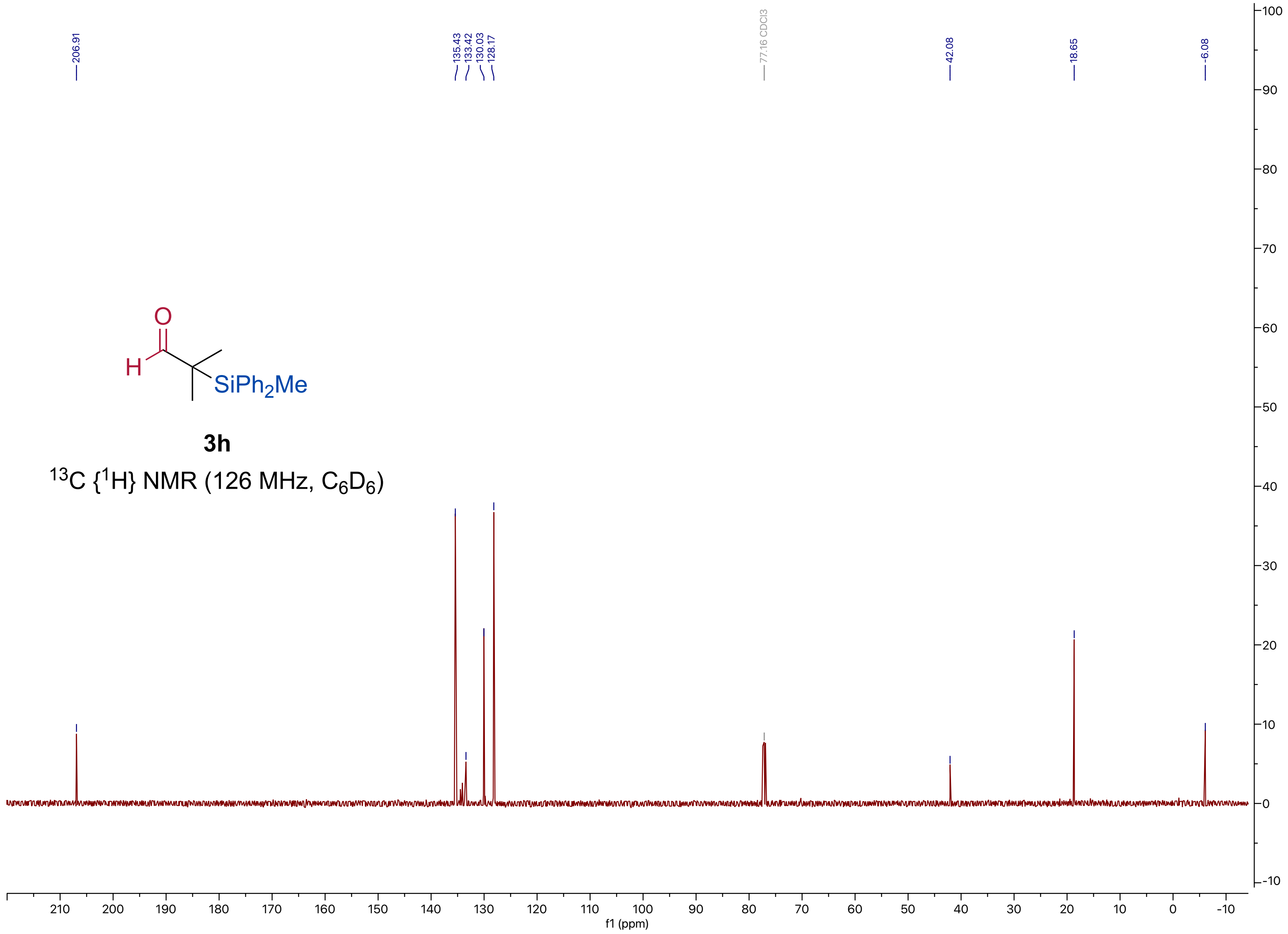

206.91
135.43
133.42
130.03
128.17
77.16 CDCl3
42.08
18.65
-6.08
H
O
SiPh2Me
3h
13C {1H} NMR (126 MHz, C6D6)
f1 (ppm)

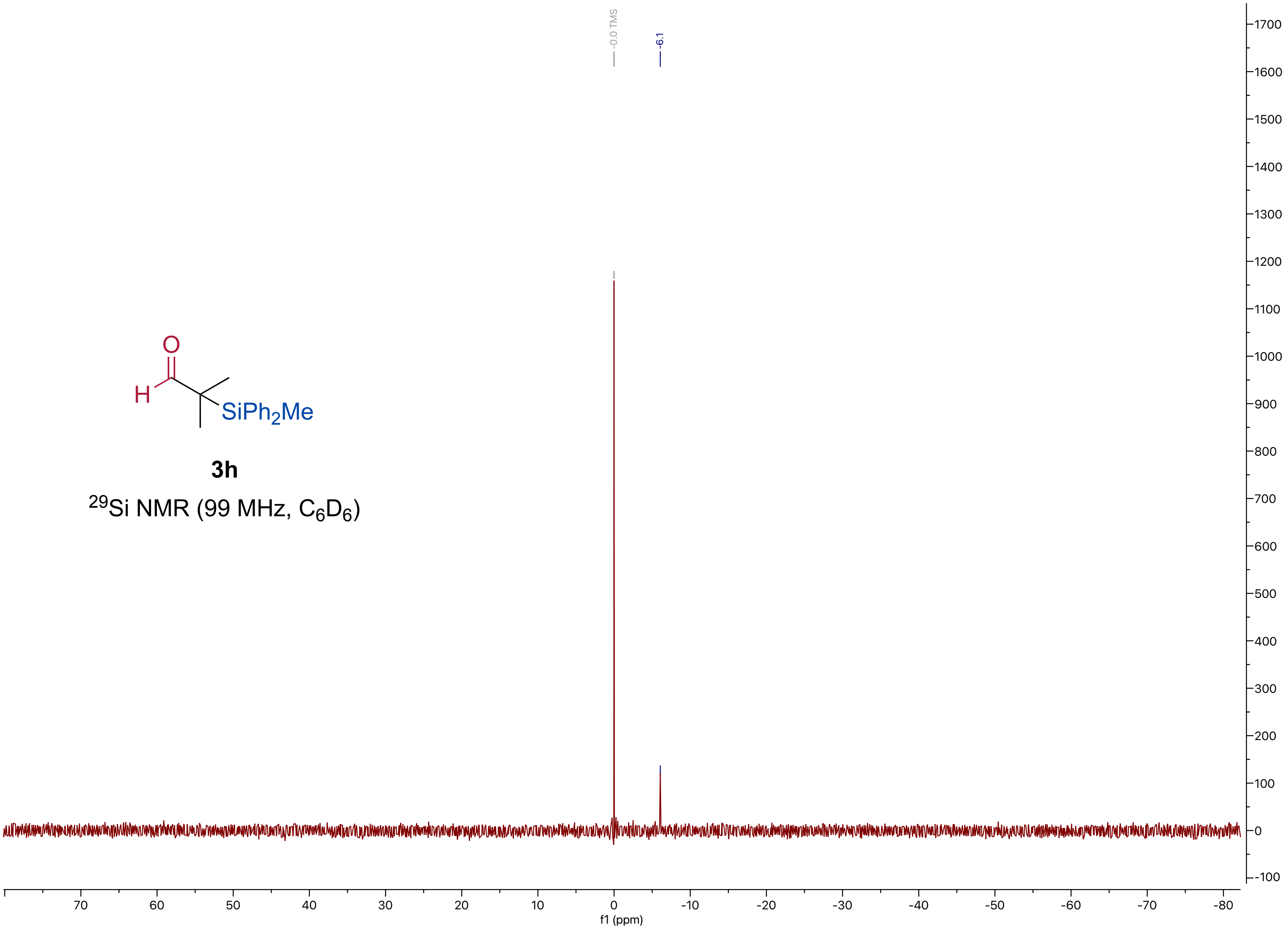

3h

^{29}Si NMR (99 MHz, C_6D_6)

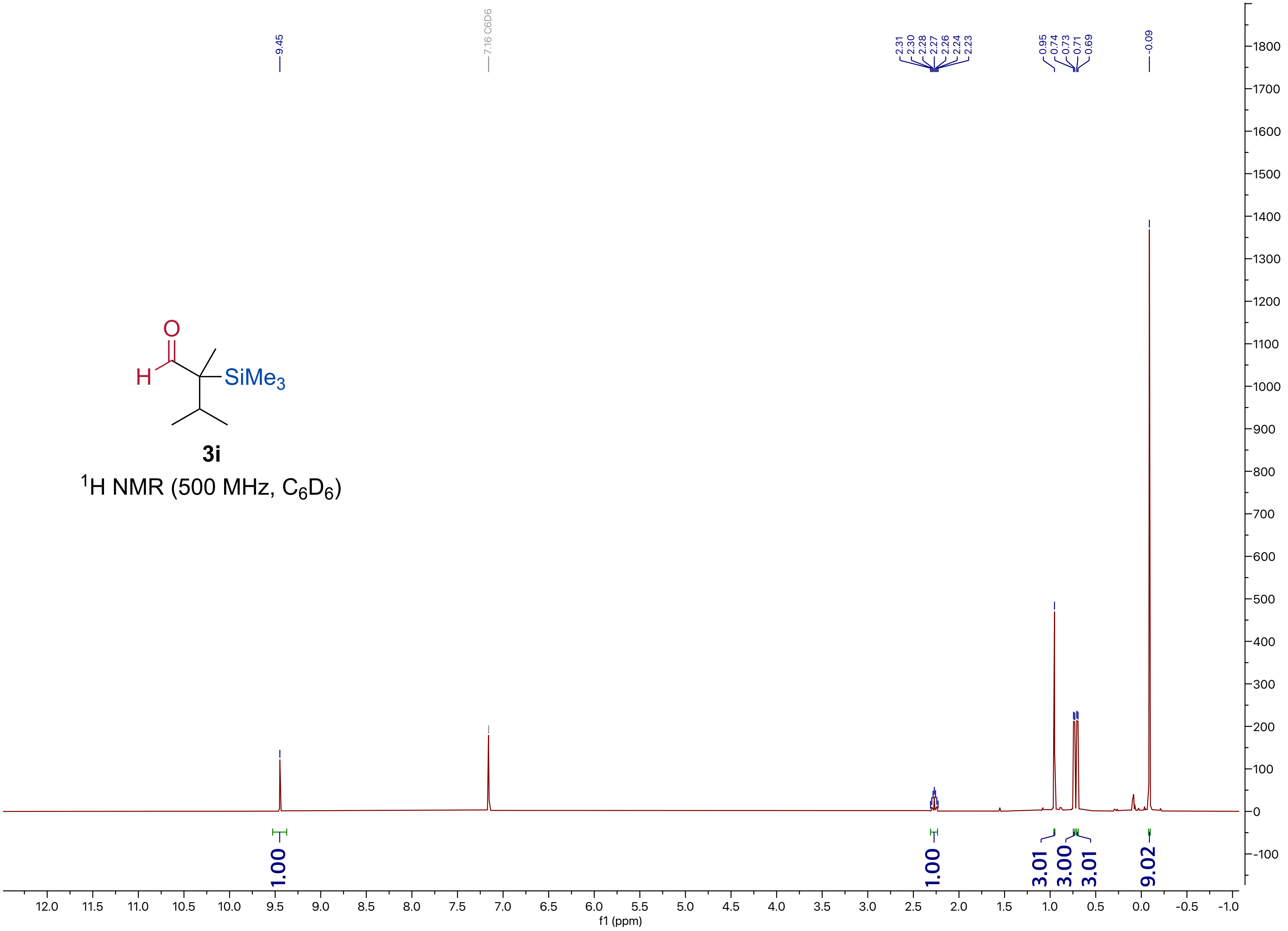
9.45
7.16 C6D6
2.31
2.30
2.28
2.27
2.26
2.24
2.23
0.95
0.74
0.73
0.71
0.69
-0.09
O
H
SiMe3
3i
1H NMR (500 MHz, C6D6)
1.00
1.00
3.01
3.00
3.01
9.02
f1 (ppm)

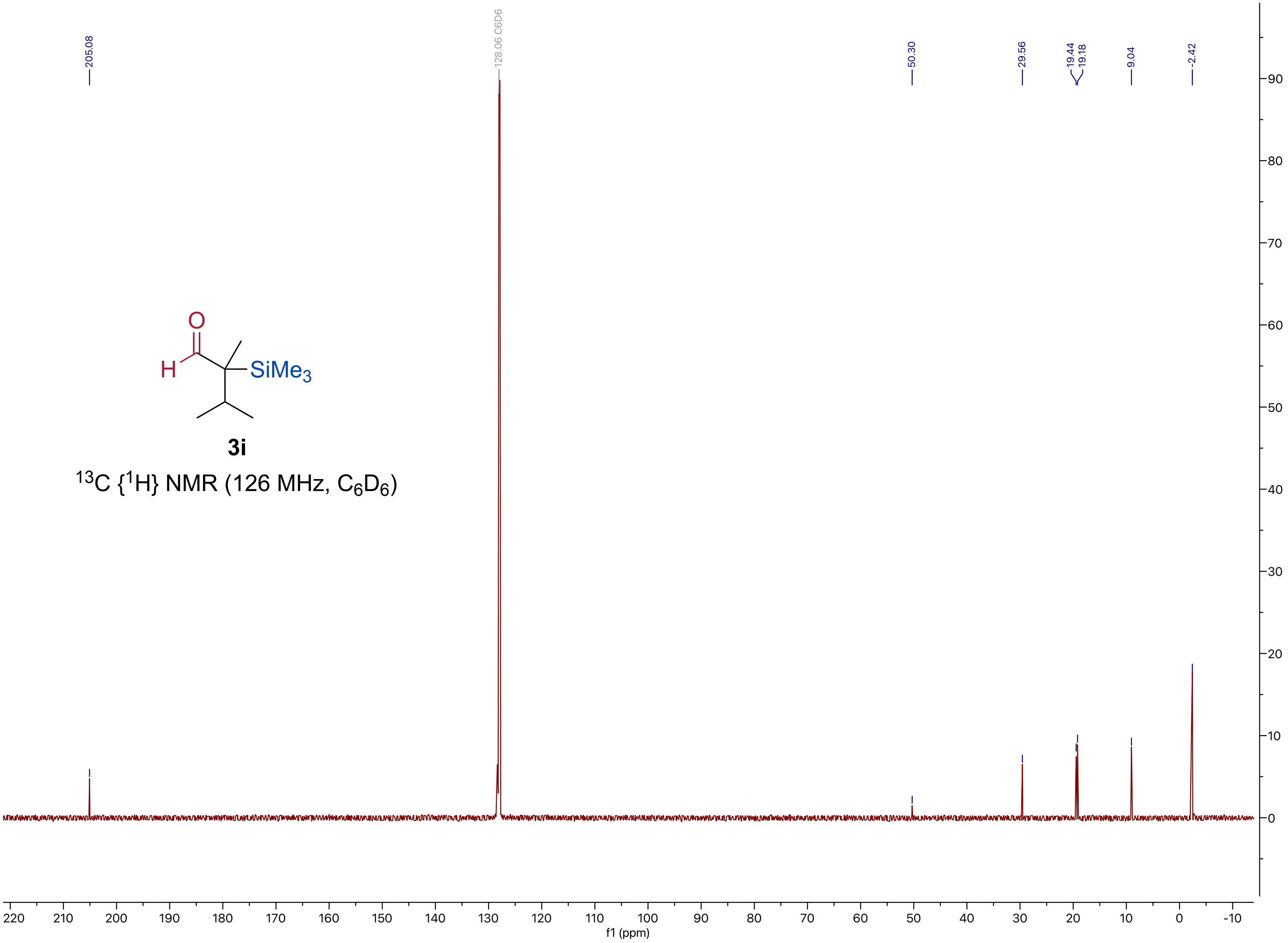

205.08
128.06 C6D6
50.30
29.56
19.44
19.18
9.04
-2.42
O
H
SiMe3
3i
13C {1H} NMR (126 MHz, C6D6)
f1 (ppm)

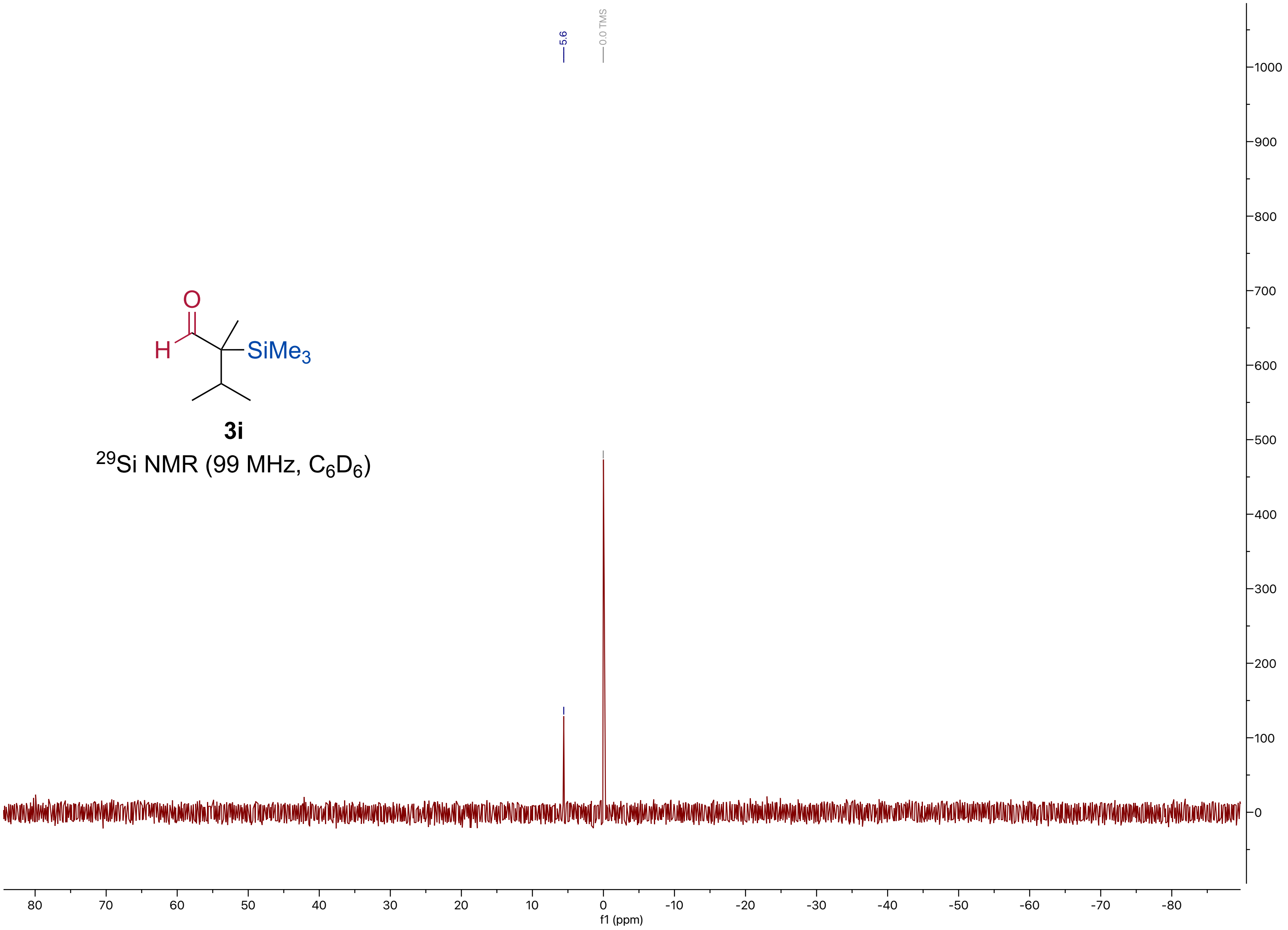

3i

^{29}Si NMR (99 MHz, C_6D_6)

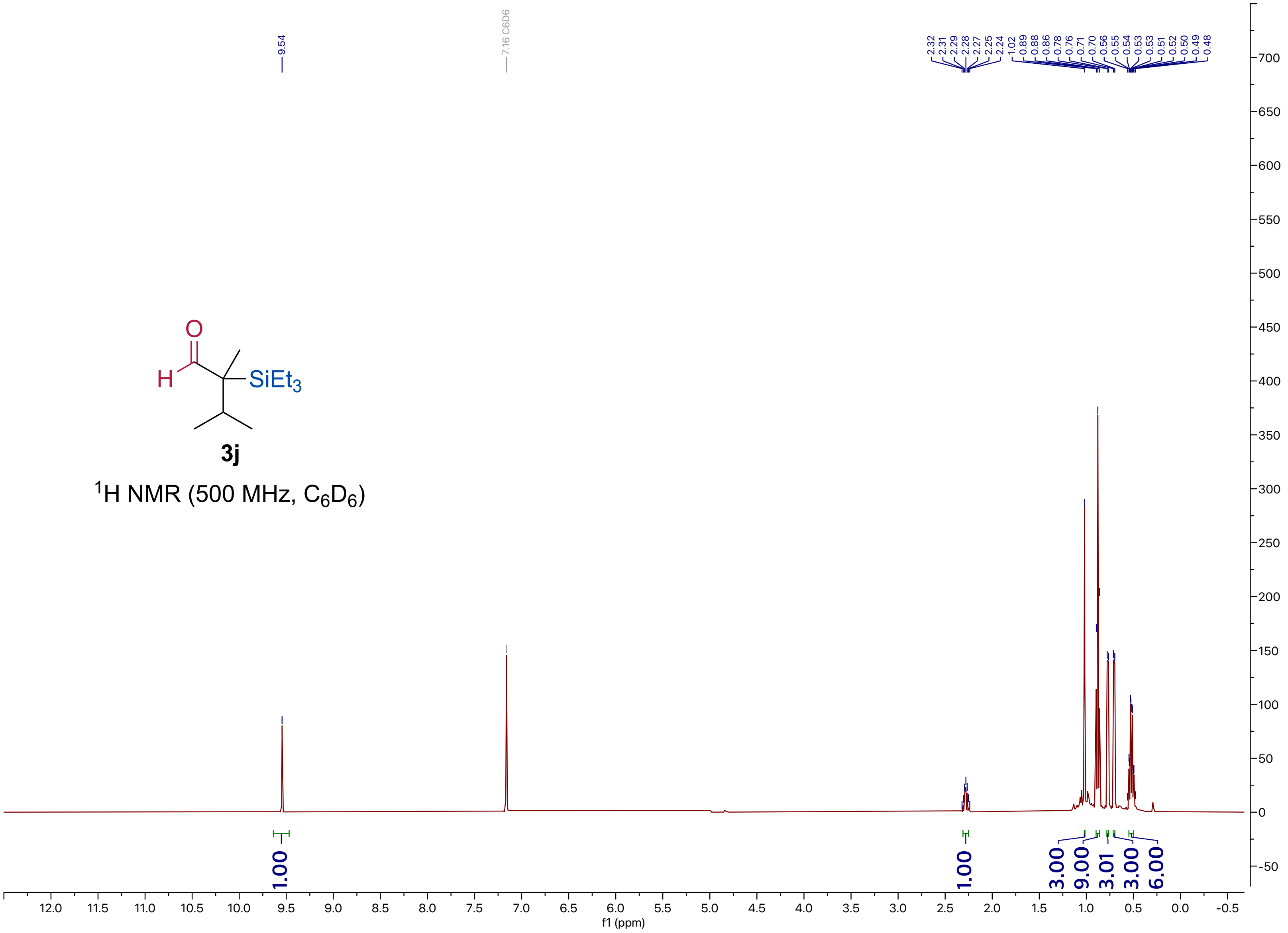

3j

^{1}H NMR (500 MHz, C_6D_6)

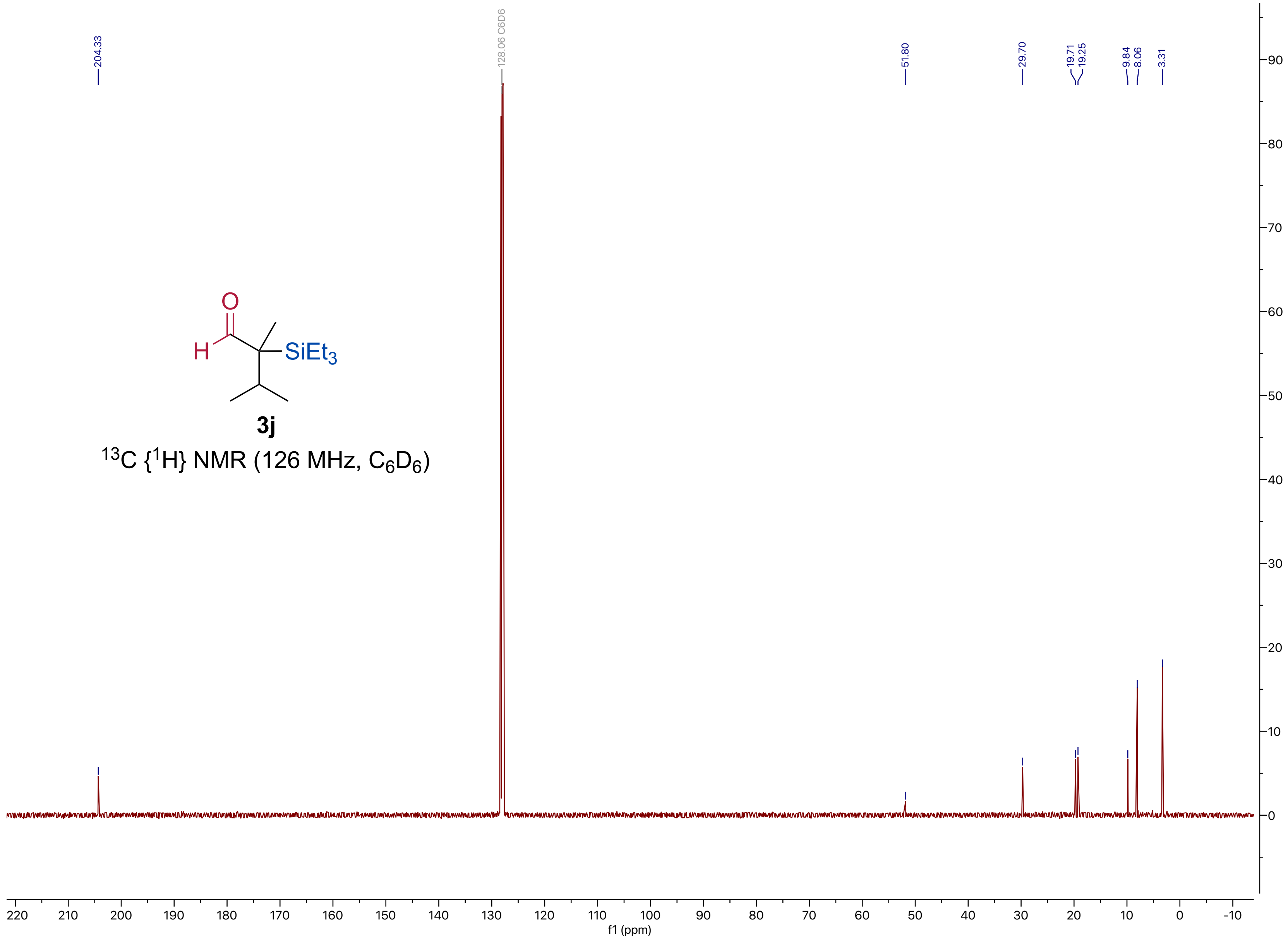

204.33
128.06 C6D6
51.80
29.70
19.71
19.25
9.84
8.06
3.31
H
O
SiEt$_3$
3j
^{13}C {^{1}H} NMR (126 MHz, C_6D_6)
f1 (ppm)

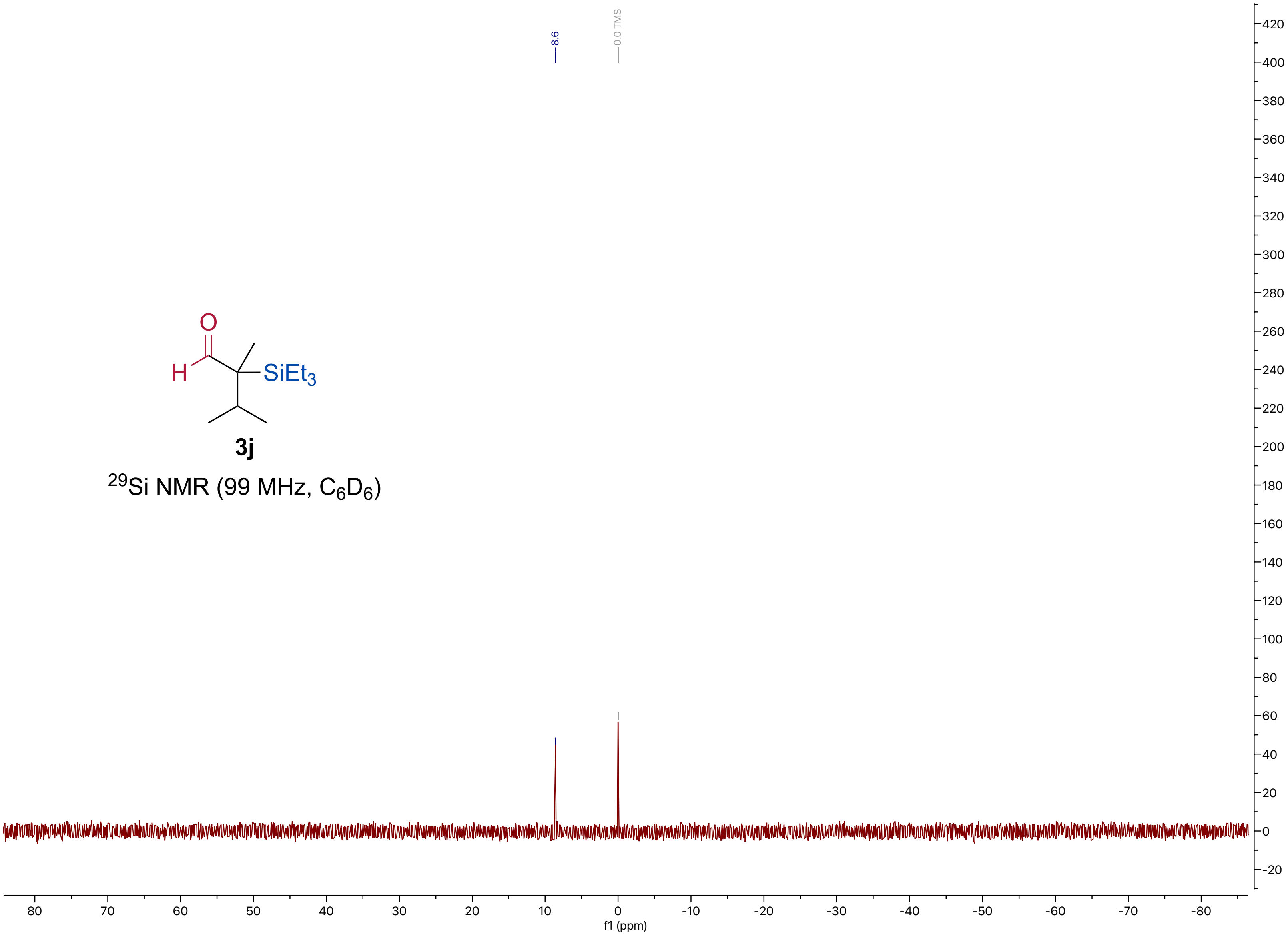

3j

^{29}Si NMR (99 MHz, C_6D_6)

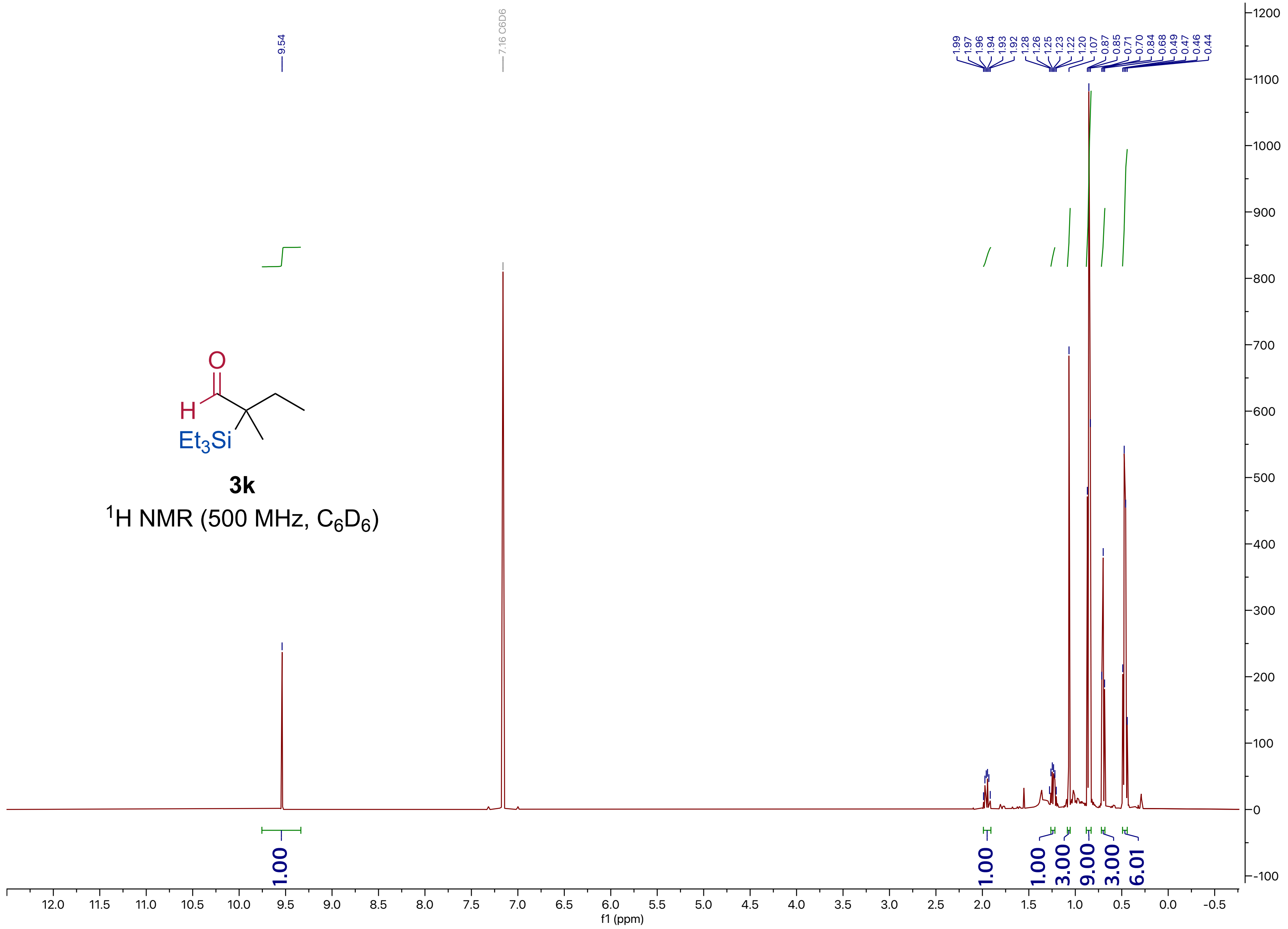
9.54
7.16 C6D6
1.99
1.97
1.96
1.94
1.93
1.92
1.28
1.26
1.25
1.23
1.22
1.20
1.07
0.87
0.85
0.71
0.70
0.84
0.68
0.49
0.47
0.46
0.44
O
H
Et3Si
3k
1H NMR (500 MHz, C6D6)
1.00
1.00
1.00
3.00
9.00
3.00
6.01
f1 (ppm)

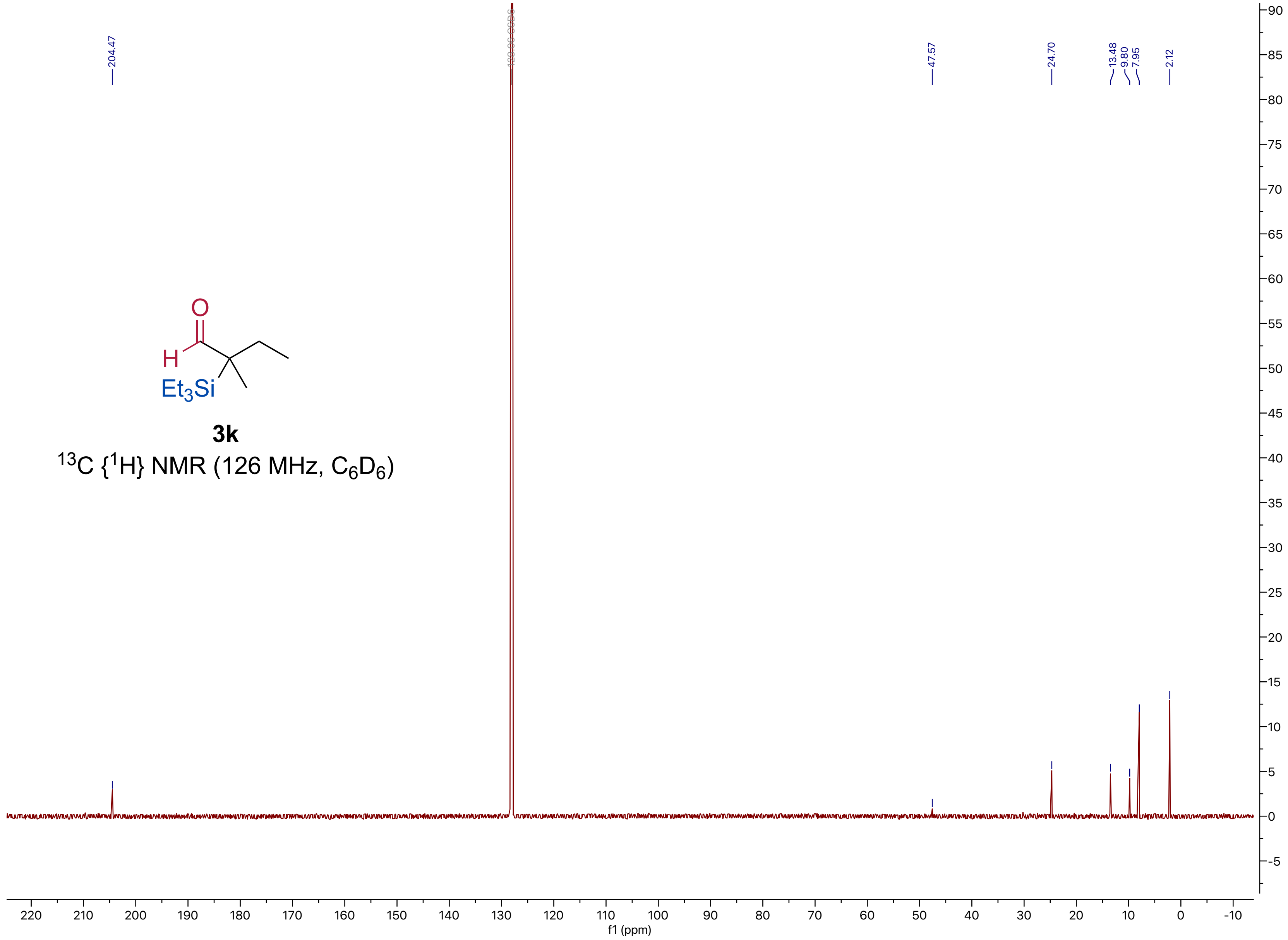

3k

^{13}C {^{1}H} NMR (126 MHz, C_6D_6)

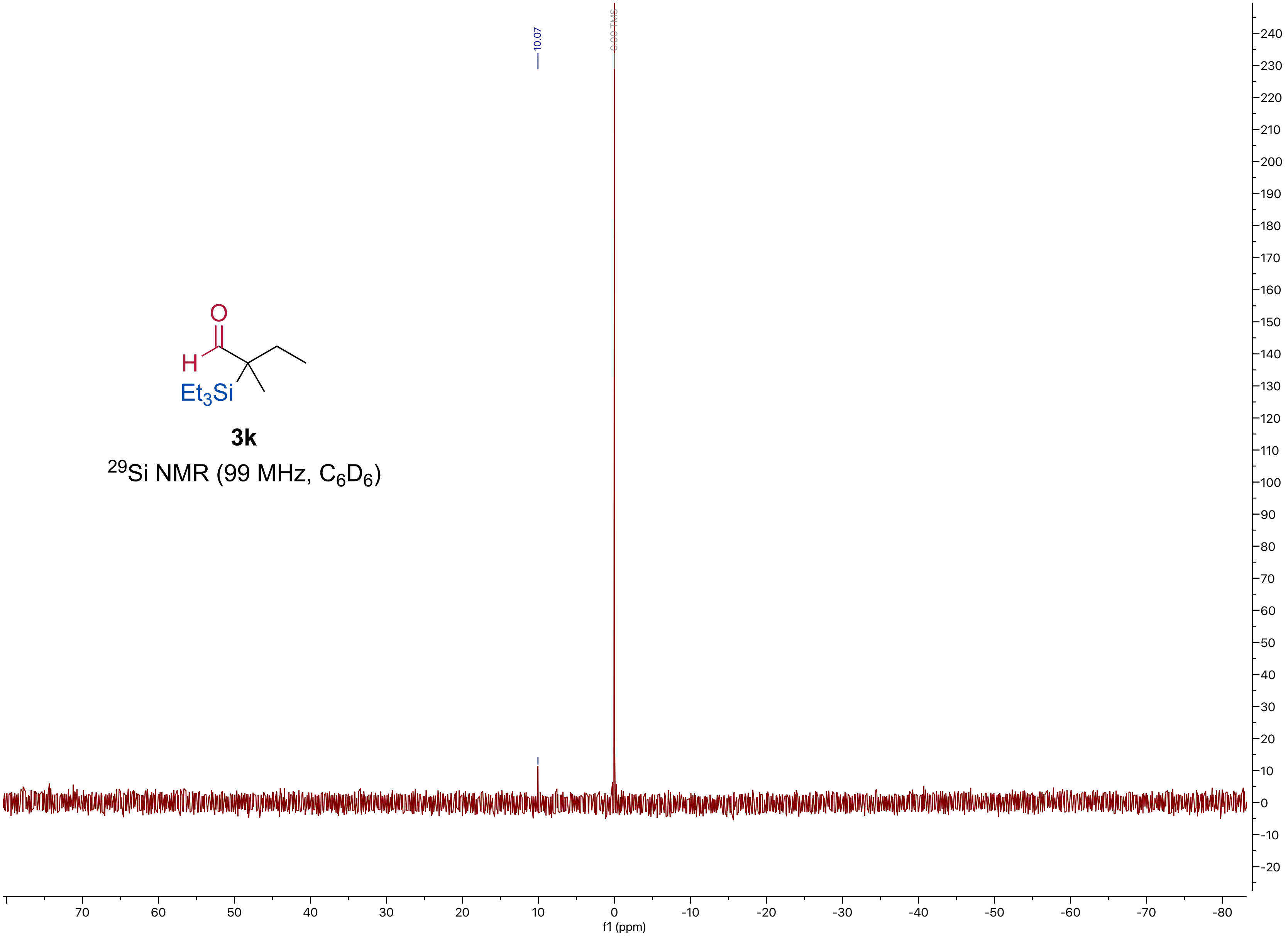

3k

^{29}Si NMR (99 MHz, C_6D_6)

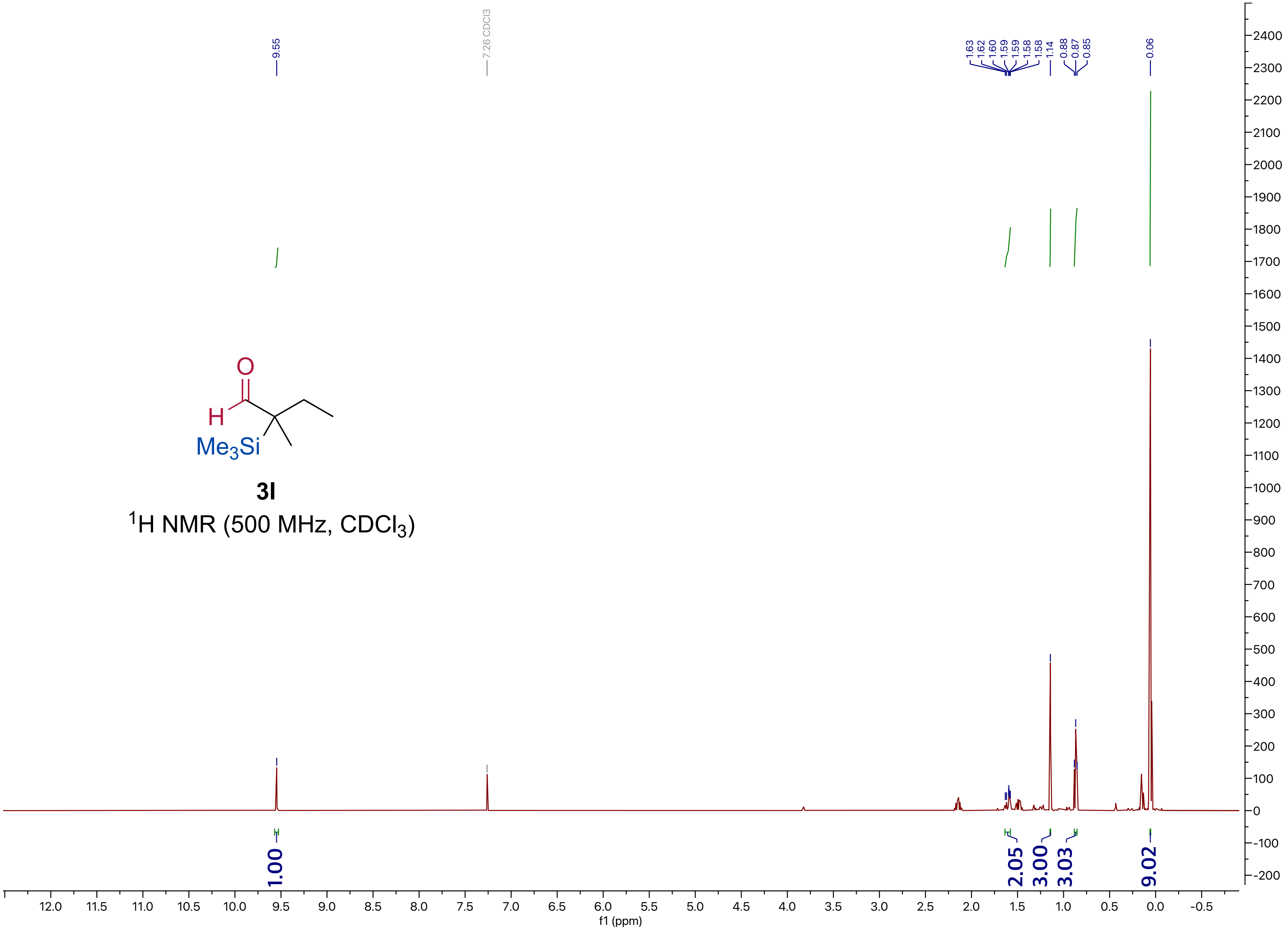

3l

^{1}H NMR (500 MHz, $CDCl_3$)

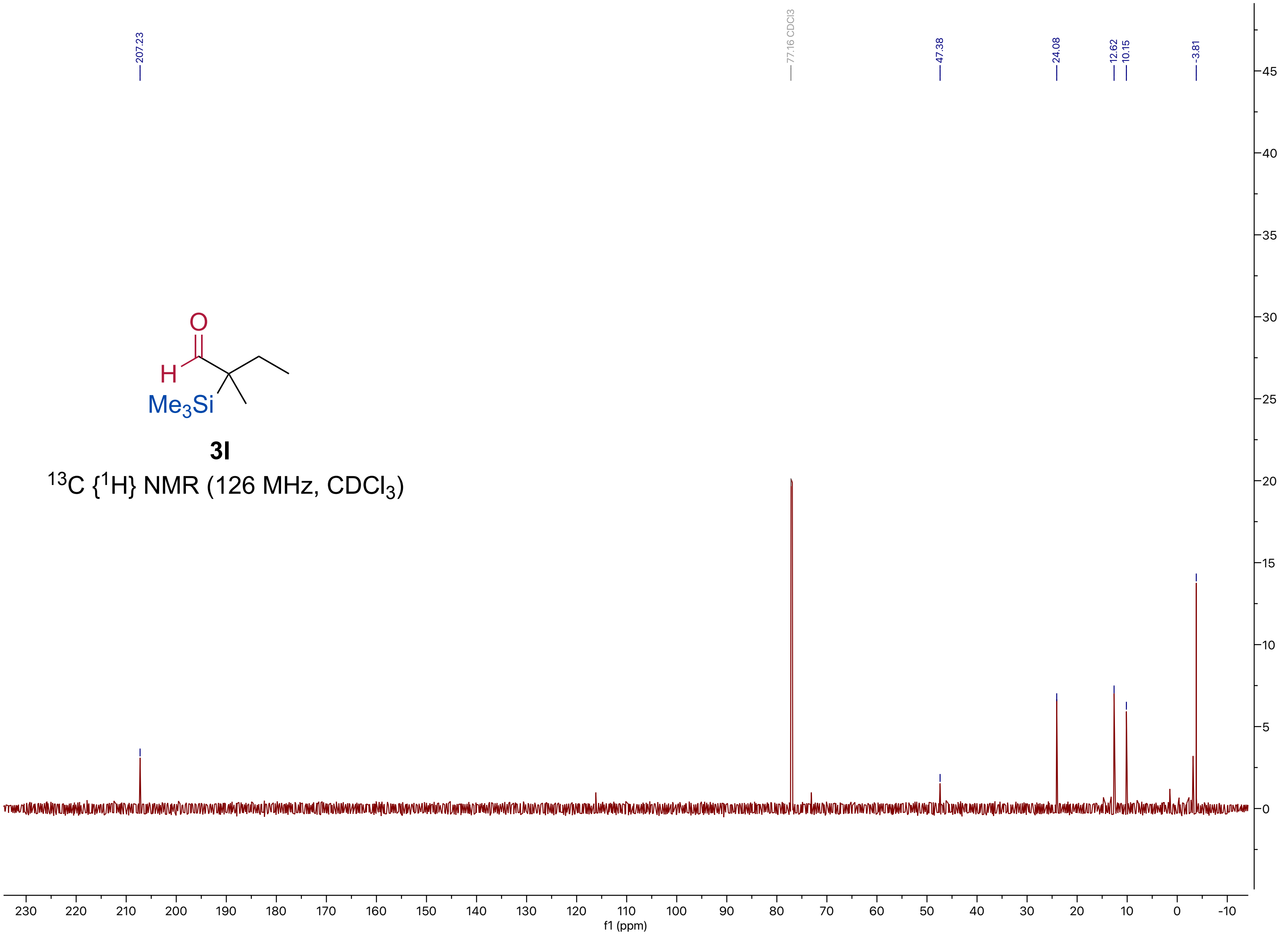

3l

$^{13}C\ \{^{1}H\}$ NMR (126 MHz, $CDCl_3$)

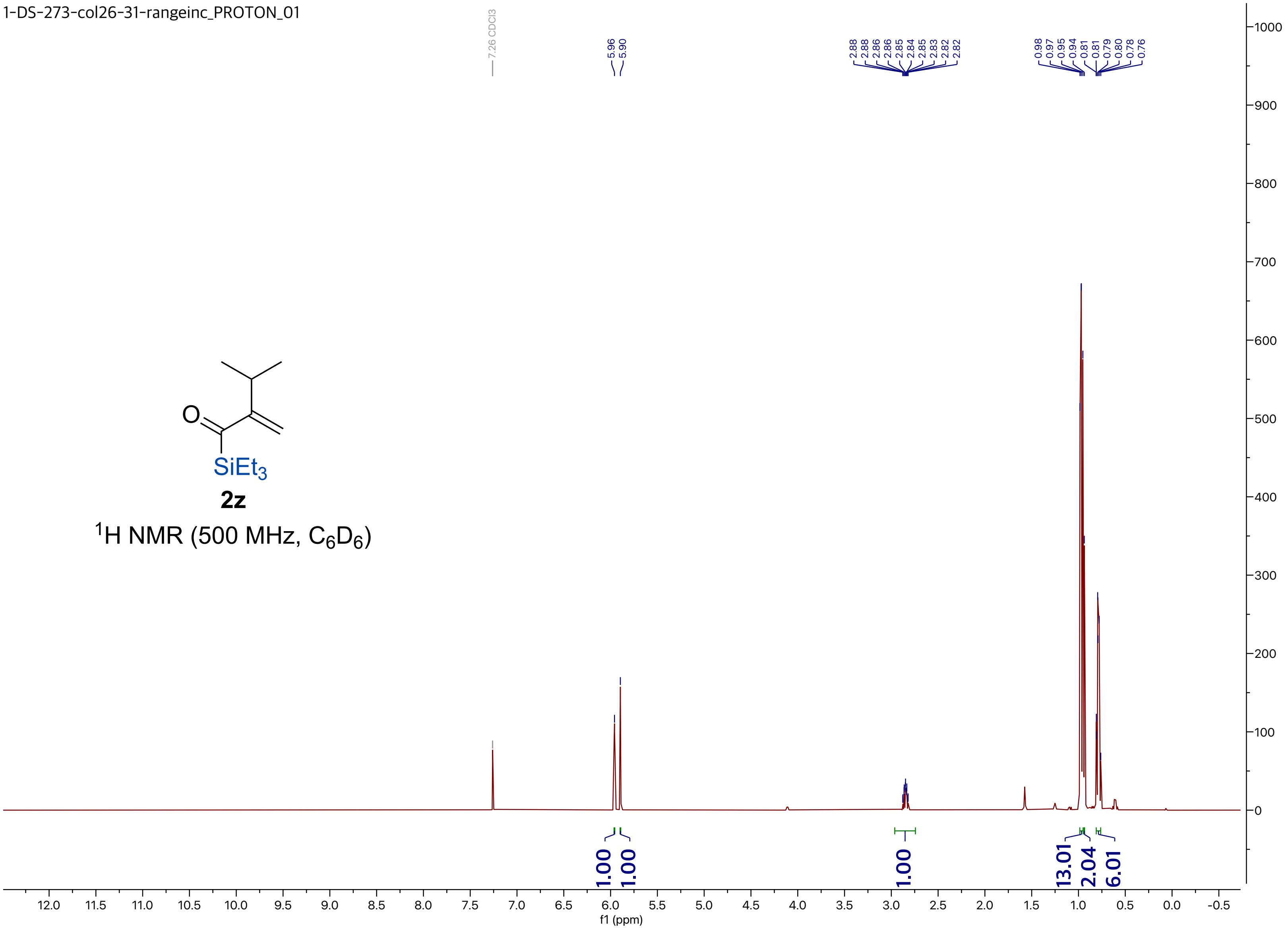
1-DS-273-col26-31-rangeinc_PROTON_01
7.26 CDCl3
5.96
5.90
2.88 2.88 2.86 2.86 2.85 2.84 2.85 2.83 2.82 2.82
0.98 0.97 0.95 0.94 0.81 0.81 0.79 0.80 0.78 0.76
O
SiEt3
2z
1H NMR (500 MHz, C6D6)
1.00
1.00
1.00
13.01
2.04
6.01
f1 (ppm)

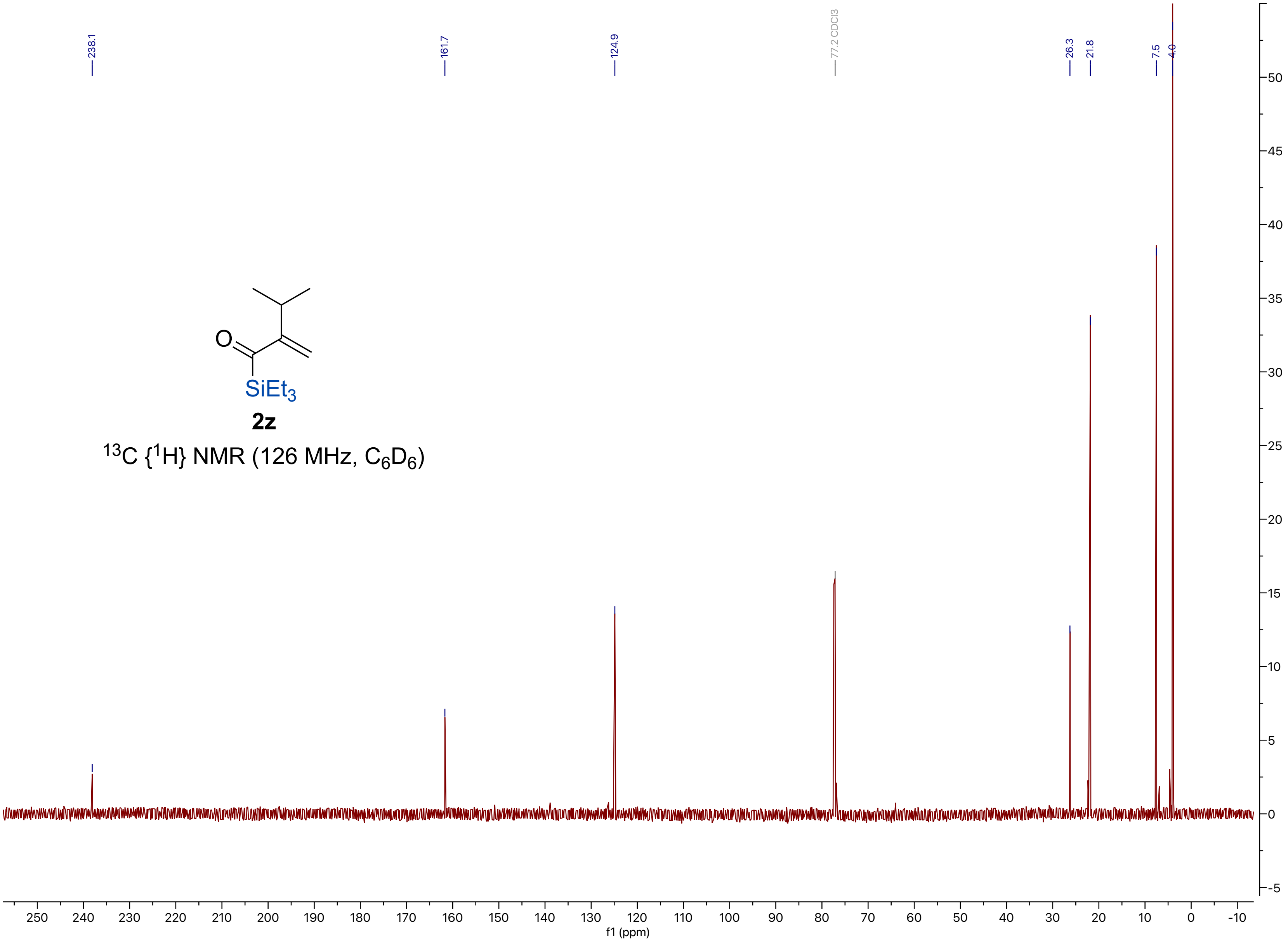

238.1
161.7
124.9
77.2 CDCl3
26.3
21.8
7.5
4.0
O
SiEt3
2z
^{13}C {^{1}H} NMR (126 MHz, C_6D_6)
f1 (ppm)
